# Matching sensor ontologies through siamese neural networks without using reference alignment

**DOI:** 10.7717/peerj-cs.602

**Published:** 2021-06-18

**Authors:** Xingsi Xue, Chao Jiang, Jie Zhang, Hai Zhu, Chaofan Yang

**Affiliations:** 1 Fujian Provincial Key Laboratory of Big Data Mining and Applications, Fujian University of Technology, Fuzhou, Fujian, China; 2 School of Computer Science and Engineering, Yulin Normal University, Yulin, Guangxi, China; 3 School of Network Engineering, Zhoukou Normal University, Zhoukou, Henan, China; 4 Intelligent Information Processing Research Center, Fujian University of Technology, Fuzhou, Fujian, China

**Keywords:** Sensor Ontology Matching, Siamese Neural Networks, Alignment Refinement

## Abstract

Sensors have been growingly used in a variety of applications. The lack of semantic information of obtained sensor data will bring about the heterogeneity problem of sensor data in semantic, schema, and syntax levels. To solve the heterogeneity problem of sensor data, it is necessary to carry out the sensor ontology matching process to determine correspondences among heterogeneous sensor concepts. In this paper, we propose a Siamese Neural Network based Ontology Matching technique (SNN-OM) to align the sensor ontologies, which does not require the utilization of reference alignment to train the network model. In particular, a representative concepts extraction method is presented to enhance the model’s performance and reduce the time of the training process, and an alignment refining method is proposed to enhance the alignments’ quality by removing the logically conflict correspondences. The experimental results show that SNN-OM is capable of efficiently determining high-quality sensor ontology alignments.

## Introduction

Over the past decades, sensors have been growingly used in a variety of applications, e.g., medical science, space observation, wildfire detection, traffic management, and weather forecasting (Yawut & Kilaso, 2011; Doolin & Sitar, 2005; Topol, Steinhubl & Torkamani, 2015; Chen, 2018; Sheth, Henson & Sahoo, 2008; Gravina et al., 2017; Du et al., 2020; Chu et al., 2020). In order to make diverse kinds of sensors cooperate in the common task of detecting and identifying a large number of observation data, it is necessary to combine sensor networks with database and Web techniques, which is called Sensor Web (Corcho & García-Castro, 2010; Xue & Chen, 2020). However, the lack of semantic information on obtained sensor data might bring about the heterogeneity problem of sensor data in semantic, schema, and syntax levels. In order to address this problem, the Semantic Sensor Web (SSW) has emerged, whose kernel technique is the sensor ontology. Sensor ontology can be used to annotate the sensor observation data and realize different sensor applications’ interoperability (Xue & Chen, 2020), but there also exists the heterogeneity issue among different sensor ontologies. The sensor ontology matching technique is able to determine the correspondences between different sensor concepts and bridge the semantic gap between two heterogeneous sensor ontologies.

Matching sensor ontologies manually is a tedious, time-consuming, and error-prone task. Hence, evolutionary algorithms (EAs) and the machine learning (ML) (Chen, 2020; Chen et al., 2020; Chen, Hwang & Kung, 2019; Lin et al., 2021; Lin et al., 2019) based ontology matching techniques (OMTs) have become popular methodology for determining the ontology alignments (Doan et al., 2004; Nezhadi, Shadgar & Osareh, 2011; Khoudja, Fareh & Bouarfa, 2018b). As the complex nature of sensor ontology matching process, recently, the neural network (NN) becomes the popular technique to align the sensor ontologies (Khoudja, Fareh & Bouarfa, 2018b; Khoudja, Fareh & Bouarfa, 2018a; Bento, Zouaq & Gagnon, 2020; Jiang & Xue, 2020; Iyer, Agarwal & Kumar, 2020). However, the existing NN-based OMTs require the utilization of reference alignment, which is unavailable in the real-world matching task. In addition, their model training process needs long runtime, which also hampers their applications. To overcome these drawbacks, this work proposes a Siamese neural network based ontology matching technique (SNN-OM) to effectively and efficiently align the sensor ontologies. The main contributions made in this paper are listed in the following:

• A Siamese neural network is proposed to align the sensor ontologies, which enhances the performance without using the reference alignment.
• A representative concepts extraction method is presented to enhance the model’s performance and reduce the time of training process.
• A feature usage method of sensor concept’s context information is proposed to improve the alignment’s quality.
• An alignment refining method is proposed to enhance the alignment’s quality, which makes use of the sensor ontology’s concept hierarchy to remove the logically conflict correspondences.

The rest of the paper is organized as follows: ‘Related Work’ provides the related work on state-of-the-art ML-based OMTs. ‘Preliminary’ presents the definition of sensor ontology, sensor ontology matching, and the alignment’s evaluation metrics, and introduces the proposed CSM. ‘Siamese Neural Network Based Ontology Matching Technique’ gives the detail of SNN-OM, including the extraction of representative concepts, training of the model, matching process, and alignment refinement. ‘Experiment’ shows the experiments on OAEI’s benchmark and three pairs of sensor ontologies. ‘Conclusion’ draws the conclusion.

## Related Work

Many evolutionary algorithms (EAs)-based OMTs have been proposed to tackle the ontology matching problem, partitioned into two categories, i.e., single-objective evolutionary algorithms (SOEAs) and multi-objective evolutionary algorithms (MOEAs). Naya et al. (2010) proposed an SOEA-based OMT, which combines a quantity of concept similarity measures (CSMs) and proposed an effective encoding mechanism. After that, a hybrid SOEA-based OMT is further proposed (Xue & Wang, 2015), which introduced the local search algorithm into the evolution process of genetic algorithm to improve the performance of the algorithm. Jiang & Xue (2021) proposed a uniform compact genetic algorithm (UCGA)-based OMT, which is able to reduce the runtime and memory consumption. In order to meet the various needs of different decision makers, many MOEAs have been developed to solve the ontology matching problem to provide a set of solutions called the Pareto set. An improved nondominated sorting genetic algorithm (iNSGA-II)-based OMT (Huang, Xue & Jiang, 2020) is proposed to improve the alignment quality, which introduced a local perturbation algorithm (Meng & Pan, 2016) into the evolution process of NSGA-II.

A semi-automatic ML-based OMT, GLUE (Doan et al., 2004), has been proposed, which is able to create highly accurate alignment that Concept Similarity Measures (CSMs) are expressed by the joint probability distribution of concepts involved. Nezhadi, Shadgar & Osareh (2011) proposed an ML-based OMT, which combined several CSMs to enhance the matching results’ quality and regarded ontology matching as the regression problem. In addition, for the efficiency of ontology matching, several ML algorithms have been studied by them, i.e., decision tree (DT), AdaBoost, K- Nearest Neighbor (KNN), and support vector machine (SVM), and the results shows that the combination of DT and AdaBoost classifiers outperforms others. The ontology matching has been deemed as a binary classification problem (Mao, Peng & Spring, 2011), which utilized SVM to solve it by using non-instance learning-based ontology alignment method. In recent years, several neural network (NN) based OMTs were proposed. Khoudja, Fareh & Bouarfa (2018a) proposed a matching approach to integrate several state-of-the-art ontology matchers by using NN for the improvement of the alignment quality. Bento, Zouaq & Gagnon (2020) used convolutional neural network (CNN) to align diverse ontologies, which shows good performance on different domains. Jiang & Xue (2020) presented a long short-term memory networks (LSTM)-based OMT to align biomedical ontologies by using the structural and semantic information of concepts. Iyer, Agarwal & Kumar (2020) proposed an NN-based OMT, VeeAlign, which utilized dual attention to calculate the contextualized representation of concepts and showed excellent performance over state-of-the-art OMTs. However, most ML-based OMTs need the usage of reference alignment, which is unavailable on actual matching tasks. To overcome this disadvantage, the SNN-OM is proposed, which enhances the performance without using the reference alignment.

## Preliminary

### Sensor ontology and sensor ontology matching

Definition 1 An ontology is defined as a quadruple (Xue & Wang, 2015)

**Figure 1:**
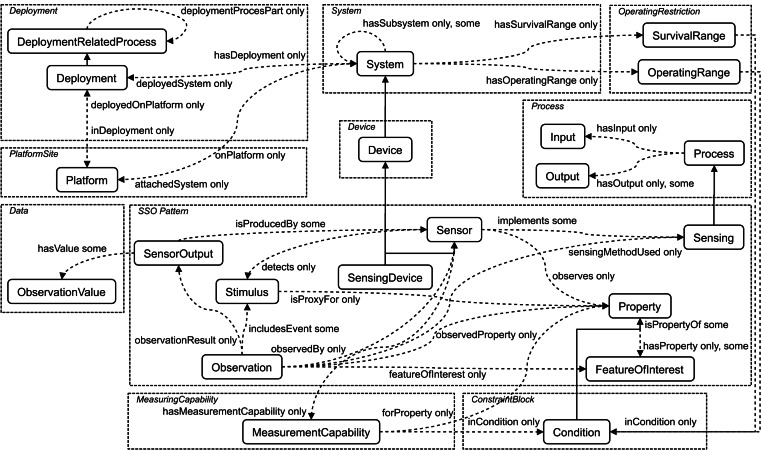
The core classes and properties of SSN ontology.

(1) \begin{eqnarray*}O=(IN,OP,DP,CL)\end{eqnarray*}

where *IN* is a quantity of individuals; *OP* is a quantity of object-type properties; *DP* is a quantity of data-type properties; *CL* is a quantity of classes. In particular, *IN*, *OP*, *DP*, and *CL* are called concept. Figure 1 shows the core classes and properties of SSN ontology (https://www.w3.org/2005/Incubator/ssn/ssnx/ssn) partitioned by conceptual modules. The rounded rectangle denotes the class, e.g., “Sensor” and “Stimulus”, and the dotted arrow the property, e.g., “hasValue”, “hasOutput”, and “isPropertyOf”. The solid one-way arrow means that two concepts are the parent–child relationship.

**Figure 2:**
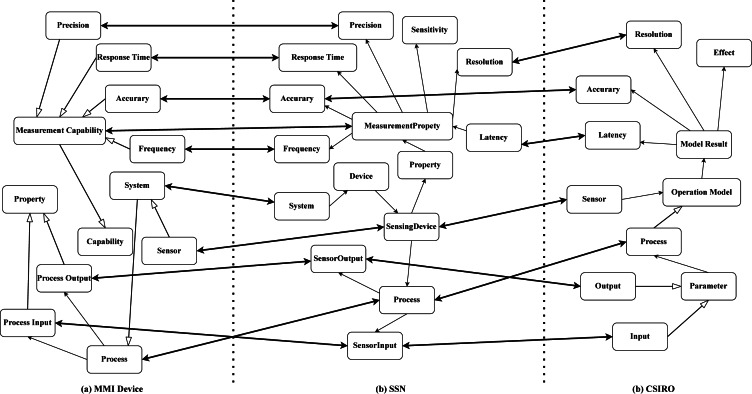
An example of sensor ontology alignment.

Definition 2 An ontology alignment is a set of correspondences, and a correspondence is defined as follows (Jiang & Xue, 2021):

(2) \begin{eqnarray*}Cor=({c}^{{^{\prime}}},c,s,t)\end{eqnarray*}

where *c*′ and *c* are the concept from two ontologies to be aligned; *s* is the similarity score; and and *t* is the relation type of *c*′ and *c*. Figure 2 depicts three sensor ontologies and their alignment that the ontologies are Marine Metadata Interoperability (MMI) device ontology ( https://mmisw.org/ont/mmi/device), Commonwealth Scientific and Industrial Research Organisation (CSIRO) sensor ontology (Neuhaus & Compton, 2009), and SSN ontology (Compton et al., 2012). The hollow one-way arrow means that the intermediate concepts between the two concepts are omitted and the solid one-way arrow means that two concepts are the parent–child relationship. The double-sided arrow links two concepts forming a correspondence, e.g., “SensingDevice” in SSN ontology and “Sensor” in CSIRO ontology are connected building a correspondence. Furthermore, all correspondences constitute an alignment, and reference alignment is the golden alignment provided by the domain experts, which is employed to evaluate OMTs’ performance.

Definition 3 The process of ontology matching is a function (Xue, Chen & Yao, 2018; Xue & Zhang, 2021):

(3) \begin{eqnarray*}A=\phi ({O}_{1},{O}_{2},RA,R,P)\end{eqnarray*}

where *A* is the final alignment; *O*_1_ and *O*_2_ are two to-be-matched ontologies; *RA* is the reference alignment (optional); *R* is the utilized resources; and *P* is the utilized parameters.

### Alignment’s evaluation metrics

Generally, precision (*P*), recall (*R*), and f-measure (*F*) are utilized to test the matching results’ quality (Xue, 2020; Xue et al., 2021b; Xue et al., 2021a):

(4) \begin{eqnarray*}R= \frac{correct\text{_}found\text{_}correspondences}{all\text{_}possible\text{_}correspondences} \end{eqnarray*}

(5) \begin{eqnarray*}P= \frac{correct\text{_}found\text{_}correspondences}{all\text{_}found\text{_}correspondences} \end{eqnarray*}

(6) \begin{eqnarray*}F= \frac{2\times R\times P}{R+P} \end{eqnarray*}

where *P* and *R* respectively indicate the accuracy and completeness of the results. *P* equals 1 denoting all found correspondences are correct, while *R* equals 1 representing that all correct correspondences are found; *F* is the harmonic mean of *P* and *R* to balance them.

### Concept similarity measure

CSM is the kernel technique in the ontology matching domain, which outputs a real number in [0,1] by considering the two input concepts’ information (Xue & Chen, 2019). Generally, CSM can be divided into three categories: string-based CSM, linguistics-based CSM, and structure-based CSM. Furthermore, string-based CSM computes the edit distance by using entities’ identifier, label or comment, linguistics-based CSM outputs the similar value through the external dictionaries or corpora, e.g., WordNet, and structure-based CSM outputs two concepts’ similarity value by taking their adjacent concepts into consideration.

In this work, the CSM is adopted to determine the anchor correspondences from two to-be-matched ontologies, which gets rid of the limitation that most NN-based OMTs rely on reference alignment. The anchor correspondences are utilized to build the training data for the model’s training. In particular, for the effectiveness and efficiency of matching process, the hybrid CSM is used that combines the n-gram similarity (string-based CSM), and the WordNet-based similarity (linguistics-based CSM), since they are greatly effective CSMs in the ontology matching field. The structure-based CSM often owns high time complexity, hence it is not advisable to employ it for building the training data, which could increase the time. Given two tokens *t*_1_ and *t*_2_, n-gram similarity and WordNet-based similarity are respectively defined as follows:

(7) \begin{eqnarray*}n-gram({t}_{1},{t}_{2})= \frac{{|}f({t}_{1},n)\cap f({t}_{2},n){|}}{min({|}{t}_{1}{|},{|}{t}_{2}{|})-n+1} \end{eqnarray*}

(8) \begin{eqnarray*}WordSim({t}_{1},{t}_{2})=\max _{{w}_{1}\in sen({t}_{1}),{w}_{2}\in sen({t}_{2})}[sim({w}_{1},{w}_{2})]\end{eqnarray*}

where |*t*_1_| and |*t*_2_| are respectively the length of *t*_1_ and *t*_2_, *f*(*t*_1_, *n*) represents a set of substrings of *t*_*i*_ with length *n*.*sen*(*t*_*i*_) denotes a set of possible meanings of the token *t*_*i*_. Here, *n* is empirically set as 3.

For the quality of used training data, given two concepts *c*′ and *c*, theirs’ labels and comments are lowercased, replaced underlines with spaces, and stripped of stop-words, respectively. Finally, four token sets ${T}_{label}^{{^{\prime}}}$, ${T}_{comment}^{{^{\prime}}}$, *T*_*label*_, and *T*_*comment*_ are generated for the similarity computing. As the diverse heterogeneity situation of the testing cases that some of the labels or comments are garbled or missed, so the designed CSM is as follows:

(9) \begin{eqnarray*}sim({c}^{{^{\prime}}},c)=max(si{m}_{label}({c}^{{^{\prime}}},c),si{m}_{comment}({c}^{{^{\prime}}},c))\end{eqnarray*}

where *sim*_*label*_(*c*′, *c*) and *sim*_*comment*_(*c*′, *c*) are respectively the label-based and comment-based similarity value by using concepts’ labels, and comments. To be specific, given two label token sets ${T}_{label}^{{^{\prime}}}$ and *T*_*label*_, represented by *T*_1_ and *T*_2_, then label-based CSM can be computed:

(10) \begin{eqnarray*}si{m}_{label}({c}^{{^{\prime}}},c)= \frac{\sum _{i=1}^{{|}{T}_{1}{|}}max{\{n-gram({T}_{1,i},{T}_{2,j}),WordSim({T}_{1,i},{T}_{2,j})\}}_{j=1}^{{|}{T}_{2}{|}}}{{T}_{1}} \end{eqnarray*}

where *T*_1,*i*_ and *T*_2,*j*_ respectively represent the |*T*_1_|’s *i*th token and |*T*_2_|’s *j*th token. The comment-based CSM is also calculated in this way only the input token sets is different.

## Siamese Neural Network based Ontology Matching technique

**Figure 3:**
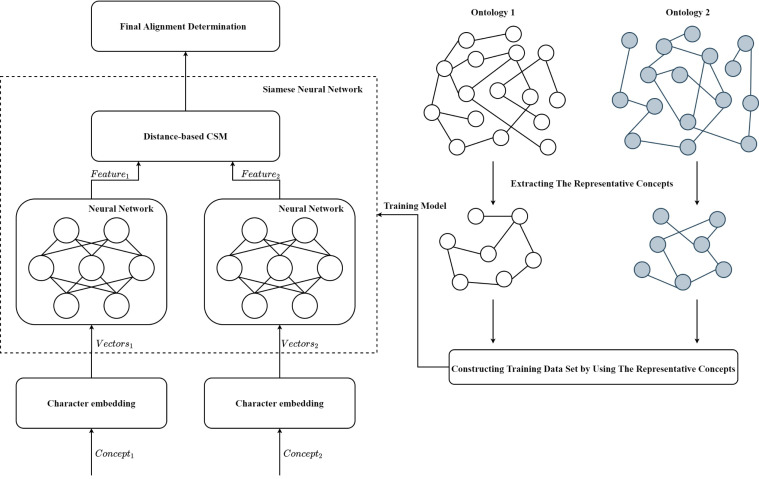
The framework of the siamese neural network based ontology matching technique.

The framework of SNN-OM is shown in Fig. 3. First, the representative concepts are extracted from two input ontologies. Then, the anchor correspondences are determined by using the CSM. After that, the training data set (positive and negative samples) are constructed to train the SNN model, which does not require the utilization of reference alignment. With the trained SNN, we can determine sensor concepts’ similarity by considering their semantic information. Finally, the alignment refinement method is utilized to further improve the alignment quality.

### The determination of representative concepts

**Figure 4:**
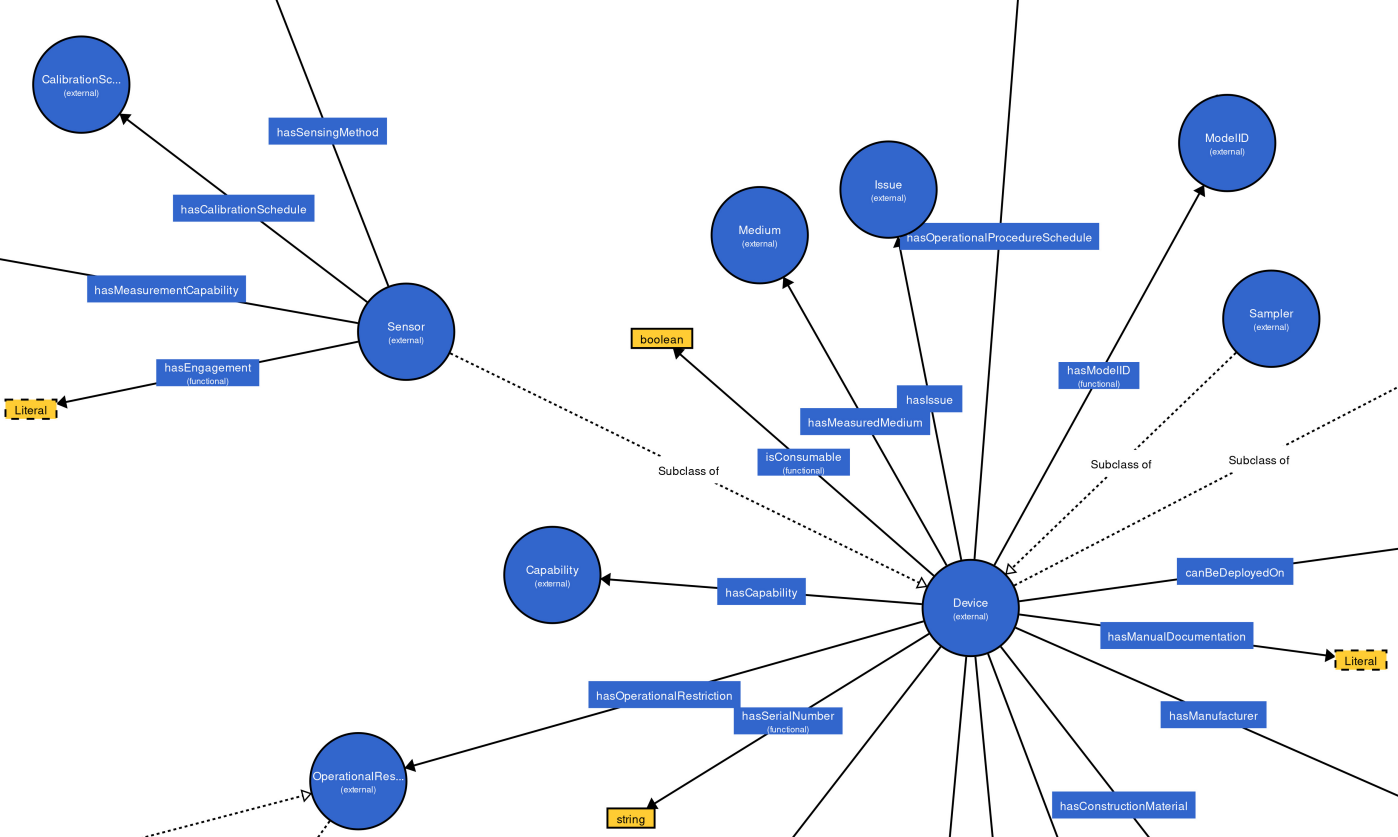
An example of MMI ontology’s class hierarchy graph.

The Degree Centrality (DC) is a measure to compute the significance of nodes in a graph, which is determined by the quantity of connections of the nodes (Bonacich, 2007; Bródka et al., 2011; Algergawy et al., 2015). A node with high DC is more significant than others (Algergawy et al., 2015; Pouriyeh et al., 2018). In order to build effective training data set and improve the matching efficiency, we extract representative concepts (nodes with the high numbers out-degree and int-degree in the class hierarchy graph) from the ontologies to be aligned. All concepts are first sorted using the DC and concepts with high rank will be selected to build the training data set. Empirically, we select the top 30% concepts, which is able to enhance the alignment’s quality and the matching efficiency. It can be seen in Fig. 4, the concepts “Device” and “Sensor”, whose DC are high, are more important than other concepts.

### Training data set construction

In this work, the positive samples are build by determining anchor correspondences through the proposed CSM rather than using the reference alignment. The proposed CSM (see Eqs. (9) and (10)) is used to calculate the similarity value among representative concepts. The correspondence with the similarity score higher than the threshold 0.95 is regarded as candidate anchor correspondence and their set is denoted as ***C***_*c*_. However, the threshold based filtering strategy is not able to guarantee that all anchors are correct correspondences. Therefore, a logic reasoning based filtering method (see also ‘Alignment refinement’) is employed on the candidate anchors ***C***_*c*_ to enhance the result’s confidence, then, the final anchor correspondence set ***C***_*f*_ is obtained. The correspondence in ***C***_*f*_ is the positive sample and the correspondence in ***C***_*c*_∖***C***_*f*_ is the negative sample. To balance the dataset to prevent biasing the training process, it is necessary to make the same number of positive and negative samples. Given an anchor correspondence *Cor* = (*c*′, *c*, *s*, *t*) ∈ ***C***_*f*_ that *c*′ from ontologies *O*_1_, the negative sample is produced by replacing *c*′ with a concept *c*^′′^ randomly selected from *O*_1_. Finally, the built data set is employed to train the SNN model.

### Matching ontologies through siamese neural network

**Figure 5:**
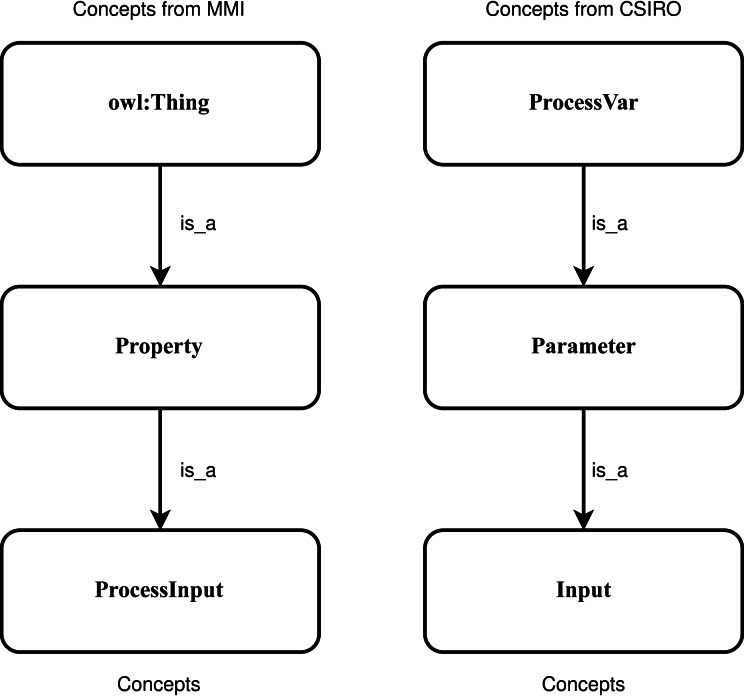
An example of the positive sample considering the context information of the concepts.

SNN is a type of NN that inputs two different vectors into two same structure and weights networks and it is used to find the similarity of the inputs by comparing its feature vectors. It can learn semantic similarity as it focuses on learning embedding, bringing the same concepts closely together (Melekhov, Kannala & Rahtu, 2016; Mueller & Thyagarajan, 2016). According to previous research (Bento, Zouaq & Gagnon, 2020; Iyer, Agarwal & Kumar, 2020; Jiang & Xue, 2020), the ancestry nodes are more suitable for ontology matching rather than other nodes. To capture the sensor concepts’ context information, two concepts and their two super-classes, respectively denoted by *Concept*_1_ and *Concept*_2_, are used as two the input. Figure 5 shows an example of the positive sample that semantically consistent concepts “ProcessInput” and “Input” and their two sup-classes are utilized to input. To feed the SNN the numeric representation vectors, the character embeddings (https://github.com/minimaxir/char-embeddings) is utilized whose possible character is a representation vector in 300 dimensions, and the value in each dimension is normalized in the interval [0,1]. In this work, the used structure of networks is an Attention-based Bidirectional Long Short-Term Memory Network (Zhou et al., 2016), which is able to connect future and past contexts of sensor concept pairs while catch the significant part to enhance the model’s performance, and capture the semantic relationships and features of input concept pairs.

Input two context vectors *Concept*_1_ and *Concept*_2_ of *c*′ and *c* to the trained SNN, the similarity value is calculated by the distance-based CSM, which is defined as follows:

(11) \begin{eqnarray*}si{m}_{distance}({c}^{{^{\prime}}},c)=1- \frac{{|}{|}Featur{e}_{1}-Featur{e}_{2}{|}{|}}{{|}{|}Featur{e}_{1}{|}{|}+{|}{|}Featur{e}_{2}{|}{|}} \end{eqnarray*}

where ||⋅|| denotes the Euclidean norm, *Feature*_1_ and *Feature*_2_ are the output feature vectors, $ \frac{{|}{|}Featur{e}_{1}-Featur{e}_{2}{|}{|}}{{|}{|}Featur{e}_{1}{|}{|}+{|}{|}Featur{e}_{2}{|}{|}} $ is the normalized distance, denoted as *d*. The similarity value can be obtained according to the principle that the smaller the distance, the greater the similarity score. In addition, an effective optimization algorithm, Adam optimizer (Kingma & Ba, 2014; Yi, Ahn & Ji, 2020; Ruder, 2016), and the most commonly used contrastive loss (Koch, Zemel & Salakhutdinov, 2015) for SNN are employed to optimize the weights of networks:

(12) \begin{eqnarray*}{L}_{contrastive}=\sum _{i=0}^{N} \frac{{y}_{i}\times {d}_{i}+(1-{y}_{i})\times max\{m-{d}_{i},0\}}{2N} \end{eqnarray*}

where *N* is the number of samples; *d* is the distance of two output feature vectors; *y*_*i*_ is the sample type that *y*_*i*_ equals 0 denoting the negative sample and 1 representing the positive sample; *m* is a margin value.

**Figure 6:**
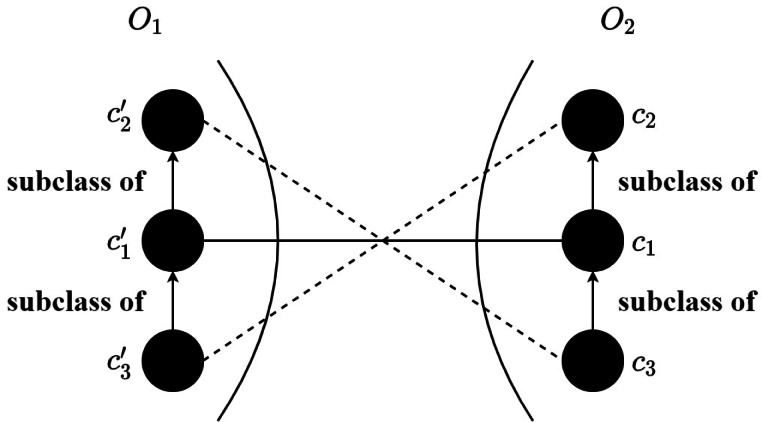
An example of inconsistent correspondences.

### Alignment refinement

By means of the trained SNN, the *M* × *N* similarity matrix can be obtained that *M* and *N* are the numbers of input concepts of *O*_1_ and *O*_2_, respectively. In particular, the concepts corresponding to the anchor are not inputted into the model for calculation, which decreases the matching time. Each element in the similarity matrix is the similarity score calculated through the (11). After that, an alignment refining method is proposed to enhance the quality of the alignment, which makes use of the sensor ontology’s concept hierarchy to remove the logically conflict correspondences. The alignment refinement method is shown in ?? and the detailed process is as follows: (1) the correspondences are sorted by descending, (2) to ensure the precision of the final alignment, a threshold is adopted to filter the correspondences (3) the correspondence with the greatest similarity is selected, (4) finally, the rest correspondences are chosen one by one if it does not conflict with previous correspondences. To be specific, in Fig. 6, two correspondences (${c}_{1}^{{^{\prime}}},{c}_{1}$) and (${c}_{2}^{{^{\prime}}},{c}_{3}$) conflict that ${c}_{1}^{{^{\prime}}}$ is the subclass of ${c}_{2}^{{^{\prime}}}$ and *c*_3_ is the subclass of *c*_1_, then the correspondence with the lower similarity score will be discarded. Finally, the anchor correspondences and the above correspondences without filtration form the final alignment.


 
_______________________ 
Algorithm 1 Alignment Refinement_______________________________________________________ 
  1:  Input:   The  similarity  matrix  with  row=  M,  column  =N;  the  corre- 
     spondences set Cs  =  {Cor1,Cor2,...,CorM×N},  Cork  =  (c′i,cj,s),  k ∈ 
  {1,2,...,M × N}, i ∈{1,2,...,M}, j ∈{1,2,...,N}; 
  2:  Output: the final alignment; 
  3:  Sort correspondences in descending order according to similarity score, then 
     the sorted correspondences set C′s = {Cor′1,Cor′2,...,Cor′M×N}; 
  4:  A threshold is adopted on C′s to remove a number of correspondences; 
  5:  for h = 1 : C′s.Length do 
 6:     if Cor′h then 
 7:         if h == 1 then 
 8:            Reserve the correspondence Cor′h = (c′i,cj,s); 
  9:            Remove the correspondences in C′s if its row or column is same with 
              the correspondence Cor′h = (c′i,cj,s); 
10:         else 
11:            if Correspondence Cor′h does not conflict with previous correspon- 
              dences then 
12:                Reserve the correspondence Cor′h = (c′i,cj,s); 
13:                Remove the correspondences in C′s if its row or column is same 
                 with the correspondence Cor′h = (c′i,cj,s); 
14:            else 
15:                Remove the correspondence Cor′h = (c′i,cj,s) in C′s; 
16:            end if 
17:         end if 
18:     end if 
19:  end for____________________________________________________________________________    


## Experiment

In the conducted experiment, the benchmark of Ontology Alignment Evaluation Initiative (OAEI) (http://oaei.ontologymatching.org/2016/benchmarks/index.html) and three pairs of sensor ontologies are utilized to test our approach’s performance. The succinct statement of OAEI’s benchmark and three sensor ontologies, i.e., MMI (http://marinemetadata.org/community/teams/cog), CSIRO (Neuhaus & Compton, 2009), and SSN (Compton et al., 2012), are shown in Tables 1 and 2. OAEI’s OMTs and four state-of-the-art sensor OMTs, i.e., CODI (Noessner et al., 2010), ASMOV (Jean-Mary, Shironoshita & Kabuka, 2009), SOBOM (Xu et al., 2010), and FuzzyAlign (Fernandez et al., 2013), are compared with the proposed approach.

**Figure 7:**
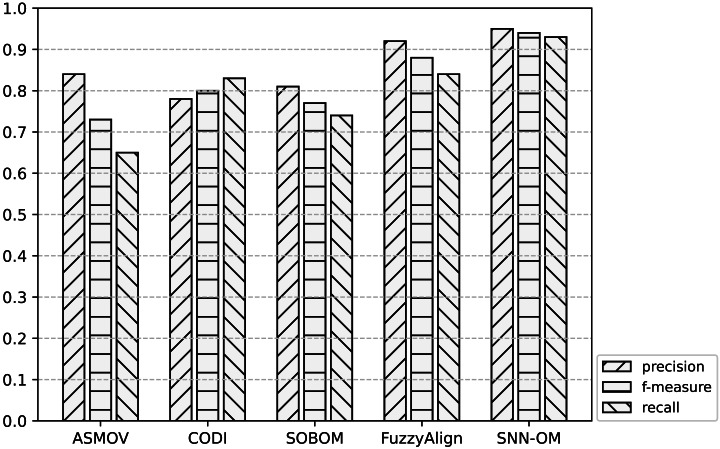
Comparison of SNN-OM and the state-of-the-art sensor OMTs on MMI-SSN matching task.

**Figure 8:**
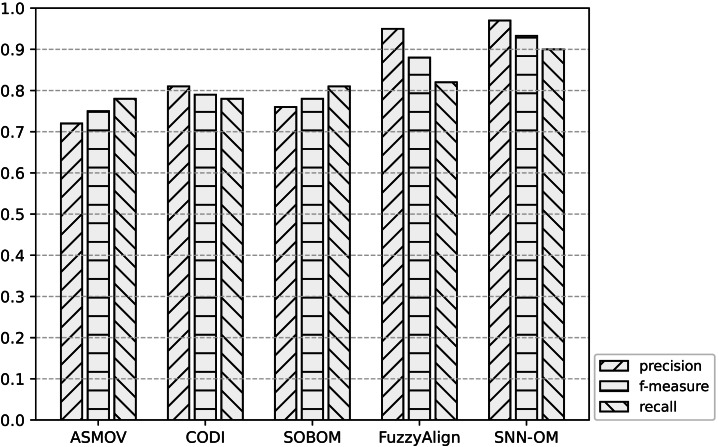
Comparison of SNN-OM and the state-of-the-art sensor OMTs on CSIRO-SSN matching task.

**Figure 9:**
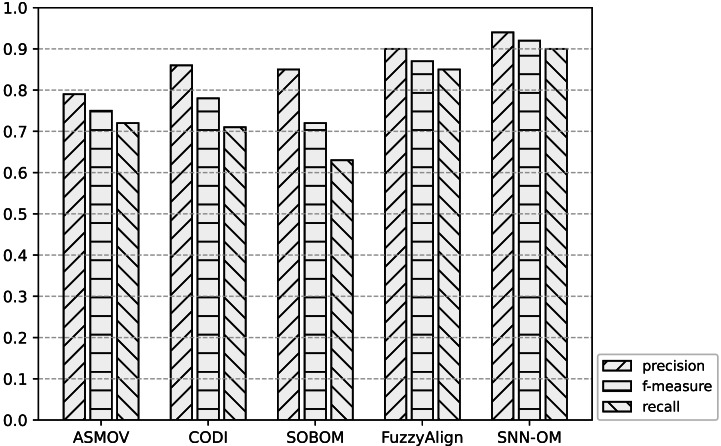
Comparison of SNN-OM and the state-of-the-art sensor OMTs on MMI-CSIRO matching task.

Tables 3–5 show the comparison among SNN-OM and OAEI’s OMTs on Benchmark in terms of precision, recall, and f-measure respectively. Table 6 compares SNN-OM with the state-of-the-art OMTs in terms of the matching efficiency. Figures 7–9 present the performance comparison of SNN-OM and the state-of-the-art sensor OMTs on sensor OM tasks.

**Table 1:** The succinct statement on benchmark testing cases.

ID	Succinct Statement
1XX	Two identical ontologies.
2XX	Two ontologies with different lexical, linguistic or structural characters.
3XX	The real ontologies.

**Table 2:** The succinct statement on sensor ontologies.

Ontology	Succinct Statement
CSIRO	Describing sensors and deployments.
MMI Device	Describing oceanographic devices, sensors and samplers.
SSN	Describing sensors, deployments, observations, measurement processes, and capabilities.

**Table 3:** Comparison of SNN-OM and OAEI’s OMTs in terms of precision on benchmark.

Test	edna	AgrMaker	AROMA	ASMOV	CODI	Ef2Match	Falcon	GeRMeSMB	MapPSO	SOBOM	TaxoMap	SNN-OM
101	1.00	0.98	1.00	1.00	1.00	1.00	1.00	1.00	1.00	1.00	1.00	1.00
103	1.00	0.98	1.00	1.00	1.00	1.00	1.00	1.00	1.00	1.00	1.00	1.00
104	1.00	0.98	1.00	1.00	1.00	1.00	1.00	1.00	1.00	1.00	1.00	1.00
201	0.04	0.98	1.00	1.00	0.88	1.00	0.97	0.95	0.47	1.00	1.00	0.97
203	1.00	0.96	1.00	1.00	1.00	1.00	1.00	0.99	1.00	1.00	0.97	1.00
204	0.93	0.98	1.00	1.00	0.79	1.00	0.96	0.99	0.98	1.00	1.00	1.00
205	0.34	0.96	1.00	1.00	0.68	0.95	0.97	1.00	0.82	1.00	1.00	0.98
206	0.54	0.98	1.00	1.00	0.81	1.00	0.94	0.94	0.90	1.00	1.00	1.00
221	1.00	0.95	1.00	1.00	1.00	1.00	1.00	1.00	1.00	1.00	1.00	1.00
222	0.96	0.97	1.00	1.00	1.00	1.00	1.00	1.00	1.00	1.00	0.88	1.00
223	1.00	0.92	0.95	1.00	1.00	1.00	1.00	0.99	0.98	0.99	0.88	1.00
224	1.00	0.98	1.00	1.00	1.00	1.00	1.00	1.00	1.00	1.00	1.00	1.00
225	1.00	0.98	1.00	1.00	1.00	1.00	1.00	1.00	1.00	1.00	1.00	1.00
228	1.00	1.00	1.00	1.00	1.00	1.00	1.00	1.00	1.00	1.00	1.00	1.00
230	0.74	0.84	0.93	0.95	1.00	0.94	0.94	1.00	0.97	0.94	0.83	1.00
231	1.00	0.98	1.00	1.00	1.00	1.00	1.00	1.00	1.00	1.00	1.00	1.00
232	1.00	0.95	1.00	1.00	1.00	1.00	1.00	1.00	1.00	1.00	1.00	1.00
233	1.00	1.00	1.00	1.00	1.00	1.00	1.00	1.00	1.00	1.00	1.00	1.00
236	1.00	1.00	1.00	1.00	1.00	1.00	1.00	1.00	1.00	1.00	1.00	1.00
237	0.96	0.97	1.00	1.00	1.00	1.00	1.00	1.00	0.99	1.00	0.88	1.00
238	1.00	0.92	0.94	1.00	0.99	1.00	1.00	0.98	0.97	0.98	0.88	1.00
239	0.33	0.97	0.97	0.97	0.97	0.97	1.00	0.97	0.97	0.97	0.88	0.97
240	0.38	0.89	0.82	0.97	0.94	0.97	1.00	0.85	0.91	0.97	0.88	1.00
241	1.00	1.00	1.00	1.00	1.00	1.00	1.00	1.00	1.00	1.00	1.00	1.00
246	0.33	0.97	0.97	0.97	0.97	0.97	1.00	0.97	0.97	0.93	0.88	0.97
250	0.01	1.00	0.00	1.00	0.00	1.00	0.00	1.00	0.04	0.67	0.00	1.00
257	0.00	1.00	0.00	1.00	0.00	1.00	0.00	1.00	0.08	0.83	0.00	1.00
258	0.04	0.98	1.00	0.97	1.00	1.00	0.00	0.51	0.08	0.94	1.00	1.00
259	0.02	0.92	1.00	0.96	1.00	1.00	0.00	0.60	0.05	0.89	1.00	1.00
260	0.00	0.93	0.00	0.92	0.00	0.67	0.00	0.00	0.14	0.33	0.00	0.93
261	0.00	0.87	0.00	0.88	0.00	0.67	0.00	0.50	0.04	0.22	0.00	0.91
301	0.47	1.00	0.86	0.91	0.93	0.92	0.91	0.92	0.68	0.90	0.69	0.91
302	0.32	1.00	0.73	0.86	1.00	0.93	0.90	0.81	0.50	0.86	0.76	0.95
303	0.00	0.83	0.69	0.78	0.92	0.85	0.77	0.00	0.00	0.46	0.48	0.93
304	0.74	0.86	0.91	0.94	0.94	0.95	0.96	0.91	0.76	0.91	0.90	0.97
mean	0.63	0.95	0.85	0.97	0.85	0.96	0.80	0.88	0.75	0.90	0.82	0.98

**Table 4:** Comparison of SNN-OM and OAEI’s OMTs in terms of recall on benchmark.

Test	edna	AgrMaker	AROMA	ASMOV	CODI	Ef2Match	Falcon	GeRMeSMB	MapPSO	SOBOM	TaxoMap	SNN-OM
101	1.00	1.00	0.97	1.00	1.00	1.00	1.00	1.00	1.00	1.00	0.34	1.00
103	1.00	1.00	0.98	1.00	1.00	1.00	1.00	1.00	1.00	1.00	0.34	1.00
104	1.00	1.00	0.98	1.00	0.98	1.00	1.00	1.00	1.00	1.00	0.34	1.00
201	0.04	0.86	0.90	1.00	0.07	0.62	0.97	0.94	0.38	0.91	0.34	0.97
203	1.00	1.00	0.66	1.00	0.76	1.00	1.00	0.97	1.00	1.00	0.33	1.00
204	0.93	0.97	0.95	1.00	0.69	0.98	0.96	0.98	0.98	0.99	0.34	0.99
205	0.34	0.88	0.90	0.99	0.18	0.75	0.97	0.98	0.66	0.93	0.34	0.98
206	0.54	0.88	0.91	0.99	0.26	0.77	0.94	0.90	0.80	0.93	0.34	0.93
221	1.00	1.00	0.98	1.00	0.96	1.00	1.00	1.00	1.00	1.00	0.34	1.00
222	1.00	1.00	0.98	1.00	1.00	1.00	1.00	0.99	1.00	1.00	0.31	1.00
223	1.00	0.98	0.92	1.00	1.00	1.00	1.00	0.94	0.98	0.99	0.30	1.00
224	1.00	1.00	0.95	1.00	1.00	1.00	0.99	1.00	1.00	1.00	0.34	1.00
225	1.00	1.00	0.98	1.00	0.99	1.00	1.00	1.00	1.00	1.00	0.34	1.00
228	1.00	1.00	1.00	1.00	1.00	1.00	1.00	1.00	1.00	1.00	1.00	1.00
230	1.00	0.97	0.93	1.00	0.97	1.00	1.00	0.89	1.00	1.00	0.35	1.00
231	1.00	1.00	0.97	1.00	1.00	1.00	1.00	1.00	1.00	1.00	0.34	1.00
232	1.00	1.00	0.94	1.00	0.94	1.00	0.99	1.00	1.00	1.00	0.34	1.00
233	1.00	1.00	1.00	1.00	0.88	1.00	1.00	0.97	1.00	1.00	1.00	1.00
236	1.00	1.00	1.00	1.00	1.00	1.00	1.00	1.00	1.00	1.00	1.00	1.00
237	1.00	1.00	0.94	1.00	0.99	1.00	0.99	1.00	1.00	1.00	0.31	1.00
238	1.00	0.96	0.91	1.00	0.99	1.00	0.99	0.95	0.97	0.98	0.3	1.00
239	1.00	1.00	1.00	1.00	1.00	1.00	1.00	1.00	1.00	1.00	1.00	1.00
240	1.00	0.94	0.85	1.00	0.97	1.00	1.00	0.85	0.94	1.00	0.88	1.00
241	1.00	1.00	0.97	1.00	0.88	1.00	1.00	0.97	1.00	1.00	1.00	1.00
246	1.00	1.00	0.97	1.00	1.00	1.00	1.00	1.00	1.00	0.97	1.00	1.00
250	0.03	0.39	0.00	0.45	0.00	0.06	0.00	0.03	0.03	0.12	0.00	1.00
257	0.00	0.39	0.00	0.33	0.00	0.06	0.00	0.03	0.06	0.15	0.00	1.00
258	0.04	0.65	0.01	0.78	0.01	0.04	0.00	0.19	0.06	0.31	0.01	0.83
259	0.02	0.67	0.01	0.78	0.01	0.04	0.00	0.19	0.04	0.35	0.01	0.80
260	0.00	0.45	0.00	0.41	0.00	0.07	0.00	0.00	0.10	0.03	0.00	0.83
261	0.00	0.39	0.00	0.42	0.00	0.06	0.00	0.03	0.03	0.06	0.00	0.88
301	0.78	0.42	0.64	0.81	0.24	0.58	0.68	0.58	0.61	0.78	0.31	0.82
302	0.65	0.19	0.23	0.63	0.42	0.58	0.58	0.27	0.02	0.65	0.27	0.62
303	0.00	0.73	0.52	0.88	0.50	0.81	0.77	0.00	0.00	0.54	0.29	0.82
304	0.95	0.86	0.78	0.97	0.61	0.95	0.93	0.66	0.68	0.92	0.37	0.91
mean	0.72	0.84	0.73	0.89	0.66	0.78	0.79	0.75	0.72	0.81	0.40	0.95

**Table 5:** Comparison of SNN-OM and OAEI’s OMTs in terms of f-measure on benchmark.

Test	edna	AgrMaker	AROMA	ASMOV	CODI	Ef2Match	Falcon	GeRMeSMB	MapPSO	SOBOM	TaxoMap	SNN-OM
101	1.00	0.99	0.98	1.00	1.00	1.00	1.00	1.00	1.00	1.00	0.50	1.00
103	1.00	0.99	0.99	1.00	1.00	1.00	1.00	1.00	1.00	1.00	0.50	1.00
104	1.00	0.99	0.99	1.00	0.99	1.00	1.00	1.00	1.00	1.00	0.50	1.00
201	0.04	0.91	0.94	1.00	0.12	0.76	0.97	0.94	0.42	0.95	0.50	0.97
203	1.00	0.98	0.79	1.00	0.86	1.00	1.00	0.97	1.00	1.00	0.49	1.00
204	0.93	0.97	0.97	1.00	0.73	0.98	0.96	0.98	0.98	0.99	0.50	0.99
205	0.34	0.91	0.94	0.99	0.28	0.83	0.97	0.98	0.73	0.96	0.50	0.98
206	0.54	0.92	0.95	0.99	0.39	0.87	0.94	0.91	0.84	0.96	0.50	0.96
221	1.00	0.97	0.99	1.00	0.98	1.00	1.00	1.00	1.00	1.00	0.50	1.00
222	0.98	0.98	0.99	1.00	1.00	1.00	1.00	0.99	1.00	1.00	0.45	1.00
223	1.00	0.94	0.93	1.00	1.00	1.00	1.00	0.96	0.98	0.99	0.44	1.00
224	1.00	0.98	0.97	1.00	1.00	1.00	0.99	1.00	1.00	1.00	0.50	1.00
225	1.00	0.98	0.98	1.00	0.99	1.00	1.00	1.00	1.00	1.00	0.50	1.00
228	1.00	1.00	1.00	1.00	1.00	1.00	1.00	1.00	1.00	1.00	1.00	1.00
230	0.85	0.90	0.93	0.97	0.98	0.96	0.96	0.94	0.98	0.96	0.49	1.00
231	1.00	0.98	0.98	1.00	1.00	1.00	1.00	1.00	1.00	1.00	0.50	1.00
232	1.00	0.97	0.96	1.00	0.96	1.00	0.99	1.00	1.00	1.00	0.50	1.00
233	1.00	1.00	1.00	1.00	0.93	1.00	1.00	0.98	1.00	1.00	1.00	1.00
236	1.00	1.00	1.00	1.00	1.00	1.00	1.00	1.00	1.00	1.00	1.00	1.00
237	0.98	0.98	0.96	1.00	0.99	1.00	0.99	1.00	0.99	1.00	0.45	1.00
238	1.00	0.93	0.92	1.00	0.99	1.00	0.99	0.96	0.97	0.98	0.44	1.00
239	0.49	0.98	0.98	0.98	0.98	0.98	1.00	0.98	0.98	0.98	0.93	0.98
240	0.55	0.91	0.83	0.98	0.95	0.98	1.00	0.85	0.92	0.98	0.88	1.00
241	1.00	1.00	0.98	1.00	0.93	1.00	1.00	0.98	1.00	1.00	1.00	1.00
246	0.49	0.98	0.97	0.98	0.98	0.98	1.00	0.98	0.98	0.94	0.93	0.98
250	0.01	0.56	0.00	0.62	0.00	0.11	0.00	0.05	0.03	0.20	0.00	1.00
257	0.00	0.56	0.00	0.49	0.00	0.11	0.00	0.05	0.06	0.25	0.00	1.00
258	0.04	0.78	0.01	0.86	0.01	0.07	0.00	0.27	0.06	0.46	0.01	0.91
259	0.02	0.77	0.01	0.86	0.01	0.07	0.00	0.28	0.04	0.50	0.01	0.89
260	0.00	0.60	0.00	0.56	0.00	0.12	0.00	0.00	0.11	0.05	0.00	0.88
261	0.00	0.53	0.00	0.56	0.00	0.11	0.00	0.05	0.03	0.09	0.00	0.89
301	0.58	0.59	0.73	0.85	0.38	0.71	0.77	0.71	0.64	0.83	0.42	0.86
302	0.42	0.31	0.34	0.72	0.59	0.71	0.70	0.40	0.03	0.74	0.39	0.75
303	0.00	0.77	0.59	0.82	0.64	0.82	0.77	0.00	0.00	0.49	0.36	0.87
304	0.83	0.86	0.84	0.95	0.73	0.95	0.94	0.76	0.71	0.91	0.52	0.94
mean	0.65	0.87	0.75	0.91	0.69	0.80	0.79	0.77	0.72	0.83	0.49	0.96

It can be seen in Table 3, the SNN-OM is able to obtain high precision in diverse heterogeneous situations, which performs much better than most OMTs denoting most of the found correspondences are correct. Table 4 demonstrates that the proposed matching technique is effective as well, which can be competent for a variety of heterogeneous problems indicating most of the correct correspondences are found. About f-measure, the superiority of our method is shown again. In particular, SNN-OM is capable of gaining high-quality alignment over other OMTs that the average values of precision, recall, and f-measure are the highest. As regards matching efficiency, Table 6 shows that, by using the representative concepts, SNN-OM owns the minimum value of runtime (i.e., 90s), and maximum values of f-measure per second (0.0103) and f-measure. Our similarity measure takes string, semantic, and context information of concepts into account. And hence the SNN-OM’s f-measure value is higher than other OAEI’s OMTs that only consider one or two types of CSM, such as XMap, Pheno family, LogMap family, and CroMatcher. In summary, SNN-OM has the capability of determining the superior correspondences and the experimental results illustrate the both effectiveness and efficiency of our method on the benchmark.

**Table 6:** Comparison on matching efficiency among SNN-OM and OAEI’s OMTs on benchmark.

OMT	f-measure	Runtime (second)	f-measure per second
XMap	0.56	123	0.0045
PhenoMP	0.01	1833	0.0000
PhenoMM	0.01	1743	0.0000
PhenoMF	0.01	1632	0.0000
LogMapBio	0.32	54439	0.0000
LogMapLt	0.46	96	0.0048
LogMap	0.55	194	0.0028
Lily	0.89	2211	0.0004
CroMatcher	0.89	1100	0.0008
AML	0.38	120	0.0031
SNN-OM	0.93	90	0.0103

It can be seen from Figs. 7–9 that SNN-OM is the most competent method to address the sensor data heterogeneous problem, whose precision, recall, and f-measure are better than four state-of-the-art sensor OMTs on three sensor matching tasks. The top 2 OMT is the FuzzyAlign, which outperforms the ASMOV, CODI, and SOBOM in terms of precision and recall as it takes semantic, linguistics, and structure information of concepts into account. However, FuzzyAlign adopted too many CSMs that bring about conflicting correspondences, which reduces the recall value. To sum up, by considering the concepts’ semantic and context information and introducing the logical reasoning method to promote the quality of correspondences, SNN-OM is able to handle diverse sensor ontology matching problems.

## Conclusion

To solve the heterogeneity problem of sensor data, it is necessary to carry out the sensor ontology matching process to determine the correspondences among different sensor concepts with the same semantic annotation. In this paper, the SNN-OM is proposed to align the sensor ontologies. Before the matching, to get rid of the limitation that most NN-based OMTs’ training requires the utilization of reference alignment, the representative concepts extraction method is used to build the effective training data set, which is able to enhance the model’s performance and reduce the time of training process. In addition, to determine the heterogeneous sensor concepts, a confidence calculation method is utilized by using the SNN, which takes sensor concepts’ semantic and context information into account to improve the sensor ontology alignment. After the matching, an alignment refining method is proposed to enhance the quality of the alignment, which makes use of the sensor ontology’s concept hierarchy to remove the logically conflict correspondences. The experimental results present that SNN-OM is capable of determining superior alignment which is better than the state-of-the-art OMTs.

In the future, we will focus on the improvement of the effectiveness and efficiency of SNN-OM. For the effectiveness, the training data set is vital, hence, the determination of anchor correspondences should be refined, and the output feature is crucial as well, therefore, we will be interested in capturing the semantic feature of concepts by using more concepts’ context information. In addition, alignment refinement method could be improved, e.g., using the constraints. For efficiency, we will be devoted to reducing the matching time by utilizing the evolutionary algorithm to determine the correspondences rather than the simple enumeration approach.

##  Supplemental Information

10.7717/peerj-cs.602/supp-1 Supplemental Information 1 The goal of this benchmark series is to identify the areas in which each alignment algorithm is strong and weak

10.7717/peerj-cs.602/supp-2 Supplemental Information 2 Source code

## Additional Information and Declarations

### Data Availability

The following information was supplied regarding data availability: The data and code are available in the Supplemental Files. The data also can be download from http://oaei.ontologymatching.org/tests/logs.html
