# Supplementary material for: Matching sensor ontologies through siamese neural networks without using reference alignment
Source: PeerJ Comput Sci. 2021 Jun 18;7:e602. doi: 10.7717/peerj-cs.602 (PMC8237319; doi:10.7717/peerj-cs.602)
Supplement: Supplemental Information 1 [file peerj-cs-07-602-s001.zip › 202/onto.html]

# 

Author: Nick Knouf <nknouf@mit.edu>  
Contributor: Antoine Zimmermann <antoine.zimmermann@inrialpes.fr>, Jérôme Euzenat,   
Date: 08/06/2005  
Version: $Id: onto.rdf,v 1.30 2008/05/27 14:41:13 euzenat Exp $

## Classes

**http://www.w3.org/1999/02/22-rdf-syntax-ns#List** (, *)*


**http://xmlns.com/foaf/0.1/Person** (, *)*


**http://xmlns.com/foaf/0.1/Organization** (, *)*


**sqdsq** (, *)*
:   - #sqndsqgy [0 1]
    - #dznbaln [0 1]
    - #sbqgzga [0 1]

    **zajdhqs** (, *)*
    :   - #dznbaln [1 1]
        - #zsbdgz [0 1]
        - #sddnsnqdb [0 1]
        - #dszbnz [0 1]
        - #sqndsqgy [1 1] *#dzshyuua*
        - #zand [1 1]
        - #sdsqddz [0 1]

        **dzajndsq** (, *)*
        :   - #iNi4b8XV *#dzqndbzq*

        **sqxsqkd** (, *)*
        :   - #iNi4b8XV *#dzqndbzq*
            - #TfniY *#dcsqdcsqd*

        **zdqssqdb** (, *)*
        :   - #PrSGUs *#deqdxcsqcsq*
            - #zadzjadns [0 1] *#zqedzbx*
            - #dzajj [0 1]
            - #sqdndvb [0 1]

    **qsdsnbsqd** (, *)*
    :   - #dznbaln [1 1]

        **zdzaudbd** (, *)*


        **scds** (, *)*


        **dcsqdsq** (, *)*
        :   - #sqdndvb [0 1]
            - #sdsqddz [0 1]
            - #dznbaln [1 1]

        **zeadza** (, *)*
        :   - #zand [1 1]
            - #dznbaln [1 1]
            - #zdsnsqdv [1 +oo]

    **sqdsopq** (, *)*
    :   - #dzayue [0 1]
        - #dznbaln [1 1]

        **hazdn** (, *)*
        :   - #zand [1 1]
            - #dzayue [1 1]
            - #sxqsnbvsq [1 1]
            - #sqndsqgy [1 1] *#dzshyuua*
            - #ndsbzh [0 1]
            - #zsbdgz [0 1]

        **dzqndbzq** (, *)*
        :   - #dsazdjz [0 1] [0 1]

        **vccfsq** (, *)*
        :   - #zand [1 1]
            - #dzayue [1 +oo]
            - #sznsbq [1 1]

        **dcsqdcsqd** (, *)*
        :   - #zand [1 1]
            - #sqnbyz [1 1]

        **deqdxcsqcsq** (, *)*
        :   - #zand [1 1]
            - #zassdzadb [1 1]

    **zdazsx** (, *)*
    :   - #zand [1 1]
        - #dznbaln [1 1]
        - #dzbn [1 1]
        - #sqndsqgy [1 1]

        **xsqlknk** (, *)*


        **zqdzqzai** (, *)*

    **wxsdxqsdh** (, *)*


    **dqzdxdcsqj** (, *)*
    :   - #zand [1 1]
        - #dznbaln [1 1]
        - #hsgiuyza [1 1]
        - #sqndsqgy [1 1] *#dzshyuua*
        - #ndsbzh [0 1]

        **sdcsqhyz** (, *)*


        **azzauhuie** (, *)*
        :   - #zandsbh [0 1]

    **dscdscg** (, *)*

**qsdsquj** (, *)*
:   - #dszabdza [1 1] *http://www.w3.org/2001/XMLSchema#string*
    - #dsza *http://www.w3.org/2001/XMLSchema#string*
    - #sdxsndxsqg *http://www.w3.org/2001/XMLSchema#string*
    - #sddnsnqdb [0 1]
    - #dszbnz [0 1]
    - #sqdnbs [0 1]
    - #YuEma *#hazdn*

**zqedzbx** (, *)*
:   - #dszabdza [1 1]
    - #sdnsbh *#izxnquo*
    - #dsza [0 1]
    - #sqnzkzn [0 1]
    - #zdjznzn [0 1]

**qzddj** (, *)*
:   - #zadszabnds [0 1] *http://www.w3.org/2001/XMLSchema#string*
    - #zdnzadh [0 1] *http://www.w3.org/2001/XMLSchema#string*
    - #zdzndh [0 1] *http://www.w3.org/2001/XMLSchema#string*

**izxnquo** (, *)*
:   super: *http://xmlns.com/foaf/0.1/Organization*  

    - #dszabdza [1 1]
    - #dsza [1 1]
    - #qzd [0 1]

    **zauio** (, *)*


    **zadazxn** (, *)*

**dsqdbz** (, *)*
:   super: *http://www.w3.org/1999/02/22-rdf-syntax-ns#List*  

    - http://www.w3.org/1999/02/22-rdf-syntax-ns#first [1 1] *http://xmlns.com/foaf/0.1/Person*
    - http://www.w3.org/1999/02/22-rdf-syntax-ns#rest [1 1] (*#dsqdbz* |  {

      <rdf:List@ttp://www.w3.org/1999/02/22-rdf-syntax-ns#nil>
      } )

**dnqsb** (, *)*
:   - #sqnhdbdj [1 1]
    - #mkalnshsq [1 1]

**dzshyuua** (, *)*
:   - #zdsnzsdh [1 1] *http://www.w3.org/2001/XMLSchema#gYear*
    - #asndzsd [0 1] *http://www.w3.org/2001/XMLSchema#gMonth*
    - #dznadzh [0 1] *http://www.w3.org/2001/XMLSchema#gDay*

## Properties

**http://www.w3.org/1999/02/22-rdf-syntax-ns#first**: http://www.w3.org/1999/02/22-rdf-syntax-ns#List -> \_ *()*


**http://www.w3.org/1999/02/22-rdf-syntax-ns#rest**: http://www.w3.org/1999/02/22-rdf-syntax-ns#List -> http://www.w3.org/1999/02/22-rdf-syntax-ns#List *()*


**iNi4b8XV**: #sqdsq -> #dzqndbzq *()*


**TfniY**: #sqdsq -> #sqdsopq *()*


**PrSGUs**: #zdqssqdb -> #deqdxcsqcsq *()*


**YuEma**: #qsdsquj -> #hazdn *()*


**qzd**: http://www.w3.org/2002/07/owl#Thing -> #qzddj *()*


**zadzjadns**: #zdqssqdb -> #zqedzbx *()*


**sdnsbh**: #zqedzbx -> http://xmlns.com/foaf/0.1/Organization *()*


**zandsbh**: #sqdsq -> http://www.w3.org/2002/07/owl#Thing *()*


**sbqgzga**: #sqdsq -> #dsqdbz *()*
:   **zand**: \_ -> \_ *()*


    **dzajj**: \_ -> \_ *()*


    **zdnzqnd**: #dscdscg -> \_ *()*

**hsgiuyza**: #dqzdxdcsqj -> #izxnquo *()*


**EzGNplyLBiS**: #sqdsopq -> \_ *()*
:   **sxqsnbvsq**: #hazdn -> #qsdsquj *()*


    **sznsbq**: #vccfsq -> #dzajndsq *()*


    **sqnbyz**: #dcsqdcsqd -> #sqxsqkd *()*


    **zassdzadb**: #deqdxcsqcsq -> #zdqssqdb *()*

**sqndsqgy**: (*#sqdsq* | *#zqedzbx*) -> #dzshyuua *()*


**sqdndvb**: (*#zdqssqdb* | *#dcsqdsq*) -> http://xmlns.com/foaf/0.1/Organization *()*


**sddnsnqdb**: (*#sqdsq* | *#qsdsquj*) -> #zauio *()*


**dzbn**: (*#zdazsx* | *#scds*) -> #zadazxn *()*


**zdjznzn**: (*#sqdsq* | *#zqedzbx*) -> #qzddj *()*


**dzayue**: #sqdsopq -> #dnqsb *()*

**http://purl.org/dc/elements/1.1/creator**\_ -> \_ *()*


**http://purl.org/dc/elements/1.1/contributor**\_ -> \_ *()*


**http://purl.org/dc/elements/1.1/description**\_ -> \_ *()*


**http://purl.org/dc/elements/1.1/date**\_ -> \_ *()*


**http://xmlns.com/foaf/0.1/firstName**\_ -> \_ *()*


**lastName**\_ -> \_ *()*


**http://xmlns.com/foaf/0.1/name**\_ -> \_ *()*


**zdzbh** #sqdsq -> http://www.w3.org/2001/XMLSchema#string *()*


**dsqndbsqx** #sqdsq -> http://www.w3.org/2001/XMLSchema#string *()*


**dsqnhza** #sqdsq -> http://www.w3.org/2001/XMLSchema#string *()*


**sdxsndxsqg** #qsdsquj -> http://www.w3.org/2001/XMLSchema#string *()*


**sqdnbs** #dzqndbzq -> http://www.w3.org/2001/XMLSchema#string *()*


**sdsqddz**(*#zajdhqs* | *#dcsqdsq*) -> http://www.w3.org/2001/XMLSchema#string *()*


**sdqz**(*#wxsdxqsdh* | *#zdzaudbd*) -> http://www.w3.org/2001/XMLSchema#string *()*


**zdsnsqdv** #sqdsq -> http://www.w3.org/2001/XMLSchema#string *()*


**dszbnz** #sqdsq -> http://www.w3.org/2001/XMLSchema#string *()*


**dznbaln** #sqdsq -> http://www.w3.org/2001/XMLSchema#string *()*


**sqbdzg**(*#dzqndbzq* | *#sdcsqhyz* | *#zdazsx*) -> http://www.w3.org/2001/XMLSchema#string *()*


**zadzqbsdg** #sqdsq -> http://www.w3.org/2001/XMLSchema#string *()*


**dsqndsz** #sqdsq -> http://www.w3.org/2001/XMLSchema#string *()*


**zdnzdzh** #sqdsq -> http://www.w3.org/2001/XMLSchema#string *()*


**zdzdzd** #sqdsq -> http://www.w3.org/2001/XMLSchema#string *()*


**szdnzak** #sqdsq -> http://www.w3.org/2001/XMLSchema#string *()*


**zadsznad** #sqdsq -> http://www.w3.org/2001/XMLSchema#string *()*


**zqdszh** #sqdsq -> http://www.w3.org/2001/XMLSchema#string *()*


**sqkxndc** #sqdsq -> http://www.w3.org/2001/XMLSchema#language *()*


**dzandzah** #sqdsq -> http://www.w3.org/2001/XMLSchema#string *()*


**dszbgz** #sqdsq -> http://www.w3.org/2001/XMLSchema#string *()*


**dsq** #sqdsq -> http://www.w3.org/2001/XMLSchema#string *()*


**dzsndzah** #sqdsq -> http://www.w3.org/2001/XMLSchema#string *()*


**zdsznbadzagh** #sqdsq -> http://www.w3.org/2001/XMLSchema#string *()*


**dszabdza**\_ -> http://www.w3.org/2001/XMLSchema#string *()*


**dsza**\_ -> http://www.w3.org/2001/XMLSchema#string *()*


**dsazdjz** #sqdsopq -> http://www.w3.org/2001/XMLSchema#string *()*


**dzezd**(*#sqdsq* | *#zqedzbx*) -> \_ *()*
:   **ndsbzh** #sqdsq -> http://www.w3.org/2001/XMLSchema#string *()*


    **sqnzkzn**(*#sqdsq* | *#zqedzbx*) -> http://www.w3.org/2001/XMLSchema#string *()*


    **zsbdgz** #sqdsq -> http://www.w3.org/2001/XMLSchema#nonNegativeInteger *()*

**zdsnzsdh** #dzshyuua -> http://www.w3.org/2001/XMLSchema#gYear *()*


**asndzsd** #dzshyuua -> http://www.w3.org/2001/XMLSchema#gMonth *()*


**dznadzh** #dzshyuua -> http://www.w3.org/2001/XMLSchema#gDay *()*


**zdzndh** #qzddj -> http://www.w3.org/2001/XMLSchema#string *()*


**zdnzadh** #qzddj -> http://www.w3.org/2001/XMLSchema#string *()*


**zadszabnds** #qzddj -> http://www.w3.org/2001/XMLSchema#string *()*


**sqnhdbdj** #dnqsb -> http://www.w3.org/2001/XMLSchema#nonNegativeInteger *()*


**mkalnshsq** #dnqsb -> http://www.w3.org/2001/XMLSchema#nonNegativeInteger *()*

## Individuals

<rdf:List@ttp://www.w3.org/1999/02/22-rdf-syntax-ns#nil>


<foaf:Person@a04570373>
:   - foaf:name = 'John-Jules Meyer'
    - foaf:firstName = 'John-Jules'
    - lastName = 'Meyer'

<foaf:Person@a43836633>
:   - foaf:name = 'Jeen Broekstra'
    - foaf:firstName = 'Jeen'
    - lastName = 'Broekstra'

<foaf:Person@a85228505>
:   - foaf:name = 'Alexander Mädche'
    - foaf:firstName = 'Alexander'
    - lastName = 'Mädche'

<foaf:Person@a48552212>
:   - foaf:name = 'Björn Schnizler'
    - foaf:firstName = 'Björn'
    - lastName = 'Schnizler'

<foaf:Person@a971541439>
:   - foaf:name = 'Alberto Trombetta'
    - foaf:firstName = 'Alberto'
    - lastName = 'Trombetta'

<foaf:Person@a11090777>
:   - foaf:name = 'Christine Parent'
    - foaf:firstName = 'Christine'
    - lastName = 'Parent'

<foaf:Person@a250331360>
:   - foaf:name = 'R. Schmidt'
    - foaf:firstName = 'R.'
    - lastName = 'Schmidt'

<foaf:Person@a79573306>
:   - foaf:name = 'York Sure'
    - foaf:firstName = 'York'
    - lastName = 'Sure'

<foaf:Person@a885257047>
:   - foaf:name = 'M. Punceva'
    - foaf:firstName = 'M.'
    - lastName = 'Punceva'

<foaf:Person@a74993404>
:   - foaf:name = 'I. V. Levenshtein'
    - foaf:firstName = 'I. V.'
    - lastName = 'Levenshtein'

<foaf:Person@a71003986>
:   - foaf:name = 'Steffen Staab'
    - foaf:firstName = 'Steffen'
    - lastName = 'Staab'

<foaf:Person@a572406328>
:   - foaf:name = 'Frank Boer'
    - foaf:firstName = 'Frank'
    - lastName = 'Boer'

<foaf:Person@a139477786>
:   - foaf:name = 'Maarten Menken'
    - foaf:firstName = 'Maarten'
    - lastName = 'Menken'

<foaf:Person@a337716610>
:   - foaf:name = 'Manfred Hauswirth'
    - foaf:firstName = 'Manfred'
    - lastName = 'Hauswirth'

<foaf:Person@a086379337>
:   - foaf:name = 'Wiebe Hoek'
    - foaf:firstName = 'Wiebe'
    - lastName = 'Hoek'

<foaf:Person@a712561038>
:   - foaf:name = 'Marc Ehrig'
    - foaf:firstName = 'Marc'
    - lastName = 'Ehrig'

<foaf:Person@a066600210>
:   - foaf:name = 'Danilo Montesi'
    - foaf:firstName = 'Danilo'
    - lastName = 'Montesi'

<foaf:Person@a093016135>
:   - foaf:name = 'Rogier Eijk'
    - foaf:firstName = 'Rogier'
    - lastName = 'Eijk'

<foaf:Person@a944339054>
:   - foaf:name = 'Frank van Harmelen'
    - foaf:firstName = 'Frank'
    - lastName = 'van Harmelen'

<foaf:Person@a98078619>
:   - foaf:name = 'Philippe Cudré-Mauroux'
    - foaf:firstName = 'Philippe'
    - lastName = 'Cudré-Mauroux'

<foaf:Person@a39510672>
:   - foaf:name = 'Z. Despotovic'
    - foaf:firstName = 'Z.'
    - lastName = 'Despotovic'

<foaf:Person@a431956276>
:   - foaf:name = 'Stefano Spaccapietra'
    - foaf:firstName = 'Stefano'
    - lastName = 'Spaccapietra'

<foaf:Person@a431956276b>
:   - foaf:name = 'Mike Papazoglou'
    - foaf:firstName = 'Mike'
    - lastName = 'Papazoglou'

<foaf:Person@a431956276c>
:   - foaf:name = 'Zahir Tari'
    - foaf:firstName = 'Zahir'
    - lastName = 'Tari'

<foaf:Person@a70955601>
:   - foaf:name = 'A. Datta'
    - foaf:firstName = 'A.'
    - lastName = 'Datta'

<foaf:Person@a467748807>
:   - foaf:name = 'Ateret Anaby-Tavor'
    - foaf:firstName = 'Ateret'
    - lastName = 'Anaby-Tavor'

<foaf:Person@a3105947>
:   - foaf:name = 'Ronny Siebes'
    - foaf:firstName = 'Ronny'
    - lastName = 'Siebes'

<foaf:Person@a29105611>
:   - foaf:name = 'Karl Aberer'
    - foaf:firstName = 'Karl'
    - lastName = 'Aberer'

<foaf:Person@a958684218>
:   - foaf:name = 'Peter Mika'
    - foaf:firstName = 'Peter'
    - lastName = 'Mika'

<foaf:Person@a94533498>
:   - foaf:name = 'Peter Haase'
    - foaf:firstName = 'Peter'
    - lastName = 'Haase'

<foaf:Person@a900366022>
:   - foaf:name = 'Avigdor Gal'
    - foaf:firstName = 'Avigdor'
    - lastName = 'Gal'

<qsdsquj@a246119474>
:   - foaf:name = 'Journal of Web Semantics'
    - dsza = 'JWS'

<zauio@a131020767>
:   - dszabdza = 'Springer-Verlag'
    - qzd =

      <qzddj@>
      :   - zdzndh = 'Heidelberg'
          - zadszabnds = 'DE'

<qsdsquj@a70981683>
:   - dszabdza = 'Cybernetics and Control Theory'

<zauio@a85849488>
:   - dszabdza = 'The MIT Press'
    - qzd =

      <qzddj@>
      :   - zdzndh = 'Cambridge'
          - zdnzadh = 'MA'
          - zadszabnds = 'US'

<qsdsquj@a362042121>
:   - dszabdza = 'International journal of intelligent system'
    - dsza = 'IJIS'

<qsdsquj@a674639524>
:   - dszabdza = 'ACM SIGMOD Record'

<qsdsquj@a906774044>
:   - dszabdza = 'VLDB Journal'

<zqedzbx@spg04>
:   - dszabdza = 'SemPGrid 04 Workshop'
    - zdjznzn =

      <qzddj@>
      :   - zdzndh = 'New-York'
          - zdnzadh = 'NY'
          - zadszabnds = 'US'
    - sqndsqgy =

      <dzshyuua@>
      :   - asndzsd = '--05'
          - zdsnzsdh = '2004'

<zqedzbx@a72192307c>
:   - dszabdza = 'Int. Conference on Knowledge Engineering and Management'
    - dsza = 'EKAW'
    - sqnzkzn = '13'
    - sqndsqgy =

      <dzshyuua@>
      :   - asndzsd = '--10'
          - zdsnzsdh = '2002'

<zqedzbx@a32071928c>
:   - dszabdza = 'European Semantic Web Symposium'
    - dsza = 'ESWS'
    - sqnzkzn = '1'
    - zdjznzn =

      <qzddj@>
      :   - zdzndh = 'Heraklion'
          - zadszabnds = 'GR'
    - sqndsqgy =

      <dzshyuua@>
      :   - asndzsd = '--05'
          - zdsnzsdh = '2004'

<zdqssqdb@a060097576>
:   - dznbaln = 'Proceedings of the SemPGrid 04 Workshop'
    - sqndsqgy =

      <dzshyuua@>
      :   - zdsnzsdh = '2004'
    - zadzjadns = <\_@#spg04>

<deqdxcsqcsq@a64263824>
:   - zand =

      <dsqdbz@>
      :   - rdf:first = <\_@#a43836633>
          - rdf:rest =

            <dsqdbz@>
            :   - rdf:first = <\_@#a712561038>
                - rdf:rest =

                  <dsqdbz@>
                  :   - rdf:first = <\_@#a94533498>
                      - rdf:rest =

                        <dsqdbz@>
                        :   - rdf:first = <\_@#a944339054>
                            - rdf:rest =

                              <dsqdbz@>
                              :   - rdf:first = <\_@#a139477786>
                                  - rdf:rest =

                                    <dsqdbz@>
                                    :   - rdf:first = <\_@#a958684218>
                                        - rdf:rest =

                                          <dsqdbz@>
                                          :   - rdf:first = <\_@#a48552212>
                                              - rdf:rest =

                                                <dsqdbz@>
                                                :   - rdf:first = <\_@#a3105947>
                                                    - rdf:rest = <\_@http://www.w3.org/1999/02/22-rdf-syntax-ns#nil>
    - zassdzadb = <\_@#a060097576>
    - dznbaln = 'Bibster - A Semantics-Based Bibliographic Peer-to-Peer System'

<deqdxcsqcsq@a439508789>
:   - zand =

      <dsqdbz@>
      :   - rdf:first = <\_@#a85228505>
          - rdf:rest =

            <dsqdbz@>
            :   - rdf:first = <\_@#a71003986>
                - rdf:rest = <\_@http://www.w3.org/1999/02/22-rdf-syntax-ns#nil>
    - zassdzadb = <\_@#a72192307>
    - dznbaln = 'Measuring Similarity between Ontologies'

<hazdn@a492378321>
:   - zand =

      <dsqdbz@>
      :   - rdf:first = <\_@#a29105611>
          - rdf:rest =

            <dsqdbz@>
            :   - rdf:first = <\_@#a98078619>
                - rdf:rest =

                  <dsqdbz@>
                  :   - rdf:first = <\_@#a70955601>
                      - rdf:rest =

                        <dsqdbz@>
                        :   - rdf:first = <\_@#a39510672>
                            - rdf:rest =

                              <dsqdbz@>
                              :   - rdf:first = <\_@#a337716610>
                                  - rdf:rest =

                                    <dsqdbz@>
                                    :   - rdf:first = <\_@#a885257047>
                                        - rdf:rest =

                                          <dsqdbz@>
                                          :   - rdf:first = <\_@#a250331360>
                                              - rdf:rest = <\_@http://www.w3.org/1999/02/22-rdf-syntax-ns#nil>
    - sxqsnbvsq = <\_@#a674639524>
    - dznbaln = '{P-Grid}: A Self-organizing Structured P2P System'
    - sqndsqgy =

      <dzshyuua@>
      :   - zdsnzsdh = '2003'

<hazdn@a475526642>
:   - zand =

      <dsqdbz@>
      :   - rdf:first = <\_@#a74993404>
          - rdf:rest = <\_@http://www.w3.org/1999/02/22-rdf-syntax-ns#nil>
    - sxqsnbvsq = <\_@#a70981683>
    - dznbaln = 'Binary Codes capable of correcting deletions, insertions, and reversals'
    - sqndsqgy =

      <dzshyuua@>
      :   - zdsnzsdh = '1996'

<vccfsq@a71568377>
:   - zand =

      <dsqdbz@>
      :   - rdf:first = <\_@#a11090777>
          - rdf:rest =

            <dsqdbz@>
            :   - rdf:first = <\_@#a431956276>
                - rdf:rest = <\_@http://www.w3.org/1999/02/22-rdf-syntax-ns#nil>
    - sznsbq = <\_@#a108048723>
    - dznbaln = 'Database integration: the key to data interoperability'
    - dzajj =

      <dsqdbz@>
      :   - rdf:first = <\_@#a431956276>
          - rdf:rest =

            <dsqdbz@>
            :   - rdf:first = <\_@#a431956276b>
                - rdf:rest =

                  <dsqdbz@>
                  :   - rdf:first = <\_@#a431956276c>
                      - rdf:rest = <\_@http://www.w3.org/1999/02/22-rdf-syntax-ns#nil>

<zdqssqdb@a72192307>
:   - sddnsnqdb = <\_@#a131020767>
    - dznbaln = 'Proc. Of the 13th Int. Conference on Knowledge Engineering and Management (EKAW-2002)'
    - zadzjadns = <\_@#a72192307c>
    - sqndsqgy =

      <dzshyuua@>
      :   - zdsnzsdh = '2002'

<zdqssqdb@a32071928>
:   - sddnsnqdb = <\_@#a131020767>
    - zadzjadns = <\_@#a32071928c>
    - dznbaln = 'Proceedings of the First European Semantic Web Symposium'
    - sqndsqgy =

      <dzshyuua@>
      :   - zdsnzsdh = '2004'

<wxsdxqsdh@a140583454>
:   - zand =

      <dsqdbz@>
      :   - rdf:first = <\_@#a712561038>
          - rdf:rest =

            <dsqdbz@>
            :   - rdf:first = <\_@#a71003986>
                - rdf:rest = <\_@http://www.w3.org/1999/02/22-rdf-syntax-ns#nil>
    - dznbaln = '{QOM} - Quick Ontology Mapping'
    - zdsnsqdv = 'submitted to the ISWC 04'
    - sqndsqgy =

      <dzshyuua@>
      :   - zdsnzsdh = '2004'

<deqdxcsqcsq@a11065952>
:   - zand =

      <dsqdbz@>
      :   - rdf:first = <\_@#a712561038>
          - rdf:rest =

            <dsqdbz@>
            :   - rdf:first = <\_@#a79573306>
                - rdf:rest = <\_@http://www.w3.org/1999/02/22-rdf-syntax-ns#nil>
    - zassdzadb = <\_@#a32071928>
    - dznbaln = 'Ontology Mapping - An Integrated Approach'
    - zdsznbadzagh = 'http://www.aifb.uni-karlsruhe.de/WBS/meh/publications/ehrig04ontology\_ESWS04.pdf'

<hazdn@a80299267>
:   - zand =

      <dsqdbz@>
      :   - rdf:first = <\_@#a29105611>
          - rdf:rest =

            <dsqdbz@>
            :   - rdf:first = <\_@#a98078619>
                - rdf:rest =

                  <dsqdbz@>
                  :   - rdf:first = <\_@#a337716610>
                      - rdf:rest = <\_@http://www.w3.org/1999/02/22-rdf-syntax-ns#nil>
    - sxqsnbvsq = <\_@#a246119474>
    - dznbaln = 'Start making sense: The Chatty Web approach for global semantic agreements'
    - sqndsqgy =

      <dzshyuua@>
      :   - asndzsd = '--12'
          - zdsnzsdh = '2003'

<dzajndsq@a108048723>
:   - sddnsnqdb = <\_@#a85849488>
    - dznbaln = 'Object-Oriented Data Modeling'
    - sqndsqgy =

      <dzshyuua@>
      :   - zdsnzsdh = '2000'

<hazdn@a456080390>
:   - zand =

      <dsqdbz@>
      :   - rdf:first = <\_@#a093016135>
          - rdf:rest =

            <dsqdbz@>
            :   - rdf:first = <\_@#a572406328>
                - rdf:rest =

                  <dsqdbz@>
                  :   - rdf:first = <\_@#a086379337>
                      - rdf:rest =

                        <dsqdbz@>
                        :   - rdf:first = <\_@#a04570373>
                            - rdf:rest = <\_@http://www.w3.org/1999/02/22-rdf-syntax-ns#nil>
    - sxqsnbvsq = <\_@#a362042121>
    - dznbaln = 'On dynamically generated ontology translators in agent communication'
    - dzayue =

      <dnqsb@>
      :   - sqnhdbdj = '587'
          - mkalnshsq = '607'
    - sqndsqgy =

      <dzshyuua@>
      :   - asndzsd = '--12'
          - zdsnzsdh = '2001'

<hazdn@a846015923>
:   - zand =

      <dsqdbz@>
      :   - rdf:first = <\_@#a900366022>
          - rdf:rest =

            <dsqdbz@>
            :   - rdf:first = <\_@#a467748807>
                - rdf:rest =

                  <dsqdbz@>
                  :   - rdf:first = <\_@#a971541439>
                      - rdf:rest =

                        <dsqdbz@>
                        :   - rdf:first = <\_@#a066600210>
                            - rdf:rest = <\_@http://www.w3.org/1999/02/22-rdf-syntax-ns#nil>
    - sxqsnbvsq = <\_@#a906774044>
    - dznbaln = 'A Framework for Modeling and Evaluating Automatic Semantic Reconciliation'
    - zdsnsqdv = 'to appear'
    - sqndsqgy =

      <dzshyuua@>
      :   - zdsnzsdh = '2004'

---

Generated by OWL2HTML
