# Supplementary material for: Matching sensor ontologies through siamese neural networks without using reference alignment
Source: PeerJ Comput Sci. 2021 Jun 18;7:e602. doi: 10.7717/peerj-cs.602 (PMC8237319; doi:10.7717/peerj-cs.602)
Supplement: Supplemental Information 1 [file peerj-cs-07-602-s001.zip › 204/refalign.html]

# (level 0) Alignment

## Source: http://oaei.ontologymatching.org/2011/benchmarks/101/onto.rdf

## Target: http://oaei.ontologymatching.org/2011/benchmarks/204/onto.rdf

## Correspondences

type = type
:   1.0

howPublished = how\_published
:   1.0

periodicity = periodicity
:   1.0

proceedings = proceedings
:   1.0

volume = volume
:   1.0

annote = annote
:   1.0

PersonList = PERSON\_LIST
:   1.0

month = month
:   1.0

copyright = copyright
:   1.0

Unpublished = Un\_published
:   1.0

address = address
:   1.0

Address = ADDRESS
:   1.0

chapter = chapter
:   1.0

Chapter = Chapter
:   1.0

editor = editor
:   1.0

InBook = In\_book
:   1.0

Date = DATE
:   1.0

series = series
:   1.0

PageRange = PAGE\_RANGE
:   1.0

date = date
:   1.0

title = title
:   1.0

Booklet = Booklet
:   1.0

numberOrVolume = number\_or\_volume
:   1.0

LectureNotes = Lecture\_notes
:   1.0

url = U.R.L.
:   1.0

MastersThesis = MSc\_thesis
:   1.0

organizer = organiser
:   1.0

mrNumber = M.R.Nb
:   1.0

pages = pages
:   1.0

TechReport = Technical\_report
:   1.0

reviewed = reviewed
:   1.0

startPage = start\_page
:   1.0

edition = edition
:   1.0

lccn = L.C.C.N.
:   1.0

affiliation = affiliation
:   1.0

institution = institution
:   1.0

year = year
:   1.0

isPartOf = is\_part\_of
:   1.0

organization = organisation
:   1.0

country = country
:   1.0

publisher = publisher
:   1.0

school = school
:   1.0

Misc = Misc.
:   1.0

collection = collection
:   1.0

Collection = Collection
:   1.0

isbn = I.S.B.N.
:   1.0

abstract = abstract
:   1.0

directors = directors
:   1.0

Academic = Academic
:   1.0

location = location
:   1.0

MotionPicture = Motion\_picture
:   1.0

Article = Article
:   1.0

Informal = Informal
:   1.0

price = price
:   1.0

name = name
:   1.0

event = event
:   1.0

state = state
:   1.0

Book = Book
:   1.0

book = book
:   1.0

day = day
:   1.0

School = School
:   1.0

shortName = short\_name
:   1.0

PhdThesis = PhD\_thesis
:   1.0

Proceedings = Proc.
:   1.0

number = number
:   1.0

issue = issue
:   1.0

Reference = REFERENCE
:   1.0

endPage = end\_page
:   1.0

InCollection = In\_collection
:   1.0

firstPublished = first\_published
:   1.0

author = author
:   1.0

Report = Report
:   1.0

note = note
:   1.0

humanCreator = human\_creator
:   1.0

Conference = CONFERENCE
:   1.0

Part = Part
:   1.0

Publisher = Publisher
:   1.0

contract = contract
:   1.0

Manual = Manual
:   1.0

key = key
:   1.0

InProceedings = In\_proceedings
:   1.0

Journal = JOURNAL
:   1.0

journal = journal
:   1.0

Monograph = Monograph
:   1.0

keywords = keywords
:   1.0

issn = I.S.S.N.
:   1.0

contents = contents
:   1.0

city = city
:   1.0

Institution = Institution
:   1.0

Deliverable = Deliverable
:   1.0

size = size
:   1.0

chapters = chap
:   1.0

parts = parts
:   1.0

communications = com
:   1.0

articles = art
:   1.0

lastName = lastName
:   1.0

language = language
:   1.0
