# Supplementary material for: Matching sensor ontologies through siamese neural networks without using reference alignment
Source: PeerJ Comput Sci. 2021 Jun 18;7:e602. doi: 10.7717/peerj-cs.602 (PMC8237319; doi:10.7717/peerj-cs.602)
Supplement: Supplemental Information 1 [file peerj-cs-07-602-s001.zip › 208/onto.html]

# 

Author: Nick Knouf <nknouf@mit.edu>  
Contributor: Antoine Zimmermann <antoine.zimmermann@inrialpes.fr>, Jérôme Euzenat,   
Date: 08/06/2005  
Version: $Id: onto.rdf,v 1.30 2008/05/27 14:41:13 euzenat Exp $

## Classes

**http://www.w3.org/1999/02/22-rdf-syntax-ns#List** (, *)*


**http://xmlns.com/foaf/0.1/Person** (, *)*


**http://xmlns.com/foaf/0.1/Organization** (, *)*


**REFERENCE** (, *)*
:   - #date [0 1]
    - #title [0 1]
    - #human\_creator [0 1]

    **Book** (, *)*
    :   - #title [1 1]
        - #volume [0 1]
        - #publisher [0 1]
        - #series [0 1]
        - #date [1 1] *#DATE*
        - #author [1 1]
        - #edition [0 1]

        **Monograph** (, *)*
        :   - #chap *#Chapter*

        **Collection** (, *)*
        :   - #chap *#Chapter*
            - #parts *#In\_collection*

        **Proc.** (, *)*
        :   - #com *#In\_proceedings*
            - #event [0 1] *#CONFERENCE*
            - #editor [0 1]
            - #organisation [0 1]

    **Informal** (, *)*
    :   - #title [1 1]

        **Booklet** (, *)*


        **Lecture\_notes** (, *)*


        **Manual** (, *)*
        :   - #organisation [0 1]
            - #edition [0 1]
            - #title [1 1]

        **Un\_published** (, *)*
        :   - #author [1 1]
            - #title [1 1]
            - #note [1 +oo]

    **Part** (, *)*
    :   - #pages [0 1]
        - #title [1 1]

        **Article** (, *)*
        :   - #author [1 1]
            - #pages [1 1]
            - #journal [1 1]
            - #date [1 1] *#DATE*
            - #number [0 1]
            - #volume [0 1]

        **Chapter** (, *)*
        :   - #chapter [0 1] [0 1]

        **In\_book** (, *)*
        :   - #author [1 1]
            - #pages [1 +oo]
            - #book [1 1]

        **In\_collection** (, *)*
        :   - #author [1 1]
            - #collection [1 1]

        **In\_proceedings** (, *)*
        :   - #author [1 1]
            - #proceedings [1 1]

    **Academic** (, *)*
    :   - #author [1 1]
        - #title [1 1]
        - #school [1 1]
        - #date [1 1]

        **MSc\_thesis** (, *)*


        **PhD\_thesis** (, *)*

    **Misc.** (, *)*


    **Report** (, *)*
    :   - #author [1 1]
        - #title [1 1]
        - #institution [1 1]
        - #date [1 1] *#DATE*
        - #number [0 1]

        **Technical\_report** (, *)*


        **Deliverable** (, *)*
        :   - #contract [0 1]

    **Motion\_picture** (, *)*

**JOURNAL** (, *)*
:   - #name [1 1] *http://www.w3.org/2001/XMLSchema#string*
    - #short\_name *http://www.w3.org/2001/XMLSchema#string*
    - #periodicity *http://www.w3.org/2001/XMLSchema#string*
    - #publisher [0 1]
    - #series [0 1]
    - #first\_published [0 1]
    - #art *#Article*

**CONFERENCE** (, *)*
:   - #name [1 1]
    - #organiser *#Institution*
    - #short\_name [0 1]
    - #issue [0 1]
    - #location [0 1]

**ADDRESS** (, *)*
:   - #country [0 1] *http://www.w3.org/2001/XMLSchema#string*
    - #state [0 1] *http://www.w3.org/2001/XMLSchema#string*
    - #city [0 1] *http://www.w3.org/2001/XMLSchema#string*

**Institution** (, *)*
:   super: *http://xmlns.com/foaf/0.1/Organization*  

    - #name [1 1]
    - #short\_name [1 1]
    - #address [0 1]

    **Publisher** (, *)*


    **School** (, *)*

**PERSON\_LIST** (, *)*
:   super: *http://www.w3.org/1999/02/22-rdf-syntax-ns#List*  

    - http://www.w3.org/1999/02/22-rdf-syntax-ns#first [1 1] *http://xmlns.com/foaf/0.1/Person*
    - http://www.w3.org/1999/02/22-rdf-syntax-ns#rest [1 1] (*#PERSON\_LIST* |  {

      <rdf:List@ttp://www.w3.org/1999/02/22-rdf-syntax-ns#nil>
      } )

**PAGE\_RANGE** (, *)*
:   - #start\_page [1 1]
    - #end\_page [1 1]

**DATE** (, *)*
:   - #year [1 1] *http://www.w3.org/2001/XMLSchema#gYear*
    - #month [0 1] *http://www.w3.org/2001/XMLSchema#gMonth*
    - #day [0 1] *http://www.w3.org/2001/XMLSchema#gDay*

## Properties

**http://www.w3.org/1999/02/22-rdf-syntax-ns#first**: http://www.w3.org/1999/02/22-rdf-syntax-ns#List -> \_ *()*


**http://www.w3.org/1999/02/22-rdf-syntax-ns#rest**: http://www.w3.org/1999/02/22-rdf-syntax-ns#List -> http://www.w3.org/1999/02/22-rdf-syntax-ns#List *()*


**chap**: #REFERENCE -> #Chapter *()*


**parts**: #REFERENCE -> #Part *()*


**com**: #Proc. -> #In\_proceedings *()*


**art**: #JOURNAL -> #Article *()*


**address**: http://www.w3.org/2002/07/owl#Thing -> #ADDRESS *()*


**event**: #Proc. -> #CONFERENCE *()*


**organiser**: #CONFERENCE -> http://xmlns.com/foaf/0.1/Organization *()*


**contract**: #REFERENCE -> http://www.w3.org/2002/07/owl#Thing *()*


**human\_creator**: #REFERENCE -> #PERSON\_LIST *()*
:   **author**: \_ -> \_ *()*


    **editor**: \_ -> \_ *()*


    **directors**: #Motion\_picture -> \_ *()*

**institution**: #Report -> #Institution *()*


**is\_part\_of**: #Part -> \_ *()*
:   **journal**: #Article -> #JOURNAL *()*


    **book**: #In\_book -> #Monograph *()*


    **collection**: #In\_collection -> #Collection *()*


    **proceedings**: #In\_proceedings -> #Proc. *()*

**date**: (*#REFERENCE* | *#CONFERENCE*) -> #DATE *()*


**organisation**: (*#Proc.* | *#Manual*) -> http://xmlns.com/foaf/0.1/Organization *()*


**publisher**: (*#REFERENCE* | *#JOURNAL*) -> #Publisher *()*


**school**: (*#Academic* | *#Lecture\_notes*) -> #School *()*


**location**: (*#REFERENCE* | *#CONFERENCE*) -> #ADDRESS *()*


**pages**: #Part -> #PAGE\_RANGE *()*

**http://purl.org/dc/elements/1.1/creator**\_ -> \_ *()*


**http://purl.org/dc/elements/1.1/contributor**\_ -> \_ *()*


**http://purl.org/dc/elements/1.1/description**\_ -> \_ *()*


**http://purl.org/dc/elements/1.1/date**\_ -> \_ *()*


**http://xmlns.com/foaf/0.1/firstName**\_ -> \_ *()*


**lastName**\_ -> \_ *()*


**http://xmlns.com/foaf/0.1/name**\_ -> \_ *()*


**key** #REFERENCE -> http://www.w3.org/2001/XMLSchema#string *()*


**reviewed** #REFERENCE -> http://www.w3.org/2001/XMLSchema#string *()*


**annote** #REFERENCE -> http://www.w3.org/2001/XMLSchema#string *()*


**periodicity** #JOURNAL -> http://www.w3.org/2001/XMLSchema#string *()*


**first\_published** #Chapter -> http://www.w3.org/2001/XMLSchema#string *()*


**edition**(*#Book* | *#Manual*) -> http://www.w3.org/2001/XMLSchema#string *()*


**how\_published**(*#Misc.* | *#Booklet*) -> http://www.w3.org/2001/XMLSchema#string *()*


**note** #REFERENCE -> http://www.w3.org/2001/XMLSchema#string *()*


**series** #REFERENCE -> http://www.w3.org/2001/XMLSchema#string *()*


**title** #REFERENCE -> http://www.w3.org/2001/XMLSchema#string *()*


**type**(*#Chapter* | *#Technical\_report* | *#Academic*) -> http://www.w3.org/2001/XMLSchema#string *()*


**affiliation** #REFERENCE -> http://www.w3.org/2001/XMLSchema#string *()*


**abstract** #REFERENCE -> http://www.w3.org/2001/XMLSchema#string *()*


**contents** #REFERENCE -> http://www.w3.org/2001/XMLSchema#string *()*


**copyright** #REFERENCE -> http://www.w3.org/2001/XMLSchema#string *()*


**I.S.B.N.** #REFERENCE -> http://www.w3.org/2001/XMLSchema#string *()*


**I.S.S.N.** #REFERENCE -> http://www.w3.org/2001/XMLSchema#string *()*


**keywords** #REFERENCE -> http://www.w3.org/2001/XMLSchema#string *()*


**language** #REFERENCE -> http://www.w3.org/2001/XMLSchema#language *()*


**L.C.C.N.** #REFERENCE -> http://www.w3.org/2001/XMLSchema#string *()*


**M.R.Nb** #REFERENCE -> http://www.w3.org/2001/XMLSchema#string *()*


**price** #REFERENCE -> http://www.w3.org/2001/XMLSchema#string *()*


**size** #REFERENCE -> http://www.w3.org/2001/XMLSchema#string *()*


**U.R.L.** #REFERENCE -> http://www.w3.org/2001/XMLSchema#string *()*


**name**\_ -> http://www.w3.org/2001/XMLSchema#string *()*


**short\_name**\_ -> http://www.w3.org/2001/XMLSchema#string *()*


**chapter** #Part -> http://www.w3.org/2001/XMLSchema#string *()*


**number\_or\_volume**(*#REFERENCE* | *#CONFERENCE*) -> \_ *()*
:   **number** #REFERENCE -> http://www.w3.org/2001/XMLSchema#string *()*


    **issue**(*#REFERENCE* | *#CONFERENCE*) -> http://www.w3.org/2001/XMLSchema#string *()*


    **volume** #REFERENCE -> http://www.w3.org/2001/XMLSchema#nonNegativeInteger *()*

**year** #DATE -> http://www.w3.org/2001/XMLSchema#gYear *()*


**month** #DATE -> http://www.w3.org/2001/XMLSchema#gMonth *()*


**day** #DATE -> http://www.w3.org/2001/XMLSchema#gDay *()*


**city** #ADDRESS -> http://www.w3.org/2001/XMLSchema#string *()*


**state** #ADDRESS -> http://www.w3.org/2001/XMLSchema#string *()*


**country** #ADDRESS -> http://www.w3.org/2001/XMLSchema#string *()*


**start\_page** #PAGE\_RANGE -> http://www.w3.org/2001/XMLSchema#nonNegativeInteger *()*


**end\_page** #PAGE\_RANGE -> http://www.w3.org/2001/XMLSchema#nonNegativeInteger *()*

## Individuals

<rdf:List@ttp://www.w3.org/1999/02/22-rdf-syntax-ns#nil>


<foaf:Person@a04570373>
:   - foaf:name = 'John-Jules Meyer'
    - foaf:firstName = 'John-Jules'
    - lastName = 'Meyer'

<foaf:Person@a43836633>
:   - foaf:name = 'Jeen Broekstra'
    - foaf:firstName = 'Jeen'
    - lastName = 'Broekstra'

<foaf:Person@a85228505>
:   - foaf:name = 'Alexander Mädche'
    - foaf:firstName = 'Alexander'
    - lastName = 'Mädche'

<foaf:Person@a48552212>
:   - foaf:name = 'Björn Schnizler'
    - foaf:firstName = 'Björn'
    - lastName = 'Schnizler'

<foaf:Person@a971541439>
:   - foaf:name = 'Alberto Trombetta'
    - foaf:firstName = 'Alberto'
    - lastName = 'Trombetta'

<foaf:Person@a11090777>
:   - foaf:name = 'Christine Parent'
    - foaf:firstName = 'Christine'
    - lastName = 'Parent'

<foaf:Person@a250331360>
:   - foaf:name = 'R. Schmidt'
    - foaf:firstName = 'R.'
    - lastName = 'Schmidt'

<foaf:Person@a79573306>
:   - foaf:name = 'York Sure'
    - foaf:firstName = 'York'
    - lastName = 'Sure'

<foaf:Person@a885257047>
:   - foaf:name = 'M. Punceva'
    - foaf:firstName = 'M.'
    - lastName = 'Punceva'

<foaf:Person@a74993404>
:   - foaf:name = 'I. V. Levenshtein'
    - foaf:firstName = 'I. V.'
    - lastName = 'Levenshtein'

<foaf:Person@a71003986>
:   - foaf:name = 'Steffen Staab'
    - foaf:firstName = 'Steffen'
    - lastName = 'Staab'

<foaf:Person@a572406328>
:   - foaf:name = 'Frank Boer'
    - foaf:firstName = 'Frank'
    - lastName = 'Boer'

<foaf:Person@a139477786>
:   - foaf:name = 'Maarten Menken'
    - foaf:firstName = 'Maarten'
    - lastName = 'Menken'

<foaf:Person@a337716610>
:   - foaf:name = 'Manfred Hauswirth'
    - foaf:firstName = 'Manfred'
    - lastName = 'Hauswirth'

<foaf:Person@a086379337>
:   - foaf:name = 'Wiebe Hoek'
    - foaf:firstName = 'Wiebe'
    - lastName = 'Hoek'

<foaf:Person@a712561038>
:   - foaf:name = 'Marc Ehrig'
    - foaf:firstName = 'Marc'
    - lastName = 'Ehrig'

<foaf:Person@a066600210>
:   - foaf:name = 'Danilo Montesi'
    - foaf:firstName = 'Danilo'
    - lastName = 'Montesi'

<foaf:Person@a093016135>
:   - foaf:name = 'Rogier Eijk'
    - foaf:firstName = 'Rogier'
    - lastName = 'Eijk'

<foaf:Person@a944339054>
:   - foaf:name = 'Frank van Harmelen'
    - foaf:firstName = 'Frank'
    - lastName = 'van Harmelen'

<foaf:Person@a98078619>
:   - foaf:name = 'Philippe Cudré-Mauroux'
    - foaf:firstName = 'Philippe'
    - lastName = 'Cudré-Mauroux'

<foaf:Person@a39510672>
:   - foaf:name = 'Z. Despotovic'
    - foaf:firstName = 'Z.'
    - lastName = 'Despotovic'

<foaf:Person@a431956276>
:   - foaf:name = 'Stefano Spaccapietra'
    - foaf:firstName = 'Stefano'
    - lastName = 'Spaccapietra'

<foaf:Person@a431956276b>
:   - foaf:name = 'Mike Papazoglou'
    - foaf:firstName = 'Mike'
    - lastName = 'Papazoglou'

<foaf:Person@a431956276c>
:   - foaf:name = 'Zahir Tari'
    - foaf:firstName = 'Zahir'
    - lastName = 'Tari'

<foaf:Person@a70955601>
:   - foaf:name = 'A. Datta'
    - foaf:firstName = 'A.'
    - lastName = 'Datta'

<foaf:Person@a467748807>
:   - foaf:name = 'Ateret Anaby-Tavor'
    - foaf:firstName = 'Ateret'
    - lastName = 'Anaby-Tavor'

<foaf:Person@a3105947>
:   - foaf:name = 'Ronny Siebes'
    - foaf:firstName = 'Ronny'
    - lastName = 'Siebes'

<foaf:Person@a29105611>
:   - foaf:name = 'Karl Aberer'
    - foaf:firstName = 'Karl'
    - lastName = 'Aberer'

<foaf:Person@a958684218>
:   - foaf:name = 'Peter Mika'
    - foaf:firstName = 'Peter'
    - lastName = 'Mika'

<foaf:Person@a94533498>
:   - foaf:name = 'Peter Haase'
    - foaf:firstName = 'Peter'
    - lastName = 'Haase'

<foaf:Person@a900366022>
:   - foaf:name = 'Avigdor Gal'
    - foaf:firstName = 'Avigdor'
    - lastName = 'Gal'

<JOURNAL@a246119474>
:   - foaf:name = 'Journal of Web Semantics'
    - short\_name = 'JWS'

<Publisher@a131020767>
:   - name = 'Springer-Verlag'
    - address =

      <ADDRESS@>
      :   - city = 'Heidelberg'
          - country = 'DE'

<JOURNAL@a70981683>
:   - name = 'Cybernetics and Control Theory'

<Publisher@a85849488>
:   - name = 'The MIT Press'
    - address =

      <ADDRESS@>
      :   - city = 'Cambridge'
          - state = 'MA'
          - country = 'US'

<JOURNAL@a362042121>
:   - name = 'International journal of intelligent system'
    - short\_name = 'IJIS'

<JOURNAL@a674639524>
:   - name = 'ACM SIGMOD Record'

<JOURNAL@a906774044>
:   - name = 'VLDB Journal'

<CONFERENCE@spg04>
:   - name = 'SemPGrid 04 Workshop'
    - location =

      <ADDRESS@>
      :   - city = 'New-York'
          - state = 'NY'
          - country = 'US'
    - date =

      <DATE@>
      :   - month = '--05'
          - year = '2004'

<CONFERENCE@a72192307c>
:   - name = 'Int. Conference on Knowledge Engineering and Management'
    - short\_name = 'EKAW'
    - issue = '13'
    - date =

      <DATE@>
      :   - month = '--10'
          - year = '2002'

<CONFERENCE@a32071928c>
:   - name = 'European Semantic Web Symposium'
    - short\_name = 'ESWS'
    - issue = '1'
    - location =

      <ADDRESS@>
      :   - city = 'Heraklion'
          - country = 'GR'
    - date =

      <DATE@>
      :   - month = '--05'
          - year = '2004'

<Proc.@a060097576>
:   - title = 'Proceedings of the SemPGrid 04 Workshop'
    - date =

      <DATE@>
      :   - year = '2004'
    - event = <\_@#spg04>

<In\_proceedings@a64263824>
:   - author =

      <PERSON\_LIST@>
      :   - rdf:first = <\_@#a43836633>
          - rdf:rest =

            <PERSON\_LIST@>
            :   - rdf:first = <\_@#a712561038>
                - rdf:rest =

                  <PERSON\_LIST@>
                  :   - rdf:first = <\_@#a94533498>
                      - rdf:rest =

                        <PERSON\_LIST@>
                        :   - rdf:first = <\_@#a944339054>
                            - rdf:rest =

                              <PERSON\_LIST@>
                              :   - rdf:first = <\_@#a139477786>
                                  - rdf:rest =

                                    <PERSON\_LIST@>
                                    :   - rdf:first = <\_@#a958684218>
                                        - rdf:rest =

                                          <PERSON\_LIST@>
                                          :   - rdf:first = <\_@#a48552212>
                                              - rdf:rest =

                                                <PERSON\_LIST@>
                                                :   - rdf:first = <\_@#a3105947>
                                                    - rdf:rest = <\_@http://www.w3.org/1999/02/22-rdf-syntax-ns#nil>
    - proceedings = <\_@#a060097576>
    - title = 'Bibster - A Semantics-Based Bibliographic Peer-to-Peer System'

<In\_proceedings@a439508789>
:   - author =

      <PERSON\_LIST@>
      :   - rdf:first = <\_@#a85228505>
          - rdf:rest =

            <PERSON\_LIST@>
            :   - rdf:first = <\_@#a71003986>
                - rdf:rest = <\_@http://www.w3.org/1999/02/22-rdf-syntax-ns#nil>
    - proceedings = <\_@#a72192307>
    - title = 'Measuring Similarity between Ontologies'

<Article@a492378321>
:   - author =

      <PERSON\_LIST@>
      :   - rdf:first = <\_@#a29105611>
          - rdf:rest =

            <PERSON\_LIST@>
            :   - rdf:first = <\_@#a98078619>
                - rdf:rest =

                  <PERSON\_LIST@>
                  :   - rdf:first = <\_@#a70955601>
                      - rdf:rest =

                        <PERSON\_LIST@>
                        :   - rdf:first = <\_@#a39510672>
                            - rdf:rest =

                              <PERSON\_LIST@>
                              :   - rdf:first = <\_@#a337716610>
                                  - rdf:rest =

                                    <PERSON\_LIST@>
                                    :   - rdf:first = <\_@#a885257047>
                                        - rdf:rest =

                                          <PERSON\_LIST@>
                                          :   - rdf:first = <\_@#a250331360>
                                              - rdf:rest = <\_@http://www.w3.org/1999/02/22-rdf-syntax-ns#nil>
    - journal = <\_@#a674639524>
    - title = '{P-Grid}: A Self-organizing Structured P2P System'
    - date =

      <DATE@>
      :   - year = '2003'

<Article@a475526642>
:   - author =

      <PERSON\_LIST@>
      :   - rdf:first = <\_@#a74993404>
          - rdf:rest = <\_@http://www.w3.org/1999/02/22-rdf-syntax-ns#nil>
    - journal = <\_@#a70981683>
    - title = 'Binary Codes capable of correcting deletions, insertions, and reversals'
    - date =

      <DATE@>
      :   - year = '1996'

<In\_book@a71568377>
:   - author =

      <PERSON\_LIST@>
      :   - rdf:first = <\_@#a11090777>
          - rdf:rest =

            <PERSON\_LIST@>
            :   - rdf:first = <\_@#a431956276>
                - rdf:rest = <\_@http://www.w3.org/1999/02/22-rdf-syntax-ns#nil>
    - book = <\_@#a108048723>
    - title = 'Database integration: the key to data interoperability'
    - editor =

      <PERSON\_LIST@>
      :   - rdf:first = <\_@#a431956276>
          - rdf:rest =

            <PERSON\_LIST@>
            :   - rdf:first = <\_@#a431956276b>
                - rdf:rest =

                  <PERSON\_LIST@>
                  :   - rdf:first = <\_@#a431956276c>
                      - rdf:rest = <\_@http://www.w3.org/1999/02/22-rdf-syntax-ns#nil>

<Proc.@a72192307>
:   - publisher = <\_@#a131020767>
    - title = 'Proc. Of the 13th Int. Conference on Knowledge Engineering and Management (EKAW-2002)'
    - event = <\_@#a72192307c>
    - date =

      <DATE@>
      :   - year = '2002'

<Proc.@a32071928>
:   - publisher = <\_@#a131020767>
    - event = <\_@#a32071928c>
    - title = 'Proceedings of the First European Semantic Web Symposium'
    - date =

      <DATE@>
      :   - year = '2004'

<Misc.@a140583454>
:   - author =

      <PERSON\_LIST@>
      :   - rdf:first = <\_@#a712561038>
          - rdf:rest =

            <PERSON\_LIST@>
            :   - rdf:first = <\_@#a71003986>
                - rdf:rest = <\_@http://www.w3.org/1999/02/22-rdf-syntax-ns#nil>
    - title = '{QOM} - Quick Ontology Mapping'
    - note = 'submitted to the ISWC 04'
    - date =

      <DATE@>
      :   - year = '2004'

<In\_proceedings@a11065952>
:   - author =

      <PERSON\_LIST@>
      :   - rdf:first = <\_@#a712561038>
          - rdf:rest =

            <PERSON\_LIST@>
            :   - rdf:first = <\_@#a79573306>
                - rdf:rest = <\_@http://www.w3.org/1999/02/22-rdf-syntax-ns#nil>
    - proceedings = <\_@#a32071928>
    - title = 'Ontology Mapping - An Integrated Approach'
    - U.R.L. = 'http://www.aifb.uni-karlsruhe.de/WBS/meh/publications/ehrig04ontology\_ESWS04.pdf'

<Article@a80299267>
:   - author =

      <PERSON\_LIST@>
      :   - rdf:first = <\_@#a29105611>
          - rdf:rest =

            <PERSON\_LIST@>
            :   - rdf:first = <\_@#a98078619>
                - rdf:rest =

                  <PERSON\_LIST@>
                  :   - rdf:first = <\_@#a337716610>
                      - rdf:rest = <\_@http://www.w3.org/1999/02/22-rdf-syntax-ns#nil>
    - journal = <\_@#a246119474>
    - title = 'Start making sense: The Chatty Web approach for global semantic agreements'
    - date =

      <DATE@>
      :   - month = '--12'
          - year = '2003'

<Monograph@a108048723>
:   - publisher = <\_@#a85849488>
    - title = 'Object-Oriented Data Modeling'
    - date =

      <DATE@>
      :   - year = '2000'

<Article@a456080390>
:   - author =

      <PERSON\_LIST@>
      :   - rdf:first = <\_@#a093016135>
          - rdf:rest =

            <PERSON\_LIST@>
            :   - rdf:first = <\_@#a572406328>
                - rdf:rest =

                  <PERSON\_LIST@>
                  :   - rdf:first = <\_@#a086379337>
                      - rdf:rest =

                        <PERSON\_LIST@>
                        :   - rdf:first = <\_@#a04570373>
                            - rdf:rest = <\_@http://www.w3.org/1999/02/22-rdf-syntax-ns#nil>
    - journal = <\_@#a362042121>
    - title = 'On dynamically generated ontology translators in agent communication'
    - pages =

      <PAGE\_RANGE@>
      :   - start\_page = '587'
          - end\_page = '607'
    - date =

      <DATE@>
      :   - month = '--12'
          - year = '2001'

<Article@a846015923>
:   - author =

      <PERSON\_LIST@>
      :   - rdf:first = <\_@#a900366022>
          - rdf:rest =

            <PERSON\_LIST@>
            :   - rdf:first = <\_@#a467748807>
                - rdf:rest =

                  <PERSON\_LIST@>
                  :   - rdf:first = <\_@#a971541439>
                      - rdf:rest =

                        <PERSON\_LIST@>
                        :   - rdf:first = <\_@#a066600210>
                            - rdf:rest = <\_@http://www.w3.org/1999/02/22-rdf-syntax-ns#nil>
    - journal = <\_@#a906774044>
    - title = 'A Framework for Modeling and Evaluating Automatic Semantic Reconciliation'
    - note = 'to appear'
    - date =

      <DATE@>
      :   - year = '2004'

---

Generated by OWL2HTML
