# Supplementary material for: Matching sensor ontologies through siamese neural networks without using reference alignment
Source: PeerJ Comput Sci. 2021 Jun 18;7:e602. doi: 10.7717/peerj-cs.602 (PMC8237319; doi:10.7717/peerj-cs.602)
Supplement: Supplemental Information 1 [file peerj-cs-07-602-s001.zip › 209/onto.html]

# 

Author: Nick Knouf <nknouf@mit.edu>  
Contributor: Antoine Zimmermann <antoine.zimmermann@inrialpes.fr>, Jérôme Euzenat,   
Date: 08/06/2005  
Version: $Id: onto.rdf,v 1.30 2008/05/27 14:41:13 euzenat Exp $

## Classes

**http://www.w3.org/1999/02/22-rdf-syntax-ns#List** (, *)*


**http://xmlns.com/foaf/0.1/Person** (, *)*


**http://xmlns.com/foaf/0.1/Organization** (, *)*


**Entry** (, *)*
:   - #publishingDate [0 1]
    - #heading [0 1]
    - #createdBy [0 1]

    **Book** (, *)*
    :   - #heading [1 1]
        - #volume [0 1]
        - #publishedBy [0 1]
        - #inSeries [0 1]
        - #publishingDate [1 1] *#Date*
        - #writtenBy [1 1]
        - #issue [0 1]

        **Monography** (, *)*
        :   - #sections *#Chapter*

        **Compilation** (, *)*
        :   - #sections *#Chapter*
            - #tomes *#InCompilation*

        **ConferenceMinutes** (, *)*
        :   - #talks *#Communication*
            - #meeting [0 1] *#Congress*
            - #coordinatedBy [0 1]
            - #sponsoredBy [0 1]

    **NonFormal** (, *)*
    :   - #heading [1 1]

        **Brochure** (, *)*


        **CourseMaterial** (, *)*


        **ReferenceGuide** (, *)*
        :   - #sponsoredBy [0 1]
            - #issue [0 1]
            - #heading [1 1]

        **Manuscript** (, *)*
        :   - #writtenBy [1 1]
            - #heading [1 1]
            - #comment [1 +oo]

    **Section** (, *)*
    :   - #pageRange [0 1]
        - #heading [1 1]

        **JournalPaper** (, *)*
        :   - #writtenBy [1 1]
            - #pageRange [1 1]
            - #inPeriodical [1 1]
            - #publishingDate [1 1] *#Date*
            - #number [0 1]
            - #volume [0 1]

        **Chapter** (, *)*
        :   - #section [0 1] [0 1]

        **Extract** (, *)*
        :   - #writtenBy [1 1]
            - #pageRange [1 +oo]
            - #inVolume [1 1]

        **InCompilation** (, *)*
        :   - #writtenBy [1 1]
            - #inCompilation [1 1]

        **Communication** (, *)*
        :   - #writtenBy [1 1]
            - #inMinutes [1 1]

    **StudentReport** (, *)*
    :   - #writtenBy [1 1]
        - #heading [1 1]
        - #issuer [1 1]
        - #publishingDate [1 1]

        **MScThesis** (, *)*


        **DoctoralThesis** (, *)*

    **Various** (, *)*


    **Report** (, *)*
    :   - #writtenBy [1 1]
        - #heading [1 1]
        - #issuedBy [1 1]
        - #publishingDate [1 1] *#Date*
        - #number [0 1]

        **TechnicalReport** (, *)*


        **Deliverable** (, *)*
        :   - #contract [0 1]

    **Movie** (, *)*

**Periodical** (, *)*
:   - #id [1 1] *http://www.w3.org/2001/XMLSchema#string*
    - #acronym *http://www.w3.org/2001/XMLSchema#string*
    - #frequency *http://www.w3.org/2001/XMLSchema#string*
    - #publishedBy [0 1]
    - #inSeries [0 1]
    - #startDate [0 1]
    - #papers *#JournalPaper*

**Congress** (, *)*
:   - #id [1 1]
    - #organizedBy *#Organization*
    - #acronym [0 1]
    - #issue [0 1]
    - #place [0 1]

**Directions** (, *)*
:   - #country [0 1] *http://www.w3.org/2001/XMLSchema#string*
    - #state [0 1] *http://www.w3.org/2001/XMLSchema#string*
    - #town [0 1] *http://www.w3.org/2001/XMLSchema#string*

**Organization** (, *)*
:   super: *http://xmlns.com/foaf/0.1/Organization*  

    - #id [1 1]
    - #acronym [1 1]
    - #coordinates [0 1]

    **PublishingHouse** (, *)*


    **University** (, *)*

**People** (, *)*
:   super: *http://www.w3.org/1999/02/22-rdf-syntax-ns#List*  

    - http://www.w3.org/1999/02/22-rdf-syntax-ns#first [1 1] *http://xmlns.com/foaf/0.1/Person*
    - http://www.w3.org/1999/02/22-rdf-syntax-ns#rest [1 1] (*#People* |  {

      <rdf:List@ttp://www.w3.org/1999/02/22-rdf-syntax-ns#nil>
      } )

**PageInterval** (, *)*
:   - #beginning [1 1]
    - #end [1 1]

**Date** (, *)*
:   - #year [1 1] *http://www.w3.org/2001/XMLSchema#gYear*
    - #month [0 1] *http://www.w3.org/2001/XMLSchema#gMonth*
    - #day [0 1] *http://www.w3.org/2001/XMLSchema#gDay*

## Properties

**http://www.w3.org/1999/02/22-rdf-syntax-ns#first**: http://www.w3.org/1999/02/22-rdf-syntax-ns#List -> \_ *()*


**http://www.w3.org/1999/02/22-rdf-syntax-ns#rest**: http://www.w3.org/1999/02/22-rdf-syntax-ns#List -> http://www.w3.org/1999/02/22-rdf-syntax-ns#List *()*


**sections**: #Entry -> #Chapter *()*


**tomes**: #Entry -> #Section *()*


**talks**: #ConferenceMinutes -> #Communication *()*


**papers**: #Periodical -> #JournalPaper *()*


**coordinates**: http://www.w3.org/2002/07/owl#Thing -> #Directions *()*


**meeting**: #ConferenceMinutes -> #Congress *()*


**organizedBy**: #Congress -> http://xmlns.com/foaf/0.1/Organization *()*


**contract**: #Entry -> http://www.w3.org/2002/07/owl#Thing *()*


**createdBy**: #Entry -> #People *()*
:   **writtenBy**: \_ -> \_ *()*


    **coordinatedBy**: \_ -> \_ *()*


    **directedBy**: #Movie -> \_ *()*

**issuedBy**: #Report -> #Organization *()*


**componentOf**: #Section -> \_ *()*
:   **inPeriodical**: #JournalPaper -> #Periodical *()*


    **inVolume**: #Extract -> #Monography *()*


    **inCompilation**: #InCompilation -> #Compilation *()*


    **inMinutes**: #Communication -> #ConferenceMinutes *()*

**publishingDate**: (*#Entry* | *#Congress*) -> #Date *()*


**sponsoredBy**: (*#ConferenceMinutes* | *#ReferenceGuide*) -> http://xmlns.com/foaf/0.1/Organization *()*


**publishedBy**: (*#Entry* | *#Periodical*) -> #PublishingHouse *()*


**issuer**: (*#StudentReport* | *#CourseMaterial*) -> #University *()*


**place**: (*#Entry* | *#Congress*) -> #Directions *()*


**pageRange**: #Section -> #PageInterval *()*

**http://purl.org/dc/elements/1.1/creator**\_ -> \_ *()*


**http://purl.org/dc/elements/1.1/contributor**\_ -> \_ *()*


**http://purl.org/dc/elements/1.1/description**\_ -> \_ *()*


**http://purl.org/dc/elements/1.1/date**\_ -> \_ *()*


**http://xmlns.com/foaf/0.1/firstName**\_ -> \_ *()*


**lastName**\_ -> \_ *()*


**http://xmlns.com/foaf/0.1/name**\_ -> \_ *()*


**ref** #Entry -> http://www.w3.org/2001/XMLSchema#string *()*


**selection** #Entry -> http://www.w3.org/2001/XMLSchema#string *()*


**annotation** #Entry -> http://www.w3.org/2001/XMLSchema#string *()*


**frequency** #Periodical -> http://www.w3.org/2001/XMLSchema#string *()*


**startDate** #Chapter -> http://www.w3.org/2001/XMLSchema#string *()*


**issue**(*#Book* | *#ReferenceGuide*) -> http://www.w3.org/2001/XMLSchema#string *()*


**PublicationDetails**(*#Various* | *#Brochure*) -> http://www.w3.org/2001/XMLSchema#string *()*


**comment** #Entry -> http://www.w3.org/2001/XMLSchema#string *()*


**inSeries** #Entry -> http://www.w3.org/2001/XMLSchema#string *()*


**heading** #Entry -> http://www.w3.org/2001/XMLSchema#string *()*


**type**(*#Chapter* | *#TechnicalReport* | *#StudentReport*) -> http://www.w3.org/2001/XMLSchema#string *()*


**company** #Entry -> http://www.w3.org/2001/XMLSchema#string *()*


**summary** #Entry -> http://www.w3.org/2001/XMLSchema#string *()*


**table** #Entry -> http://www.w3.org/2001/XMLSchema#string *()*


**rights** #Entry -> http://www.w3.org/2001/XMLSchema#string *()*


**ISBN** #Entry -> http://www.w3.org/2001/XMLSchema#string *()*


**ISSN** #Entry -> http://www.w3.org/2001/XMLSchema#string *()*


**KeyWordsAndPhrases** #Entry -> http://www.w3.org/2001/XMLSchema#string *()*


**idiom** #Entry -> http://www.w3.org/2001/XMLSchema#language *()*


**LCCN** #Entry -> http://www.w3.org/2001/XMLSchema#string *()*


**MRN** #Entry -> http://www.w3.org/2001/XMLSchema#string *()*


**amount** #Entry -> http://www.w3.org/2001/XMLSchema#string *()*


**dimensions** #Entry -> http://www.w3.org/2001/XMLSchema#string *()*


**link** #Entry -> http://www.w3.org/2001/XMLSchema#string *()*


**id**\_ -> http://www.w3.org/2001/XMLSchema#string *()*


**acronym**\_ -> http://www.w3.org/2001/XMLSchema#string *()*


**section** #Section -> http://www.w3.org/2001/XMLSchema#string *()*


**numbering**(*#Entry* | *#Congress*) -> \_ *()*
:   **number** #Entry -> http://www.w3.org/2001/XMLSchema#string *()*


    **issue**(*#Entry* | *#Congress*) -> http://www.w3.org/2001/XMLSchema#string *()*


    **volume** #Entry -> http://www.w3.org/2001/XMLSchema#nonNegativeInteger *()*

**year** #Date -> http://www.w3.org/2001/XMLSchema#gYear *()*


**month** #Date -> http://www.w3.org/2001/XMLSchema#gMonth *()*


**day** #Date -> http://www.w3.org/2001/XMLSchema#gDay *()*


**town** #Directions -> http://www.w3.org/2001/XMLSchema#string *()*


**state** #Directions -> http://www.w3.org/2001/XMLSchema#string *()*


**country** #Directions -> http://www.w3.org/2001/XMLSchema#string *()*


**beginning** #PageInterval -> http://www.w3.org/2001/XMLSchema#nonNegativeInteger *()*


**end** #PageInterval -> http://www.w3.org/2001/XMLSchema#nonNegativeInteger *()*

## Individuals

<rdf:List@ttp://www.w3.org/1999/02/22-rdf-syntax-ns#nil>


<foaf:Person@a04570373>
:   - foaf:name = 'John-Jules Meyer'
    - foaf:firstName = 'John-Jules'
    - lastName = 'Meyer'

<foaf:Person@a43836633>
:   - foaf:name = 'Jeen Broekstra'
    - foaf:firstName = 'Jeen'
    - lastName = 'Broekstra'

<foaf:Person@a85228505>
:   - foaf:name = 'Alexander Mädche'
    - foaf:firstName = 'Alexander'
    - lastName = 'Mädche'

<foaf:Person@a48552212>
:   - foaf:name = 'Björn Schnizler'
    - foaf:firstName = 'Björn'
    - lastName = 'Schnizler'

<foaf:Person@a971541439>
:   - foaf:name = 'Alberto Trombetta'
    - foaf:firstName = 'Alberto'
    - lastName = 'Trombetta'

<foaf:Person@a11090777>
:   - foaf:name = 'Christine Parent'
    - foaf:firstName = 'Christine'
    - lastName = 'Parent'

<foaf:Person@a250331360>
:   - foaf:name = 'R. Schmidt'
    - foaf:firstName = 'R.'
    - lastName = 'Schmidt'

<foaf:Person@a79573306>
:   - foaf:name = 'York Sure'
    - foaf:firstName = 'York'
    - lastName = 'Sure'

<foaf:Person@a885257047>
:   - foaf:name = 'M. Punceva'
    - foaf:firstName = 'M.'
    - lastName = 'Punceva'

<foaf:Person@a74993404>
:   - foaf:name = 'I. V. Levenshtein'
    - foaf:firstName = 'I. V.'
    - lastName = 'Levenshtein'

<foaf:Person@a71003986>
:   - foaf:name = 'Steffen Staab'
    - foaf:firstName = 'Steffen'
    - lastName = 'Staab'

<foaf:Person@a572406328>
:   - foaf:name = 'Frank Boer'
    - foaf:firstName = 'Frank'
    - lastName = 'Boer'

<foaf:Person@a139477786>
:   - foaf:name = 'Maarten Menken'
    - foaf:firstName = 'Maarten'
    - lastName = 'Menken'

<foaf:Person@a337716610>
:   - foaf:name = 'Manfred Hauswirth'
    - foaf:firstName = 'Manfred'
    - lastName = 'Hauswirth'

<foaf:Person@a086379337>
:   - foaf:name = 'Wiebe Hoek'
    - foaf:firstName = 'Wiebe'
    - lastName = 'Hoek'

<foaf:Person@a712561038>
:   - foaf:name = 'Marc Ehrig'
    - foaf:firstName = 'Marc'
    - lastName = 'Ehrig'

<foaf:Person@a066600210>
:   - foaf:name = 'Danilo Montesi'
    - foaf:firstName = 'Danilo'
    - lastName = 'Montesi'

<foaf:Person@a093016135>
:   - foaf:name = 'Rogier Eijk'
    - foaf:firstName = 'Rogier'
    - lastName = 'Eijk'

<foaf:Person@a944339054>
:   - foaf:name = 'Frank van Harmelen'
    - foaf:firstName = 'Frank'
    - lastName = 'van Harmelen'

<foaf:Person@a98078619>
:   - foaf:name = 'Philippe Cudré-Mauroux'
    - foaf:firstName = 'Philippe'
    - lastName = 'Cudré-Mauroux'

<foaf:Person@a39510672>
:   - foaf:name = 'Z. Despotovic'
    - foaf:firstName = 'Z.'
    - lastName = 'Despotovic'

<foaf:Person@a431956276>
:   - foaf:name = 'Stefano Spaccapietra'
    - foaf:firstName = 'Stefano'
    - lastName = 'Spaccapietra'

<foaf:Person@a431956276b>
:   - foaf:name = 'Mike Papazoglou'
    - foaf:firstName = 'Mike'
    - lastName = 'Papazoglou'

<foaf:Person@a431956276c>
:   - foaf:name = 'Zahir Tari'
    - foaf:firstName = 'Zahir'
    - lastName = 'Tari'

<foaf:Person@a70955601>
:   - foaf:name = 'A. Datta'
    - foaf:firstName = 'A.'
    - lastName = 'Datta'

<foaf:Person@a467748807>
:   - foaf:name = 'Ateret Anaby-Tavor'
    - foaf:firstName = 'Ateret'
    - lastName = 'Anaby-Tavor'

<foaf:Person@a3105947>
:   - foaf:name = 'Ronny Siebes'
    - foaf:firstName = 'Ronny'
    - lastName = 'Siebes'

<foaf:Person@a29105611>
:   - foaf:name = 'Karl Aberer'
    - foaf:firstName = 'Karl'
    - lastName = 'Aberer'

<foaf:Person@a958684218>
:   - foaf:name = 'Peter Mika'
    - foaf:firstName = 'Peter'
    - lastName = 'Mika'

<foaf:Person@a94533498>
:   - foaf:name = 'Peter Haase'
    - foaf:firstName = 'Peter'
    - lastName = 'Haase'

<foaf:Person@a900366022>
:   - foaf:name = 'Avigdor Gal'
    - foaf:firstName = 'Avigdor'
    - lastName = 'Gal'

<Periodical@a246119474>
:   - foaf:name = 'Journal of Web Semantics'
    - acronym = 'JWS'

<PublishingHouse@a131020767>
:   - id = 'Springer-Verlag'
    - coordinates =

      <Directions@>
      :   - town = 'Heidelberg'
          - country = 'DE'

<Periodical@a70981683>
:   - id = 'Cybernetics and Control Theory'

<PublishingHouse@a85849488>
:   - id = 'The MIT Press'
    - coordinates =

      <Directions@>
      :   - town = 'Cambridge'
          - state = 'MA'
          - country = 'US'

<Periodical@a362042121>
:   - id = 'International journal of intelligent system'
    - acronym = 'IJIS'

<Periodical@a674639524>
:   - id = 'ACM SIGMOD Record'

<Periodical@a906774044>
:   - id = 'VLDB Journal'

<Congress@spg04>
:   - id = 'SemPGrid 04 Workshop'
    - place =

      <Directions@>
      :   - town = 'New-York'
          - state = 'NY'
          - country = 'US'
    - publishingDate =

      <Date@>
      :   - month = '--05'
          - year = '2004'

<Congress@a72192307c>
:   - id = 'Int. Conference on Knowledge Engineering and Management'
    - acronym = 'EKAW'
    - issue = '13'
    - publishingDate =

      <Date@>
      :   - month = '--10'
          - year = '2002'

<Congress@a32071928c>
:   - id = 'European Semantic Web Symposium'
    - acronym = 'ESWS'
    - issue = '1'
    - place =

      <Directions@>
      :   - town = 'Heraklion'
          - country = 'GR'
    - publishingDate =

      <Date@>
      :   - month = '--05'
          - year = '2004'

<ConferenceMinutes@a060097576>
:   - heading = 'Proceedings of the SemPGrid 04 Workshop'
    - publishingDate =

      <Date@>
      :   - year = '2004'
    - meeting = <\_@#spg04>

<Communication@a64263824>
:   - writtenBy =

      <People@>
      :   - rdf:first = <\_@#a43836633>
          - rdf:rest =

            <People@>
            :   - rdf:first = <\_@#a712561038>
                - rdf:rest =

                  <People@>
                  :   - rdf:first = <\_@#a94533498>
                      - rdf:rest =

                        <People@>
                        :   - rdf:first = <\_@#a944339054>
                            - rdf:rest =

                              <People@>
                              :   - rdf:first = <\_@#a139477786>
                                  - rdf:rest =

                                    <People@>
                                    :   - rdf:first = <\_@#a958684218>
                                        - rdf:rest =

                                          <People@>
                                          :   - rdf:first = <\_@#a48552212>
                                              - rdf:rest =

                                                <People@>
                                                :   - rdf:first = <\_@#a3105947>
                                                    - rdf:rest = <\_@http://www.w3.org/1999/02/22-rdf-syntax-ns#nil>
    - inMinutes = <\_@#a060097576>
    - heading = 'Bibster - A Semantics-Based Bibliographic Peer-to-Peer System'

<Communication@a439508789>
:   - writtenBy =

      <People@>
      :   - rdf:first = <\_@#a85228505>
          - rdf:rest =

            <People@>
            :   - rdf:first = <\_@#a71003986>
                - rdf:rest = <\_@http://www.w3.org/1999/02/22-rdf-syntax-ns#nil>
    - inMinutes = <\_@#a72192307>
    - heading = 'Measuring Similarity between Ontologies'

<JournalPaper@a492378321>
:   - writtenBy =

      <People@>
      :   - rdf:first = <\_@#a29105611>
          - rdf:rest =

            <People@>
            :   - rdf:first = <\_@#a98078619>
                - rdf:rest =

                  <People@>
                  :   - rdf:first = <\_@#a70955601>
                      - rdf:rest =

                        <People@>
                        :   - rdf:first = <\_@#a39510672>
                            - rdf:rest =

                              <People@>
                              :   - rdf:first = <\_@#a337716610>
                                  - rdf:rest =

                                    <People@>
                                    :   - rdf:first = <\_@#a885257047>
                                        - rdf:rest =

                                          <People@>
                                          :   - rdf:first = <\_@#a250331360>
                                              - rdf:rest = <\_@http://www.w3.org/1999/02/22-rdf-syntax-ns#nil>
    - inPeriodical = <\_@#a674639524>
    - heading = '{P-Grid}: A Self-organizing Structured P2P System'
    - publishingDate =

      <Date@>
      :   - year = '2003'

<JournalPaper@a475526642>
:   - writtenBy =

      <People@>
      :   - rdf:first = <\_@#a74993404>
          - rdf:rest = <\_@http://www.w3.org/1999/02/22-rdf-syntax-ns#nil>
    - inPeriodical = <\_@#a70981683>
    - heading = 'Binary Codes capable of correcting deletions, insertions, and reversals'
    - publishingDate =

      <Date@>
      :   - year = '1996'

<Extract@a71568377>
:   - writtenBy =

      <People@>
      :   - rdf:first = <\_@#a11090777>
          - rdf:rest =

            <People@>
            :   - rdf:first = <\_@#a431956276>
                - rdf:rest = <\_@http://www.w3.org/1999/02/22-rdf-syntax-ns#nil>
    - inVolume = <\_@#a108048723>
    - heading = 'Database integration: the key to data interoperability'
    - coordinatedBy =

      <People@>
      :   - rdf:first = <\_@#a431956276>
          - rdf:rest =

            <People@>
            :   - rdf:first = <\_@#a431956276b>
                - rdf:rest =

                  <People@>
                  :   - rdf:first = <\_@#a431956276c>
                      - rdf:rest = <\_@http://www.w3.org/1999/02/22-rdf-syntax-ns#nil>

<ConferenceMinutes@a72192307>
:   - publishedBy = <\_@#a131020767>
    - heading = 'Proc. Of the 13th Int. Conference on Knowledge Engineering and Management (EKAW-2002)'
    - meeting = <\_@#a72192307c>
    - publishingDate =

      <Date@>
      :   - year = '2002'

<ConferenceMinutes@a32071928>
:   - publishedBy = <\_@#a131020767>
    - meeting = <\_@#a32071928c>
    - heading = 'Proceedings of the First European Semantic Web Symposium'
    - publishingDate =

      <Date@>
      :   - year = '2004'

<Various@a140583454>
:   - writtenBy =

      <People@>
      :   - rdf:first = <\_@#a712561038>
          - rdf:rest =

            <People@>
            :   - rdf:first = <\_@#a71003986>
                - rdf:rest = <\_@http://www.w3.org/1999/02/22-rdf-syntax-ns#nil>
    - heading = '{QOM} - Quick Ontology Mapping'
    - comment = 'submitted to the ISWC 04'
    - publishingDate =

      <Date@>
      :   - year = '2004'

<Communication@a11065952>
:   - writtenBy =

      <People@>
      :   - rdf:first = <\_@#a712561038>
          - rdf:rest =

            <People@>
            :   - rdf:first = <\_@#a79573306>
                - rdf:rest = <\_@http://www.w3.org/1999/02/22-rdf-syntax-ns#nil>
    - inMinutes = <\_@#a32071928>
    - heading = 'Ontology Mapping - An Integrated Approach'
    - link = 'http://www.aifb.uni-karlsruhe.de/WBS/meh/publications/ehrig04ontology\_ESWS04.pdf'

<JournalPaper@a80299267>
:   - writtenBy =

      <People@>
      :   - rdf:first = <\_@#a29105611>
          - rdf:rest =

            <People@>
            :   - rdf:first = <\_@#a98078619>
                - rdf:rest =

                  <People@>
                  :   - rdf:first = <\_@#a337716610>
                      - rdf:rest = <\_@http://www.w3.org/1999/02/22-rdf-syntax-ns#nil>
    - inPeriodical = <\_@#a246119474>
    - heading = 'Start making sense: The Chatty Web approach for global semantic agreements'
    - publishingDate =

      <Date@>
      :   - month = '--12'
          - year = '2003'

<Monography@a108048723>
:   - publishedBy = <\_@#a85849488>
    - heading = 'Object-Oriented Data Modeling'
    - publishingDate =

      <Date@>
      :   - year = '2000'

<JournalPaper@a456080390>
:   - writtenBy =

      <People@>
      :   - rdf:first = <\_@#a093016135>
          - rdf:rest =

            <People@>
            :   - rdf:first = <\_@#a572406328>
                - rdf:rest =

                  <People@>
                  :   - rdf:first = <\_@#a086379337>
                      - rdf:rest =

                        <People@>
                        :   - rdf:first = <\_@#a04570373>
                            - rdf:rest = <\_@http://www.w3.org/1999/02/22-rdf-syntax-ns#nil>
    - inPeriodical = <\_@#a362042121>
    - heading = 'On dynamically generated ontology translators in agent communication'
    - pageRange =

      <PageInterval@>
      :   - beginning = '587'
          - end = '607'
    - publishingDate =

      <Date@>
      :   - month = '--12'
          - year = '2001'

<JournalPaper@a846015923>
:   - writtenBy =

      <People@>
      :   - rdf:first = <\_@#a900366022>
          - rdf:rest =

            <People@>
            :   - rdf:first = <\_@#a467748807>
                - rdf:rest =

                  <People@>
                  :   - rdf:first = <\_@#a971541439>
                      - rdf:rest =

                        <People@>
                        :   - rdf:first = <\_@#a066600210>
                            - rdf:rest = <\_@http://www.w3.org/1999/02/22-rdf-syntax-ns#nil>
    - inPeriodical = <\_@#a906774044>
    - heading = 'A Framework for Modeling and Evaluating Automatic Semantic Reconciliation'
    - comment = 'to appear'
    - publishingDate =

      <Date@>
      :   - year = '2004'

---

Generated by OWL2HTML
