# Supplementary material for: Matching sensor ontologies through siamese neural networks without using reference alignment
Source: PeerJ Comput Sci. 2021 Jun 18;7:e602. doi: 10.7717/peerj-cs.602 (PMC8237319; doi:10.7717/peerj-cs.602)
Supplement: Supplemental Information 1 [file peerj-cs-07-602-s001.zip › 209/refalign.html]

# (level 0) Alignment

## Source: http://oaei.ontologymatching.org/2011/benchmarks/101/onto.rdf

## Target: http://oaei.ontologymatching.org/2011/benchmarks/209/onto.rdf

## Correspondences

type = type
:   1.0

howPublished = PublicationDetails
:   1.0

periodicity = frequency
:   1.0

proceedings = inMinutes
:   1.0

volume = volume
:   1.0

annote = annotation
:   1.0

PersonList = People
:   1.0

month = month
:   1.0

copyright = rights
:   1.0

Unpublished = Manuscript
:   1.0

address = coordinates
:   1.0

Address = Directions
:   1.0

chapter = section
:   1.0

Chapter = Chapter
:   1.0

editor = coordinatedBy
:   1.0

InBook = Extract
:   1.0

Date = Date
:   1.0

series = inSeries
:   1.0

PageRange = PageInterval
:   1.0

date = publishingDate
:   1.0

title = heading
:   1.0

Booklet = Brochure
:   1.0

numberOrVolume = numbering
:   1.0

LectureNotes = CourseMaterial
:   1.0

url = link
:   1.0

MastersThesis = MScThesis
:   1.0

organizer = organizedBy
:   1.0

mrNumber = MRN
:   1.0

pages = pageRange
:   1.0

TechReport = TechnicalReport
:   1.0

reviewed = selection
:   1.0

startPage = beginning
:   1.0

edition = issue
:   1.0

lccn = LCCN
:   1.0

affiliation = company
:   1.0

institution = issuedBy
:   1.0

year = year
:   1.0

isPartOf = componentOf
:   1.0

organization = sponsoredBy
:   1.0

country = country
:   1.0

publisher = publishedBy
:   1.0

school = issuer
:   1.0

Misc = Various
:   1.0

collection = inCompilation
:   1.0

Collection = Compilation
:   1.0

isbn = ISBN
:   1.0

abstract = summary
:   1.0

directors = directedBy
:   1.0

Academic = StudentReport
:   1.0

location = place
:   1.0

MotionPicture = Movie
:   1.0

Article = JournalPaper
:   1.0

Informal = NonFormal
:   1.0

price = amount
:   1.0

name = id
:   1.0

event = meeting
:   1.0

state = state
:   1.0

Book = Book
:   1.0

book = inVolume
:   1.0

day = day
:   1.0

School = University
:   1.0

shortName = acronym
:   1.0

PhdThesis = DoctoralThesis
:   1.0

Proceedings = ConferenceMinutes
:   1.0

number = number
:   1.0

issue = issue
:   1.0

Reference = Entry
:   1.0

endPage = end
:   1.0

InCollection = InCompilation
:   1.0

firstPublished = startDate
:   1.0

author = writtenBy
:   1.0

Report = Report
:   1.0

note = comment
:   1.0

humanCreator = createdBy
:   1.0

Conference = Congress
:   1.0

Part = Section
:   1.0

Publisher = PublishingHouse
:   1.0

contract = contract
:   1.0

Manual = ReferenceGuide
:   1.0

key = ref
:   1.0

InProceedings = Communication
:   1.0

Journal = Periodical
:   1.0

journal = inPeriodical
:   1.0

Monograph = Monography
:   1.0

keywords = KeyWordsAndPhrases
:   1.0

issn = ISSN
:   1.0

contents = table
:   1.0

city = town
:   1.0

Institution = Organization
:   1.0

Deliverable = Deliverable
:   1.0

size = dimensions
:   1.0

chapters = sections
:   1.0

parts = tomes
:   1.0

communications = talks
:   1.0

articles = papers
:   1.0

lastName = lastName
:   1.0

language = idiom
:   1.0
