# Supplementary material for: Matching sensor ontologies through siamese neural networks without using reference alignment
Source: PeerJ Comput Sci. 2021 Jun 18;7:e602. doi: 10.7717/peerj-cs.602 (PMC8237319; doi:10.7717/peerj-cs.602)
Supplement: Supplemental Information 1 [file peerj-cs-07-602-s001.zip › 210/onto.html]

# 

Author: Nick Knouf <nknouf@mit.edu>  
Contributor: Antoine Zimmermann <antoine.zimmermann@inrialpes.fr>, Jérôme Euzenat,   
Date: 08/06/2005  
Version: $Id: onto.rdf,v 1.30 2008/05/27 14:41:13 euzenat Exp $

## Classes

**http://www.w3.org/1999/02/22-rdf-syntax-ns#List** (, *)*


**http://xmlns.com/foaf/0.1/Person** (, *)*


**http://xmlns.com/foaf/0.1/Organization** (, *)*


**Référence** (, *)*
:   - #date [0 1]
    - #titre [0 1]
    - #créateur [0 1]

    **Livre** (, *)*
    :   - #titre [1 1]
        - #volume [0 1]
        - #éditeur [0 1]
        - #série [0 1]
        - #date [1 1] *#Date*
        - #auteurs [1 1]
        - #édition [0 1]

        **Monographie** (, *)*
        :   - #chapitres *#Chapitre*

        **Compilation** (, *)*
        :   - #chapitres *#Chapitre*
            - #parties *#ExtraitCompilation*

        **Actes** (, *)*
        :   - #communications *#ExtraitActes*
            - #évènement [0 1] *#Conférence*
            - #éditeurs [0 1]
            - #organisation [0 1]

    **Informel** (, *)*
    :   - #titre [1 1]

        **Livret** (, *)*


        **Polycopié** (, *)*


        **Manuel** (, *)*
        :   - #organisation [0 1]
            - #édition [0 1]
            - #titre [1 1]

        **NonPublié** (, *)*
        :   - #auteurs [1 1]
            - #titre [1 1]
            - #note [1 +oo]

    **Partie** (, *)*
    :   - #pages [0 1]
        - #titre [1 1]

        **Article** (, *)*
        :   - #auteurs [1 1]
            - #pages [1 1]
            - #journal [1 1]
            - #date [1 1] *#Date*
            - #numéro [0 1]
            - #volume [0 1]

        **Chapitre** (, *)*
        :   - #chapitre [0 1] [0 1]

        **ExtraitLivre** (, *)*
        :   - #auteurs [1 1]
            - #pages [1 +oo]
            - #livre [1 1]

        **ExtraitCompilation** (, *)*
        :   - #auteurs [1 1]
            - #collection [1 1]

        **ExtraitActes** (, *)*
        :   - #auteurs [1 1]
            - #actes [1 1]

    **Mémoire** (, *)*
    :   - #auteurs [1 1]
        - #titre [1 1]
        - #institution [1 1]
        - #date [1 1]

        **MémoireDeMastère** (, *)*


        **MémoireDeDoctorat** (, *)*

    **Divers** (, *)*


    **Raport** (, *)*
    :   - #auteurs [1 1]
        - #titre [1 1]
        - #institution [1 1]
        - #date [1 1] *#Date*
        - #numéro [0 1]

        **RaportTechnique** (, *)*


        **Livrable** (, *)*
        :   - #contrat [0 1]

    **Film** (, *)*

**Revue** (, *)*
:   - #nom [1 1] *http://www.w3.org/2001/XMLSchema#string*
    - #nomCourt *http://www.w3.org/2001/XMLSchema#string*
    - #périodicité *http://www.w3.org/2001/XMLSchema#string*
    - #éditeur [0 1]
    - #série [0 1]
    - #dateDePublication [0 1]
    - #articles *#Article*

**Conférence** (, *)*
:   - #nom [1 1]
    - #organisateur *#Institution*
    - #nomCourt [0 1]
    - #sortie [0 1]
    - #localisation [0 1]

**Adresse** (, *)*
:   - #pays [0 1] *http://www.w3.org/2001/XMLSchema#string*
    - #état [0 1] *http://www.w3.org/2001/XMLSchema#string*
    - #ville [0 1] *http://www.w3.org/2001/XMLSchema#string*

**Institution** (, *)*
:   super: *http://xmlns.com/foaf/0.1/Organization*  

    - #nom [1 1]
    - #nomCourt [1 1]
    - #adresse [0 1]

    **Éditeur** (, *)*


    **Université** (, *)*

**Personnes** (, *)*
:   super: *http://www.w3.org/1999/02/22-rdf-syntax-ns#List*  

    - http://www.w3.org/1999/02/22-rdf-syntax-ns#first [1 1] *http://xmlns.com/foaf/0.1/Person*
    - http://www.w3.org/1999/02/22-rdf-syntax-ns#rest [1 1] (*#Personnes* |  {

      <rdf:List@ttp://www.w3.org/1999/02/22-rdf-syntax-ns#nil>
      } )

**IntervalleDePages** (, *)*
:   - #pageDébut [1 1]
    - #pageFin [1 1]

**Date** (, *)*
:   - #année [1 1] *http://www.w3.org/2001/XMLSchema#gYear*
    - #mois [0 1] *http://www.w3.org/2001/XMLSchema#gMonth*
    - #jour [0 1] *http://www.w3.org/2001/XMLSchema#gDay*

## Properties

**http://www.w3.org/1999/02/22-rdf-syntax-ns#first**: http://www.w3.org/1999/02/22-rdf-syntax-ns#List -> \_ *()*


**http://www.w3.org/1999/02/22-rdf-syntax-ns#rest**: http://www.w3.org/1999/02/22-rdf-syntax-ns#List -> http://www.w3.org/1999/02/22-rdf-syntax-ns#List *()*


**chapitres**: #Référence -> #Chapitre *()*


**parties**: #Référence -> #Partie *()*


**communications**: #Actes -> #ExtraitActes *()*


**articles**: #Revue -> #Article *()*


**adresse**: http://www.w3.org/2002/07/owl#Thing -> #Adresse *()*


**évènement**: #Actes -> #Conférence *()*


**organisateur**: #Conférence -> http://xmlns.com/foaf/0.1/Organization *()*


**contrat**: #Référence -> http://www.w3.org/2002/07/owl#Thing *()*


**créateur**: #Référence -> #Personnes *()*
:   **auteurs**: \_ -> \_ *()*


    **éditeurs**: \_ -> \_ *()*


    **réalisateurs**: #Film -> \_ *()*

**institution**: #Raport -> #Institution *()*


**partieDe**: #Partie -> \_ *()*
:   **journal**: #Article -> #Revue *()*


    **livre**: #ExtraitLivre -> #Monographie *()*


    **collection**: #ExtraitCompilation -> #Compilation *()*


    **actes**: #ExtraitActes -> #Actes *()*

**date**: (*#Référence* | *#Conférence*) -> #Date *()*


**organisation**: (*#Actes* | *#Manuel*) -> http://xmlns.com/foaf/0.1/Organization *()*


**éditeur**: (*#Référence* | *#Revue*) -> #Éditeur *()*


**institution**: (*#Mémoire* | *#Polycopié*) -> #Université *()*


**localisation**: (*#Référence* | *#Conférence*) -> #Adresse *()*


**pages**: #Partie -> #IntervalleDePages *()*

**http://purl.org/dc/elements/1.1/creator**\_ -> \_ *()*


**http://purl.org/dc/elements/1.1/contributor**\_ -> \_ *()*


**http://purl.org/dc/elements/1.1/description**\_ -> \_ *()*


**http://purl.org/dc/elements/1.1/date**\_ -> \_ *()*


**http://xmlns.com/foaf/0.1/firstName**\_ -> \_ *()*


**lastName**\_ -> \_ *()*


**http://xmlns.com/foaf/0.1/name**\_ -> \_ *()*


**Clé** #Référence -> http://www.w3.org/2001/XMLSchema#string *()*


**Évalué** #Référence -> http://www.w3.org/2001/XMLSchema#string *()*


**annote** #Référence -> http://www.w3.org/2001/XMLSchema#string *()*


**périodicité** #Revue -> http://www.w3.org/2001/XMLSchema#string *()*


**dateDePublication** #Chapitre -> http://www.w3.org/2001/XMLSchema#string *()*


**édition**(*#Livre* | *#Manuel*) -> http://www.w3.org/2001/XMLSchema#string *()*


**modeDePublication**(*#Divers* | *#Livret*) -> http://www.w3.org/2001/XMLSchema#string *()*


**note** #Référence -> http://www.w3.org/2001/XMLSchema#string *()*


**série** #Référence -> http://www.w3.org/2001/XMLSchema#string *()*


**titre** #Référence -> http://www.w3.org/2001/XMLSchema#string *()*


**catégorie**(*#Chapitre* | *#RaportTechnique* | *#Mémoire*) -> http://www.w3.org/2001/XMLSchema#string *()*


**affiliation** #Référence -> http://www.w3.org/2001/XMLSchema#string *()*


**résumé** #Référence -> http://www.w3.org/2001/XMLSchema#string *()*


**contenu** #Référence -> http://www.w3.org/2001/XMLSchema#string *()*


**droits** #Référence -> http://www.w3.org/2001/XMLSchema#string *()*


**isbn** #Référence -> http://www.w3.org/2001/XMLSchema#string *()*


**issn** #Référence -> http://www.w3.org/2001/XMLSchema#string *()*


**mots-clé** #Référence -> http://www.w3.org/2001/XMLSchema#string *()*


**langage** #Référence -> http://www.w3.org/2001/XMLSchema#language *()*


**lccn** #Référence -> http://www.w3.org/2001/XMLSchema#string *()*


**noMR** #Référence -> http://www.w3.org/2001/XMLSchema#string *()*


**prix** #Référence -> http://www.w3.org/2001/XMLSchema#string *()*


**taille** #Référence -> http://www.w3.org/2001/XMLSchema#string *()*


**url** #Référence -> http://www.w3.org/2001/XMLSchema#string *()*


**nom**\_ -> http://www.w3.org/2001/XMLSchema#string *()*


**nomCourt**\_ -> http://www.w3.org/2001/XMLSchema#string *()*


**chapitre** #Partie -> http://www.w3.org/2001/XMLSchema#string *()*


**numéroOuVolume**(*#Référence* | *#Conférence*) -> \_ *()*
:   **numéro** #Référence -> http://www.w3.org/2001/XMLSchema#string *()*


    **sortie**(*#Référence* | *#Conférence*) -> http://www.w3.org/2001/XMLSchema#string *()*


    **volume** #Référence -> http://www.w3.org/2001/XMLSchema#nonNegativeInteger *()*

**année** #Date -> http://www.w3.org/2001/XMLSchema#gYear *()*


**mois** #Date -> http://www.w3.org/2001/XMLSchema#gMonth *()*


**jour** #Date -> http://www.w3.org/2001/XMLSchema#gDay *()*


**ville** #Adresse -> http://www.w3.org/2001/XMLSchema#string *()*


**état** #Adresse -> http://www.w3.org/2001/XMLSchema#string *()*


**pays** #Adresse -> http://www.w3.org/2001/XMLSchema#string *()*


**pageDébut** #IntervalleDePages -> http://www.w3.org/2001/XMLSchema#nonNegativeInteger *()*


**pageFin** #IntervalleDePages -> http://www.w3.org/2001/XMLSchema#nonNegativeInteger *()*

## Individuals

<rdf:List@ttp://www.w3.org/1999/02/22-rdf-syntax-ns#nil>


<foaf:Person@a04570373>
:   - foaf:name = 'John-Jules Meyer'
    - foaf:firstName = 'John-Jules'
    - lastName = 'Meyer'

<foaf:Person@a43836633>
:   - foaf:name = 'Jeen Broekstra'
    - foaf:firstName = 'Jeen'
    - lastName = 'Broekstra'

<foaf:Person@a85228505>
:   - foaf:name = 'Alexander Mädche'
    - foaf:firstName = 'Alexander'
    - lastName = 'Mädche'

<foaf:Person@a48552212>
:   - foaf:name = 'Björn Schnizler'
    - foaf:firstName = 'Björn'
    - lastName = 'Schnizler'

<foaf:Person@a971541439>
:   - foaf:name = 'Alberto Trombetta'
    - foaf:firstName = 'Alberto'
    - lastName = 'Trombetta'

<foaf:Person@a11090777>
:   - foaf:name = 'Christine Parent'
    - foaf:firstName = 'Christine'
    - lastName = 'Parent'

<foaf:Person@a250331360>
:   - foaf:name = 'R. Schmidt'
    - foaf:firstName = 'R.'
    - lastName = 'Schmidt'

<foaf:Person@a79573306>
:   - foaf:name = 'York Sure'
    - foaf:firstName = 'York'
    - lastName = 'Sure'

<foaf:Person@a885257047>
:   - foaf:name = 'M. Punceva'
    - foaf:firstName = 'M.'
    - lastName = 'Punceva'

<foaf:Person@a74993404>
:   - foaf:name = 'I. V. Levenshtein'
    - foaf:firstName = 'I. V.'
    - lastName = 'Levenshtein'

<foaf:Person@a71003986>
:   - foaf:name = 'Steffen Staab'
    - foaf:firstName = 'Steffen'
    - lastName = 'Staab'

<foaf:Person@a572406328>
:   - foaf:name = 'Frank Boer'
    - foaf:firstName = 'Frank'
    - lastName = 'Boer'

<foaf:Person@a139477786>
:   - foaf:name = 'Maarten Menken'
    - foaf:firstName = 'Maarten'
    - lastName = 'Menken'

<foaf:Person@a337716610>
:   - foaf:name = 'Manfred Hauswirth'
    - foaf:firstName = 'Manfred'
    - lastName = 'Hauswirth'

<foaf:Person@a086379337>
:   - foaf:name = 'Wiebe Hoek'
    - foaf:firstName = 'Wiebe'
    - lastName = 'Hoek'

<foaf:Person@a712561038>
:   - foaf:name = 'Marc Ehrig'
    - foaf:firstName = 'Marc'
    - lastName = 'Ehrig'

<foaf:Person@a066600210>
:   - foaf:name = 'Danilo Montesi'
    - foaf:firstName = 'Danilo'
    - lastName = 'Montesi'

<foaf:Person@a093016135>
:   - foaf:name = 'Rogier Eijk'
    - foaf:firstName = 'Rogier'
    - lastName = 'Eijk'

<foaf:Person@a944339054>
:   - foaf:name = 'Frank van Harmelen'
    - foaf:firstName = 'Frank'
    - lastName = 'van Harmelen'

<foaf:Person@a98078619>
:   - foaf:name = 'Philippe Cudré-Mauroux'
    - foaf:firstName = 'Philippe'
    - lastName = 'Cudré-Mauroux'

<foaf:Person@a39510672>
:   - foaf:name = 'Z. Despotovic'
    - foaf:firstName = 'Z.'
    - lastName = 'Despotovic'

<foaf:Person@a431956276>
:   - foaf:name = 'Stefano Spaccapietra'
    - foaf:firstName = 'Stefano'
    - lastName = 'Spaccapietra'

<foaf:Person@a431956276b>
:   - foaf:name = 'Mike Papazoglou'
    - foaf:firstName = 'Mike'
    - lastName = 'Papazoglou'

<foaf:Person@a431956276c>
:   - foaf:name = 'Zahir Tari'
    - foaf:firstName = 'Zahir'
    - lastName = 'Tari'

<foaf:Person@a70955601>
:   - foaf:name = 'A. Datta'
    - foaf:firstName = 'A.'
    - lastName = 'Datta'

<foaf:Person@a467748807>
:   - foaf:name = 'Ateret Anaby-Tavor'
    - foaf:firstName = 'Ateret'
    - lastName = 'Anaby-Tavor'

<foaf:Person@a3105947>
:   - foaf:name = 'Ronny Siebes'
    - foaf:firstName = 'Ronny'
    - lastName = 'Siebes'

<foaf:Person@a29105611>
:   - foaf:name = 'Karl Aberer'
    - foaf:firstName = 'Karl'
    - lastName = 'Aberer'

<foaf:Person@a958684218>
:   - foaf:name = 'Peter Mika'
    - foaf:firstName = 'Peter'
    - lastName = 'Mika'

<foaf:Person@a94533498>
:   - foaf:name = 'Peter Haase'
    - foaf:firstName = 'Peter'
    - lastName = 'Haase'

<foaf:Person@a900366022>
:   - foaf:name = 'Avigdor Gal'
    - foaf:firstName = 'Avigdor'
    - lastName = 'Gal'

<Revue@a246119474>
:   - foaf:name = 'Journal of Web Semantics'
    - nomCourt = 'JWS'

<Éditeur@a131020767>
:   - nom = 'Springer-Verlag'
    - adresse =

      <Adresse@>
      :   - ville = 'Heidelberg'
          - pays = 'DE'

<Revue@a70981683>
:   - nom = 'Cybernetics and Control Theory'

<Éditeur@a85849488>
:   - nom = 'The MIT Press'
    - adresse =

      <Adresse@>
      :   - ville = 'Cambridge'
          - état = 'MA'
          - pays = 'US'

<Revue@a362042121>
:   - nom = 'International journal of intelligent system'
    - nomCourt = 'IJIS'

<Revue@a674639524>
:   - nom = 'ACM SIGMOD Record'

<Revue@a906774044>
:   - nom = 'VLDB Journal'

<Conférence@spg04>
:   - nom = 'SemPGrid 04 Workshop'
    - localisation =

      <Adresse@>
      :   - ville = 'New-York'
          - état = 'NY'
          - pays = 'US'
    - date =

      <Date@>
      :   - mois = '--05'
          - année = '2004'

<Conférence@a72192307c>
:   - nom = 'Int. Conference on Knowledge Engineering and Management'
    - nomCourt = 'EKAW'
    - sortie = '13'
    - date =

      <Date@>
      :   - mois = '--10'
          - année = '2002'

<Conférence@a32071928c>
:   - nom = 'European Semantic Web Symposium'
    - nomCourt = 'ESWS'
    - sortie = '1'
    - localisation =

      <Adresse@>
      :   - ville = 'Heraklion'
          - pays = 'GR'
    - date =

      <Date@>
      :   - mois = '--05'
          - année = '2004'

<Actes@a060097576>
:   - titre = 'Proceedings of the SemPGrid 04 Workshop'
    - date =

      <Date@>
      :   - année = '2004'
    - évènement = <\_@#spg04>

<ExtraitActes@a64263824>
:   - auteurs =

      <Personnes@>
      :   - rdf:first = <\_@#a43836633>
          - rdf:rest =

            <Personnes@>
            :   - rdf:first = <\_@#a712561038>
                - rdf:rest =

                  <Personnes@>
                  :   - rdf:first = <\_@#a94533498>
                      - rdf:rest =

                        <Personnes@>
                        :   - rdf:first = <\_@#a944339054>
                            - rdf:rest =

                              <Personnes@>
                              :   - rdf:first = <\_@#a139477786>
                                  - rdf:rest =

                                    <Personnes@>
                                    :   - rdf:first = <\_@#a958684218>
                                        - rdf:rest =

                                          <Personnes@>
                                          :   - rdf:first = <\_@#a48552212>
                                              - rdf:rest =

                                                <Personnes@>
                                                :   - rdf:first = <\_@#a3105947>
                                                    - rdf:rest = <\_@http://www.w3.org/1999/02/22-rdf-syntax-ns#nil>
    - actes = <\_@#a060097576>
    - titre = 'Bibster - A Semantics-Based Bibliographic Peer-to-Peer System'

<ExtraitActes@a439508789>
:   - auteurs =

      <Personnes@>
      :   - rdf:first = <\_@#a85228505>
          - rdf:rest =

            <Personnes@>
            :   - rdf:first = <\_@#a71003986>
                - rdf:rest = <\_@http://www.w3.org/1999/02/22-rdf-syntax-ns#nil>
    - actes = <\_@#a72192307>
    - titre = 'Measuring Similarity between Ontologies'

<Article@a492378321>
:   - auteurs =

      <Personnes@>
      :   - rdf:first = <\_@#a29105611>
          - rdf:rest =

            <Personnes@>
            :   - rdf:first = <\_@#a98078619>
                - rdf:rest =

                  <Personnes@>
                  :   - rdf:first = <\_@#a70955601>
                      - rdf:rest =

                        <Personnes@>
                        :   - rdf:first = <\_@#a39510672>
                            - rdf:rest =

                              <Personnes@>
                              :   - rdf:first = <\_@#a337716610>
                                  - rdf:rest =

                                    <Personnes@>
                                    :   - rdf:first = <\_@#a885257047>
                                        - rdf:rest =

                                          <Personnes@>
                                          :   - rdf:first = <\_@#a250331360>
                                              - rdf:rest = <\_@http://www.w3.org/1999/02/22-rdf-syntax-ns#nil>
    - journal = <\_@#a674639524>
    - titre = '{P-Grid}: A Self-organizing Structured P2P System'
    - date =

      <Date@>
      :   - année = '2003'

<Article@a475526642>
:   - auteurs =

      <Personnes@>
      :   - rdf:first = <\_@#a74993404>
          - rdf:rest = <\_@http://www.w3.org/1999/02/22-rdf-syntax-ns#nil>
    - journal = <\_@#a70981683>
    - titre = 'Binary Codes capable of correcting deletions, insertions, and reversals'
    - date =

      <Date@>
      :   - année = '1996'

<ExtraitLivre@a71568377>
:   - auteurs =

      <Personnes@>
      :   - rdf:first = <\_@#a11090777>
          - rdf:rest =

            <Personnes@>
            :   - rdf:first = <\_@#a431956276>
                - rdf:rest = <\_@http://www.w3.org/1999/02/22-rdf-syntax-ns#nil>
    - livre = <\_@#a108048723>
    - titre = 'Database integration: the key to data interoperability'
    - éditeurs =

      <Personnes@>
      :   - rdf:first = <\_@#a431956276>
          - rdf:rest =

            <Personnes@>
            :   - rdf:first = <\_@#a431956276b>
                - rdf:rest =

                  <Personnes@>
                  :   - rdf:first = <\_@#a431956276c>
                      - rdf:rest = <\_@http://www.w3.org/1999/02/22-rdf-syntax-ns#nil>

<Actes@a72192307>
:   - éditeur = <\_@#a131020767>
    - titre = 'Proc. Of the 13th Int. Conference on Knowledge Engineering and Management (EKAW-2002)'
    - évènement = <\_@#a72192307c>
    - date =

      <Date@>
      :   - année = '2002'

<Actes@a32071928>
:   - éditeur = <\_@#a131020767>
    - évènement = <\_@#a32071928c>
    - titre = 'Proceedings of the First European Semantic Web Symposium'
    - date =

      <Date@>
      :   - année = '2004'

<Divers@a140583454>
:   - auteurs =

      <Personnes@>
      :   - rdf:first = <\_@#a712561038>
          - rdf:rest =

            <Personnes@>
            :   - rdf:first = <\_@#a71003986>
                - rdf:rest = <\_@http://www.w3.org/1999/02/22-rdf-syntax-ns#nil>
    - titre = '{QOM} - Quick Ontology Mapping'
    - note = 'submitted to the ISWC 04'
    - date =

      <Date@>
      :   - année = '2004'

<ExtraitActes@a11065952>
:   - auteurs =

      <Personnes@>
      :   - rdf:first = <\_@#a712561038>
          - rdf:rest =

            <Personnes@>
            :   - rdf:first = <\_@#a79573306>
                - rdf:rest = <\_@http://www.w3.org/1999/02/22-rdf-syntax-ns#nil>
    - actes = <\_@#a32071928>
    - titre = 'Ontology Mapping - An Integrated Approach'
    - url = 'http://www.aifb.uni-karlsruhe.de/WBS/meh/publications/ehrig04ontology\_ESWS04.pdf'

<Article@a80299267>
:   - auteurs =

      <Personnes@>
      :   - rdf:first = <\_@#a29105611>
          - rdf:rest =

            <Personnes@>
            :   - rdf:first = <\_@#a98078619>
                - rdf:rest =

                  <Personnes@>
                  :   - rdf:first = <\_@#a337716610>
                      - rdf:rest = <\_@http://www.w3.org/1999/02/22-rdf-syntax-ns#nil>
    - journal = <\_@#a246119474>
    - titre = 'Start making sense: The Chatty Web approach for global semantic agreements'
    - date =

      <Date@>
      :   - mois = '--12'
          - année = '2003'

<Monographie@a108048723>
:   - éditeur = <\_@#a85849488>
    - titre = 'Object-Oriented Data Modeling'
    - date =

      <Date@>
      :   - année = '2000'

<Article@a456080390>
:   - auteurs =

      <Personnes@>
      :   - rdf:first = <\_@#a093016135>
          - rdf:rest =

            <Personnes@>
            :   - rdf:first = <\_@#a572406328>
                - rdf:rest =

                  <Personnes@>
                  :   - rdf:first = <\_@#a086379337>
                      - rdf:rest =

                        <Personnes@>
                        :   - rdf:first = <\_@#a04570373>
                            - rdf:rest = <\_@http://www.w3.org/1999/02/22-rdf-syntax-ns#nil>
    - journal = <\_@#a362042121>
    - titre = 'On dynamically generated ontology translators in agent communication'
    - pages =

      <IntervalleDePages@>
      :   - pageDébut = '587'
          - pageFin = '607'
    - date =

      <Date@>
      :   - mois = '--12'
          - année = '2001'

<Article@a846015923>
:   - auteurs =

      <Personnes@>
      :   - rdf:first = <\_@#a900366022>
          - rdf:rest =

            <Personnes@>
            :   - rdf:first = <\_@#a467748807>
                - rdf:rest =

                  <Personnes@>
                  :   - rdf:first = <\_@#a971541439>
                      - rdf:rest =

                        <Personnes@>
                        :   - rdf:first = <\_@#a066600210>
                            - rdf:rest = <\_@http://www.w3.org/1999/02/22-rdf-syntax-ns#nil>
    - journal = <\_@#a906774044>
    - titre = 'A Framework for Modeling and Evaluating Automatic Semantic Reconciliation'
    - note = 'to appear'
    - date =

      <Date@>
      :   - année = '2004'

---

Generated by OWL2HTML
