# Supplementary material for: Matching sensor ontologies through siamese neural networks without using reference alignment
Source: PeerJ Comput Sci. 2021 Jun 18;7:e602. doi: 10.7717/peerj-cs.602 (PMC8237319; doi:10.7717/peerj-cs.602)
Supplement: Supplemental Information 1 [file peerj-cs-07-602-s001.zip › 210/refalign.html]

# (level 0) Alignment

## Source: http://oaei.ontologymatching.org/2011/benchmarks/101/onto.rdf

## Target: http://oaei.ontologymatching.org/2011/benchmarks/210/onto.rdf

## Correspondences

type = catégorie
:   1.0

howPublished = modeDePublication
:   1.0

periodicity = périodicité
:   1.0

proceedings = actes
:   1.0

volume = volume
:   1.0

annote = annote
:   1.0

PersonList = Personnes
:   1.0

month = mois
:   1.0

copyright = droits
:   1.0

Unpublished = NonPublié
:   1.0

address = adresse
:   1.0

Address = Adresse
:   1.0

chapter = chapitre
:   1.0

Chapter = Chapitre
:   1.0

editor = éditeurs
:   1.0

InBook = ExtraitLivre
:   1.0

Date = Date
:   1.0

series = série
:   1.0

PageRange = IntervalleDePages
:   1.0

date = date
:   1.0

title = titre
:   1.0

Booklet = Livret
:   1.0

numberOrVolume = numéroOuVolume
:   1.0

LectureNotes = Polycopié
:   1.0

url = url
:   1.0

MastersThesis = MémoireDeMastère
:   1.0

organizer = organisateur
:   1.0

mrNumber = noMR
:   1.0

pages = pages
:   1.0

TechReport = RaportTechnique
:   1.0

reviewed = Évalué
:   1.0

startPage = pageDébut
:   1.0

edition = édition
:   1.0

lccn = lccn
:   1.0

affiliation = affiliation
:   1.0

institution = institution
:   1.0

year = année
:   1.0

isPartOf = partieDe
:   1.0

organization = organisation
:   1.0

country = pays
:   1.0

publisher = éditeur
:   1.0

school = institution
:   1.0

Misc = Divers
:   1.0

collection = collection
:   1.0

Collection = Compilation
:   1.0

isbn = isbn
:   1.0

abstract = résumé
:   1.0

directors = réalisateurs
:   1.0

Academic = Mémoire
:   1.0

location = localisation
:   1.0

MotionPicture = Film
:   1.0

Article = Article
:   1.0

Informal = Informel
:   1.0

price = prix
:   1.0

name = nom
:   1.0

event = évènement
:   1.0

state = état
:   1.0

Book = Livre
:   1.0

book = livre
:   1.0

day = jour
:   1.0

School = Université
:   1.0

shortName = nomCourt
:   1.0

PhdThesis = MémoireDeDoctorat
:   1.0

Proceedings = Actes
:   1.0

number = numéro
:   1.0

issue = sortie
:   1.0

Reference = Référence
:   1.0

endPage = pageFin
:   1.0

InCollection = ExtraitCompilation
:   1.0

firstPublished = dateDePublication
:   1.0

author = auteurs
:   1.0

Report = Raport
:   1.0

note = note
:   1.0

humanCreator = créateur
:   1.0

Conference = Conférence
:   1.0

Part = Partie
:   1.0

Publisher = Éditeur
:   1.0

contract = contrat
:   1.0

Manual = Manuel
:   1.0

key = Clé
:   1.0

InProceedings = ExtraitActes
:   1.0

Journal = Revue
:   1.0

journal = journal
:   1.0

Monograph = Monographie
:   1.0

keywords = mots-clé
:   1.0

issn = issn
:   1.0

contents = contenu
:   1.0

city = ville
:   1.0

Institution = Institution
:   1.0

Deliverable = Livrable
:   1.0

size = taille
:   1.0

chapters = chapitres
:   1.0

parts = parties
:   1.0

communications = communications
:   1.0

articles = articles
:   1.0

lastName = lastName
:   1.0

language = langage
:   1.0
