# Supplementary material for: Matching sensor ontologies through siamese neural networks without using reference alignment
Source: PeerJ Comput Sci. 2021 Jun 18;7:e602. doi: 10.7717/peerj-cs.602 (PMC8237319; doi:10.7717/peerj-cs.602)
Supplement: Supplemental Information 1 [file peerj-cs-07-602-s001.zip › 222/onto.html]

Bibliographic references


# Bibliographic references

Bibliographic references in OWL

*Possible ontology to describe bibTeX entries.*  
Author: Nick Knouf <nknouf@mit.edu>  
Contributor: Antoine Zimmermann <antoine.zimmermann@inrialpes.fr>, Jérôme Euzenat,   
Date: 08/06/2005  
Version: $Id: onto-flat.rdf,v 1.15 2011/05/31 15:37:47 euzenat Exp $

## Classes

**http://www.w3.org/1999/02/22-rdf-syntax-ns#List** (, *)*


**http://xmlns.com/foaf/0.1/Person** (, *)*


**http://xmlns.com/foaf/0.1/Organization** (, *)*


**Reference** (Reference, *Base class for all entries)*
:   - #date [0 1]
    - #title [0 1]
    - #humanCreator [0 1]

    **Article** (Article, *An article from a journal or magazine.)*
    :   - #title [1 1]
        - #author [1 1]
        - #pages [0 1] [1 1]
        - #journal [1 1]
        - #date [1 1] *#Date*
        - #number [0 1]
        - #volume [0 1]

    **Monograph** (Monograph, *A book that is a single entity, as opposed to a collection.)*
    :   - #title [1 1]
        - #volume [0 1]
        - #publisher [0 1]
        - #series [0 1]
        - #date [1 1] *#Date*
        - #author [1 1]
        - #edition [0 1]
        - #chapters *#Chapter*

    **Collection** (Collection, *A book that is collection of texts or articles.)*
    :   - #title [1 1]
        - #volume [0 1]
        - #publisher [0 1]
        - #series [0 1]
        - #date [1 1] *#Date*
        - #author [1 1]
        - #edition [0 1]
        - #chapters *#Chapter*
        - #parts *#InCollection*

    **Booklet** (Booklet, *A work that is printed and bound, but without a named publisher or sponsoring institution.)*
    :   - #title [1 1]

    **Chapter** (BookPart, *A chapter (or section or whatever) of a book having its own title.)*
    :   - #pages [0 1]
        - #title [1 1]
        - #chapter [0 1] [0 1]

    **InBook** (InBook, *A subpart of a book given by a range of pages.)*
    :   - #title [1 1]
        - #author [1 1]
        - #pages [0 1] [1 +oo]
        - #book [1 1]

    **InCollection** (Incollection, *A part of a book having its own title.)*
    :   - #pages [0 1]
        - #title [1 1]
        - #author [1 1]
        - #collection [1 1]

    **InProceedings** (InProceedings, *An article in a conference proceedings.)*
    :   - #pages [0 1]
        - #title [1 1]
        - #author [1 1]
        - #proceedings [1 1]

    **LectureNotes** (LectureNotes, *Lecture notes.)*
    :   - #title [1 1]

    **Manual** (Manual, *Technical documentation.)*
    :   - #organization [0 1]
        - #edition [0 1]
        - #title [1 1] [1 1]

    **MastersThesis** (MastersThesis, *A Master's thesis.)*
    :   - #author [1 1]
        - #title [1 1]
        - #school [1 1]
        - #date [1 1]

    **PhdThesis** (PhdThesis, *A PhD thesis.)*
    :   - #author [1 1]
        - #title [1 1]
        - #school [1 1]
        - #date [1 1]

    **Misc** (Misc, *Use this type when nothing else fits.)*


    **Proceedings** (Proceedings, *The proceedings of a conference.)*
    :   - #title [1 1]
        - #volume [0 1]
        - #publisher [0 1]
        - #series [0 1]
        - #date [1 1] *#Date*
        - #author [1 1]
        - #edition [0 1]
        - #communications *#InProceedings*
        - #event [0 1] *#Conference*
        - #editor [0 1]
        - #organization [0 1]

    **Report** (Report, *A report published by an institution with some explicit policy.)*
    :   - #author [1 1]
        - #title [1 1]
        - #institution [1 1]
        - #date [1 1] *#Date*
        - #number [0 1]

        **TechReport** (TechReport, *A report published by a school or other institution, usually numbered within a series.)*


        **Deliverable** (Deliverable report, *A report delivered for accomplishing a contract.)*
        :   - #contract [0 1]

    **Unpublished** (Unpublished, *A document having an author and title, but not formally published.)*
    :   - #author [1 1]
        - #title [1 1] [1 1]
        - #note [1 +oo]

    **MotionPicture** (MotionPicture, *A film/movie/motion picture.)*

**Journal** (Journal or magazine, *A periodical publication collecting works from different authors.)*
:   - #name [1 1] *http://www.w3.org/2001/XMLSchema#string*
    - #shortName *http://www.w3.org/2001/XMLSchema#string*
    - #periodicity *http://www.w3.org/2001/XMLSchema#string*
    - #publisher [0 1]
    - #series [0 1]
    - #firstPublished [0 1]
    - #articles *#Article*

**Conference** (The location of an event, *An event presenting work.)*
:   - #name [1 1]
    - #organizer *#Institution*
    - #shortName [0 1]
    - #issue [0 1]
    - #location [0 1]

**Address** (Address, *The street address of the location of some organization or event.)*
:   - #country [0 1] *http://www.w3.org/2001/XMLSchema#string*
    - #state [0 1] *http://www.w3.org/2001/XMLSchema#string*
    - #city [0 1] *http://www.w3.org/2001/XMLSchema#string*

**Institution** (Institution, *An institution.)*
:   super: *http://xmlns.com/foaf/0.1/Organization*  

    - #name [1 1]
    - #shortName [1 1]
    - #address [0 1]

    **Publisher** (Publisher, *The publisher of books or journals.)*


    **School** (School, *A school or university.)*

**PersonList** (Person list, *A list of persons.)*
:   super: *http://www.w3.org/1999/02/22-rdf-syntax-ns#List*  

    - http://www.w3.org/1999/02/22-rdf-syntax-ns#first [1 1] *http://xmlns.com/foaf/0.1/Person*
    - http://www.w3.org/1999/02/22-rdf-syntax-ns#rest [1 1] (*#PersonList* |  {

      <rdf:List@ttp://www.w3.org/1999/02/22-rdf-syntax-ns#nil>
      } )

**PageRange** (PageRange, *A range of pages.)*
:   - #startPage [1 1]
    - #endPage [1 1]

**Date** (Date, *Date of a day which can be unknown (i.e., only the year is known or only the year and month). This is for overcoming the limits of XML-Schema for wich a date is not separable.)*
:   - #year [1 1] *http://www.w3.org/2001/XMLSchema#gYear*
    - #month [0 1] *http://www.w3.org/2001/XMLSchema#gMonth*
    - #day [0 1] *http://www.w3.org/2001/XMLSchema#gDay*

## Properties

**http://www.w3.org/1999/02/22-rdf-syntax-ns#first**: http://www.w3.org/1999/02/22-rdf-syntax-ns#List -> \_ *()*


**http://www.w3.org/1999/02/22-rdf-syntax-ns#rest**: http://www.w3.org/1999/02/22-rdf-syntax-ns#List -> http://www.w3.org/1999/02/22-rdf-syntax-ns#List *()*


**chapters**: #Reference -> #Chapter *(The chapters of a book (monograph or collection).)*


**parts**: #Reference -> #Reference *(The parts of some reference (chapters, sections).)*


**communications**: #Proceedings -> #InProceedings *(The communications to a conference as collected in proceedings.)*


**articles**: #Journal -> #Article *(The papers published by a journal.)*


**address**: http://www.w3.org/2002/07/owl#Thing -> #Address *(The address of an institution or the place of a conference.)*


**event**: #Proceedings -> #Conference *(The event concerned with the proceedings.)*


**organizer**: #Conference -> http://xmlns.com/foaf/0.1/Organization *(The organization taking in charge a conference.)*


**contract**: #Reference -> http://www.w3.org/2002/07/owl#Thing *(The contract in relation of which a particular reference has been made.)*


**humanCreator**: #Reference -> #PersonList *(The list of the human creators of a work.)*
:   **author**: \_ -> \_ *(The list of the author(s) of a work.)*


    **editor**: \_ -> \_ *(The list of persons who edited or coordinated a work.)*


    **directors**: #MotionPicture -> \_ *(The list of directors of a motion picture or theater play.)*

**institution**: #Report -> #Institution *(The sponsoring institution of a technical report.)*


**isPartOf**: #Reference -> \_ *(The document that contains a text or article.)*
:   **journal**: #Article -> #Journal *(The journal in which an article is published.)*


    **book**: #InBook -> #Monograph *(A reference to the book that contains the entry.)*


    **collection**: #InCollection -> #Collection *(A reference to the book that contains the entry.)*


    **proceedings**: #InProceedings -> #Proceedings *(A reference to the proceedings in which the entry appears.)*

**date**: (*#Reference* | *#Conference*) -> #Date *(The date when the work was published or, for an unpublished work, in which it was written. This date can be incomplete (i.e., no known day or month) but must contain a year.)*


**organization**: (*#Proceedings* | *#Manual*) -> http://xmlns.com/foaf/0.1/Organization *(The organization that sponsors a conference or that publishes a manual.)*


**publisher**: (*#Reference* | *#Journal*) -> #Publisher *(The publisher of a work.)*


**school**: #Reference -> #School *(The name of the school where a thesis was written.)*


**location**: (*#Reference* | *#Conference*) -> #Address *(A location associated with the entry, such as the city in which a conference took place (this is now obsolete).)*


**pages**: #Reference -> #PageRange *(A range of page, with an upper and lower limit.)*

**http://purl.org/dc/elements/1.1/creator**\_ -> \_ *()*


**http://purl.org/dc/elements/1.1/contributor**\_ -> \_ *()*


**http://purl.org/dc/elements/1.1/description**\_ -> \_ *()*


**http://purl.org/dc/elements/1.1/date**\_ -> \_ *()*


**http://xmlns.com/foaf/0.1/firstName**\_ -> \_ *()*


**#lastName**\_ -> \_ *()*


**http://xmlns.com/foaf/0.1/name**\_ -> \_ *()*


**key** #Reference -> http://www.w3.org/2001/XMLSchema#string *(The key for a particular entry. Note that the rdf:ID for each Reference instance could be the key as well, possibly making this property redundant.)*


**reviewed** #Reference -> http://www.w3.org/2001/XMLSchema#string *(The selection process overcome by the publication (full, none, poster, invited are possible values).)*


**annote** #Reference -> http://www.w3.org/2001/XMLSchema#string *(An annotation. It is not used by the standard bibliography styles, but may be used by others that produce an annotated bibliography.)*


**periodicity** #Journal -> http://www.w3.org/2001/XMLSchema#string *(The periodicity of a serial publication (yearly, biannual, quarterly, monthly, bimonthly, weekly, biweekly, dayly).)*


**firstPublished** #Chapter -> http://www.w3.org/2001/XMLSchema#string *(When the reference was first published.)*


**edition** #Reference -> http://www.w3.org/2001/XMLSchema#string *(The edition of a book (for example, "Second"). This should be an ordinal.)*


**howPublished**(*#Misc* | *#Booklet*) -> http://www.w3.org/2001/XMLSchema#string *(How something strange has been published.)*


**note** #Reference -> http://www.w3.org/2001/XMLSchema#string *(Any additional information that can help the reader.)*


**series** #Reference -> http://www.w3.org/2001/XMLSchema#string *(The name of a series or set of books. When citing an entire book, the the title field gives its title and an optional series field gives the name of a series or multi-volume set in which the book is published.)*


**title** #Reference -> http://www.w3.org/2001/XMLSchema#string *(The title of the work.)*


**type** #Reference -> http://www.w3.org/2001/XMLSchema#string *(The type of a technical report (for example, "Research Note").)*


**affiliation** #Reference -> http://www.w3.org/2001/XMLSchema#string *(The authors affiliation.)*


**abstract** #Reference -> http://www.w3.org/2001/XMLSchema#string *(The abstract of the work.)*


**contents** #Reference -> http://www.w3.org/2001/XMLSchema#string *(The Table of Contents.)*


**copyright** #Reference -> http://www.w3.org/2001/XMLSchema#string *(The Copyright information.)*


**isbn** #Reference -> http://www.w3.org/2001/XMLSchema#string *(The International Standard Book Number.)*


**issn** #Reference -> http://www.w3.org/2001/XMLSchema#string *(The International Standard Serial Number. Used to identify a journal.)*


**keywords** #Reference -> http://www.w3.org/2001/XMLSchema#string *(Key words used for searching or possibly for annotation (as given by the author or publisher).)*


**language** #Reference -> http://www.w3.org/2001/XMLSchema#language *(The language in which the referenced publication is written or performed (use ISO two-letter codes).)*


**lccn** #Reference -> http://www.w3.org/2001/XMLSchema#string *(The Library of Congress Call Number (this the Congress of the United State of America).)*


**mrNumber** #Reference -> http://www.w3.org/2001/XMLSchema#string *(The Mathematical Reviews number.)*


**price** #Reference -> http://www.w3.org/2001/XMLSchema#string *(The price of the document.)*


**size** #Reference -> http://www.w3.org/2001/XMLSchema#string *(The physical dimensions of a document.)*


**url** #Reference -> http://www.w3.org/2001/XMLSchema#string *(The Universal Resource Locator that points to the item being referenced.)*


**name**\_ -> http://www.w3.org/2001/XMLSchema#string *()*


**shortName**\_ -> http://www.w3.org/2001/XMLSchema#string *()*


**chapter** #Reference -> http://www.w3.org/2001/XMLSchema#string *(The chapter (or section or whatever) number in which the entry is found.)*


**numberOrVolume**(*#Reference* | *#Conference*) -> \_ *(The number of a journal, magazine, technical report, or of a work in a series. An issue of a journal or magazine is usually identified by its volume and number; the organization that issues a technical report usually gives it a number; and sometimes books are given numbers in a named series.)*
:   **number** #Reference -> http://www.w3.org/2001/XMLSchema#string *(The number of a journal, magazine, technical report, or of a work in a series. An issue of a journal or magazine is usually identified by its volume and number; the organization that issues a technical report usually gives it a number; and sometimes books are given numbers in a named series.)*


    **issue**(*#Reference* | *#Conference*) -> http://www.w3.org/2001/XMLSchema#string *(The issue of a conference.)*


    **volume** #Reference -> http://www.w3.org/2001/XMLSchema#nonNegativeInteger *(The volume of a journal or multivolume book.)*

**year** #Date -> http://www.w3.org/2001/XMLSchema#gYear *()*


**month** #Date -> http://www.w3.org/2001/XMLSchema#gMonth *()*


**day** #Date -> http://www.w3.org/2001/XMLSchema#gDay *()*


**city** #Address -> http://www.w3.org/2001/XMLSchema#string *()*


**state** #Address -> http://www.w3.org/2001/XMLSchema#string *()*


**country** #Address -> http://www.w3.org/2001/XMLSchema#string *(Usually in ISO format)*


**startPage** #PageRange -> http://www.w3.org/2001/XMLSchema#nonNegativeInteger *(The beginning of a range of pages.)*


**endPage** #PageRange -> http://www.w3.org/2001/XMLSchema#nonNegativeInteger *(The end of a range of pages.)*

## Individuals

<rdf:List@ttp://www.w3.org/1999/02/22-rdf-syntax-ns#nil>


<foaf:Person@a04570373>
:   - rdfs:label = 'John-Jules Meyer'
    - foaf:name = 'John-Jules Meyer'
    - foaf:firstName = 'John-Jules'
    - lastName = 'Meyer'

<foaf:Person@a43836633>
:   - rdfs:label = 'Jeen Broekstra'
    - foaf:name = 'Jeen Broekstra'
    - foaf:firstName = 'Jeen'
    - lastName = 'Broekstra'

<foaf:Person@a85228505>
:   - rdfs:label = 'Alexander Mädche'
    - foaf:name = 'Alexander Mädche'
    - foaf:firstName = 'Alexander'
    - lastName = 'Mädche'

<foaf:Person@a48552212>
:   - rdfs:label = 'Björn Schnizler'
    - foaf:name = 'Björn Schnizler'
    - foaf:firstName = 'Björn'
    - lastName = 'Schnizler'

<foaf:Person@a971541439>
:   - rdfs:label = 'Alberto Trombetta'
    - foaf:name = 'Alberto Trombetta'
    - foaf:firstName = 'Alberto'
    - lastName = 'Trombetta'

<foaf:Person@a11090777>
:   - rdfs:label = 'Christine Parent'
    - foaf:name = 'Christine Parent'
    - foaf:firstName = 'Christine'
    - lastName = 'Parent'

<foaf:Person@a250331360>
:   - rdfs:label = 'R. Schmidt'
    - foaf:name = 'R. Schmidt'
    - foaf:firstName = 'R.'
    - lastName = 'Schmidt'

<foaf:Person@a79573306>
:   - rdfs:label = 'York Sure'
    - foaf:name = 'York Sure'
    - foaf:firstName = 'York'
    - lastName = 'Sure'

<foaf:Person@a885257047>
:   - rdfs:label = 'M. Punceva'
    - foaf:name = 'M. Punceva'
    - foaf:firstName = 'M.'
    - lastName = 'Punceva'

<foaf:Person@a74993404>
:   - rdfs:label = 'I. V. Levenshtein'
    - foaf:name = 'I. V. Levenshtein'
    - foaf:firstName = 'I. V.'
    - lastName = 'Levenshtein'

<foaf:Person@a71003986>
:   - rdfs:label = 'Steffen Staab'
    - foaf:name = 'Steffen Staab'
    - foaf:firstName = 'Steffen'
    - lastName = 'Staab'

<foaf:Person@a572406328>
:   - rdfs:label = 'Frank Boer'
    - foaf:name = 'Frank Boer'
    - foaf:firstName = 'Frank'
    - lastName = 'Boer'

<foaf:Person@a139477786>
:   - rdfs:label = 'Maarten Menken'
    - foaf:name = 'Maarten Menken'
    - foaf:firstName = 'Maarten'
    - lastName = 'Menken'

<foaf:Person@a337716610>
:   - rdfs:label = 'Manfred Hauswirth'
    - foaf:name = 'Manfred Hauswirth'
    - foaf:firstName = 'Manfred'
    - lastName = 'Hauswirth'

<foaf:Person@a086379337>
:   - rdfs:label = 'Wiebe Hoek'
    - foaf:name = 'Wiebe Hoek'
    - foaf:firstName = 'Wiebe'
    - lastName = 'Hoek'

<foaf:Person@a712561038>
:   - rdfs:label = 'Marc Ehrig'
    - foaf:name = 'Marc Ehrig'
    - foaf:firstName = 'Marc'
    - lastName = 'Ehrig'

<foaf:Person@a066600210>
:   - rdfs:label = 'Danilo Montesi'
    - foaf:name = 'Danilo Montesi'
    - foaf:firstName = 'Danilo'
    - lastName = 'Montesi'

<foaf:Person@a093016135>
:   - rdfs:label = 'Rogier Eijk'
    - foaf:name = 'Rogier Eijk'
    - foaf:firstName = 'Rogier'
    - lastName = 'Eijk'

<foaf:Person@a944339054>
:   - rdfs:label = 'Frank van Harmelen'
    - foaf:name = 'Frank van Harmelen'
    - foaf:firstName = 'Frank'
    - lastName = 'van Harmelen'

<foaf:Person@a98078619>
:   - rdfs:label = 'Philippe Cudré-Mauroux'
    - foaf:name = 'Philippe Cudré-Mauroux'
    - foaf:firstName = 'Philippe'
    - lastName = 'Cudré-Mauroux'

<foaf:Person@a39510672>
:   - rdfs:label = 'Z. Despotovic'
    - foaf:name = 'Z. Despotovic'
    - foaf:firstName = 'Z.'
    - lastName = 'Despotovic'

<foaf:Person@a431956276>
:   - rdfs:label = 'Stefano Spaccapietra'
    - foaf:name = 'Stefano Spaccapietra'
    - foaf:firstName = 'Stefano'
    - lastName = 'Spaccapietra'

<foaf:Person@a431956276b>
:   - rdfs:label = 'Mike Papazoglou'
    - foaf:name = 'Mike Papazoglou'
    - foaf:firstName = 'Mike'
    - lastName = 'Papazoglou'

<foaf:Person@a431956276c>
:   - rdfs:label = 'Zahir Tari'
    - foaf:name = 'Zahir Tari'
    - foaf:firstName = 'Zahir'
    - lastName = 'Tari'

<foaf:Person@a70955601>
:   - rdfs:label = 'A. Datta'
    - foaf:name = 'A. Datta'
    - foaf:firstName = 'A.'
    - lastName = 'Datta'

<foaf:Person@a467748807>
:   - rdfs:label = 'Ateret Anaby-Tavor'
    - foaf:name = 'Ateret Anaby-Tavor'
    - foaf:firstName = 'Ateret'
    - lastName = 'Anaby-Tavor'

<foaf:Person@a3105947>
:   - rdfs:label = 'Ronny Siebes'
    - foaf:name = 'Ronny Siebes'
    - foaf:firstName = 'Ronny'
    - lastName = 'Siebes'

<foaf:Person@a29105611>
:   - rdfs:label = 'Karl Aberer'
    - foaf:name = 'Karl Aberer'
    - foaf:firstName = 'Karl'
    - lastName = 'Aberer'

<foaf:Person@a958684218>
:   - rdfs:label = 'Peter Mika'
    - foaf:name = 'Peter Mika'
    - foaf:firstName = 'Peter'
    - lastName = 'Mika'

<foaf:Person@a94533498>
:   - rdfs:label = 'Peter Haase'
    - foaf:name = 'Peter Haase'
    - foaf:firstName = 'Peter'
    - lastName = 'Haase'

<foaf:Person@a900366022>
:   - rdfs:label = 'Avigdor Gal'
    - foaf:name = 'Avigdor Gal'
    - foaf:firstName = 'Avigdor'
    - lastName = 'Gal'

<Journal@a246119474>
:   - rdfs:label = 'Journal of Web Semantics'
    - name = 'Journal of Web Semantics'
    - shortName = 'JWS'

<Publisher@a131020767>
:   - rdfs:label = 'Springer-Verlag'
    - name = 'Springer-Verlag'
    - address =

      <Address@>
      :   - city = 'Heidelberg'
          - country = 'DE'

<Journal@a70981683>
:   - rdfs:label = 'Cybernetics and Control Theory'
    - name = 'Cybernetics and Control Theory'

<Publisher@a85849488>
:   - rdfs:label = 'The MIT Press'
    - name = 'The MIT Press'
    - address =

      <Address@>
      :   - city = 'Cambridge'
          - state = 'MA'
          - country = 'US'

<Journal@a362042121>
:   - rdfs:label = 'International journal of intelligent system'
    - name = 'International journal of intelligent system'
    - shortName = 'IJIS'

<Journal@a674639524>
:   - rdfs:label = 'ACM SIGMOD Record'
    - name = 'ACM SIGMOD Record'

<Journal@a906774044>
:   - rdfs:label = 'VLDB Journal'
    - name = 'VLDB Journal'

<Conference@spg04>
:   - rdfs:label = 'SemPGrid 04 Workshop'
    - name = 'SemPGrid 04 Workshop'
    - location =

      <Address@>
      :   - city = 'New-York'
          - state = 'NY'
          - country = 'US'
    - date =

      <Date@>
      :   - month = '--05'
          - year = '2004'

<Conference@a72192307c>
:   - rdfs:label = '13th Int. Conference on Knowledge Engineering and Management (EKAW-2002)'
    - name = 'Int. Conference on Knowledge Engineering and Management'
    - shortName = 'EKAW'
    - issue = '13'
    - date =

      <Date@>
      :   - month = '--10'
          - year = '2002'

<Conference@a32071928c>
:   - rdfs:label = 'First European Semantic Web Symposium'
    - name = 'European Semantic Web Symposium'
    - shortName = 'ESWS'
    - issue = '1'
    - location =

      <Address@>
      :   - city = 'Heraklion'
          - country = 'GR'
    - date =

      <Date@>
      :   - month = '--05'
          - year = '2004'

<Proceedings@a060097576>
:   - rdfs:label = 'Proceedings of the SemPGrid 04 Workshop'
    - title = 'Proceedings of the SemPGrid 04 Workshop'
    - date =

      <Date@>
      :   - year = '2004'
    - event = <\_@#spg04>

<InProceedings@a64263824>
:   - rdfs:label = 'Bibster - A Semantics-Based Bibliographic Peer-to-Peer System'
    - author =

      <PersonList@>
      :   - rdf:first = <\_@#a43836633>
          - rdf:rest =

            <PersonList@>
            :   - rdf:first = <\_@#a712561038>
                - rdf:rest =

                  <PersonList@>
                  :   - rdf:first = <\_@#a94533498>
                      - rdf:rest =

                        <PersonList@>
                        :   - rdf:first = <\_@#a944339054>
                            - rdf:rest =

                              <PersonList@>
                              :   - rdf:first = <\_@#a139477786>
                                  - rdf:rest =

                                    <PersonList@>
                                    :   - rdf:first = <\_@#a958684218>
                                        - rdf:rest =

                                          <PersonList@>
                                          :   - rdf:first = <\_@#a48552212>
                                              - rdf:rest =

                                                <PersonList@>
                                                :   - rdf:first = <\_@#a3105947>
                                                    - rdf:rest = <\_@http://www.w3.org/1999/02/22-rdf-syntax-ns#nil>
    - proceedings = <\_@#a060097576>
    - title = 'Bibster - A Semantics-Based Bibliographic Peer-to-Peer System'

<InProceedings@a439508789>
:   - rdfs:label = 'Measuring Similarity between Ontologies'
    - author =

      <PersonList@>
      :   - rdf:first = <\_@#a85228505>
          - rdf:rest =

            <PersonList@>
            :   - rdf:first = <\_@#a71003986>
                - rdf:rest = <\_@http://www.w3.org/1999/02/22-rdf-syntax-ns#nil>
    - proceedings = <\_@#a72192307>
    - title = 'Measuring Similarity between Ontologies'

<Article@a492378321>
:   - rdfs:label = '{P-Grid: A Self-organizing Structured P2P System}'
    - author =

      <PersonList@>
      :   - rdf:first = <\_@#a29105611>
          - rdf:rest =

            <PersonList@>
            :   - rdf:first = <\_@#a98078619>
                - rdf:rest =

                  <PersonList@>
                  :   - rdf:first = <\_@#a70955601>
                      - rdf:rest =

                        <PersonList@>
                        :   - rdf:first = <\_@#a39510672>
                            - rdf:rest =

                              <PersonList@>
                              :   - rdf:first = <\_@#a337716610>
                                  - rdf:rest =

                                    <PersonList@>
                                    :   - rdf:first = <\_@#a885257047>
                                        - rdf:rest =

                                          <PersonList@>
                                          :   - rdf:first = <\_@#a250331360>
                                              - rdf:rest = <\_@http://www.w3.org/1999/02/22-rdf-syntax-ns#nil>
    - journal = <\_@#a674639524>
    - title = '{P-Grid}: A Self-organizing Structured P2P System'
    - date =

      <Date@>
      :   - year = '2003'

<Article@a475526642>
:   - rdfs:label = 'Binary Codes capable of correcting deletions, insertions, and reversals'
    - author =

      <PersonList@>
      :   - rdf:first = <\_@#a74993404>
          - rdf:rest = <\_@http://www.w3.org/1999/02/22-rdf-syntax-ns#nil>
    - journal = <\_@#a70981683>
    - title = 'Binary Codes capable of correcting deletions, insertions, and reversals'
    - date =

      <Date@>
      :   - year = '1996'

<InBook@a71568377>
:   - rdfs:label = 'Database integration: the key to data interoperability'
    - author =

      <PersonList@>
      :   - rdf:first = <\_@#a11090777>
          - rdf:rest =

            <PersonList@>
            :   - rdf:first = <\_@#a431956276>
                - rdf:rest = <\_@http://www.w3.org/1999/02/22-rdf-syntax-ns#nil>
    - book = <\_@#a108048723>
    - title = 'Database integration: the key to data interoperability'
    - editor =

      <PersonList@>
      :   - rdf:first = <\_@#a431956276>
          - rdf:rest =

            <PersonList@>
            :   - rdf:first = <\_@#a431956276b>
                - rdf:rest =

                  <PersonList@>
                  :   - rdf:first = <\_@#a431956276c>
                      - rdf:rest = <\_@http://www.w3.org/1999/02/22-rdf-syntax-ns#nil>

<Proceedings@a72192307>
:   - rdfs:label = 'Proc. Of the 13th Int. Conference on Knowledge Engineering and Management (EKAW-2002)'
    - publisher = <\_@#a131020767>
    - title = 'Proc. Of the 13th Int. Conference on Knowledge Engineering and Management (EKAW-2002)'
    - event = <\_@#a72192307c>
    - date =

      <Date@>
      :   - year = '2002'

<Proceedings@a32071928>
:   - rdfs:label = 'Proceedings of the First European Semantic Web Symposium'
    - publisher = <\_@#a131020767>
    - event = <\_@#a32071928c>
    - title = 'Proceedings of the First European Semantic Web Symposium'
    - date =

      <Date@>
      :   - year = '2004'

<Misc@a140583454>
:   - rdfs:label = '{QOM} - Quick Ontology Mapping'
    - author =

      <PersonList@>
      :   - rdf:first = <\_@#a712561038>
          - rdf:rest =

            <PersonList@>
            :   - rdf:first = <\_@#a71003986>
                - rdf:rest = <\_@http://www.w3.org/1999/02/22-rdf-syntax-ns#nil>
    - title = '{QOM} - Quick Ontology Mapping'
    - note = 'submitted to the ISWC 04'
    - date =

      <Date@>
      :   - year = '2004'

<InProceedings@a11065952>
:   - rdfs:label = 'Ontology Mapping - An Integrated Approach'
    - author =

      <PersonList@>
      :   - rdf:first = <\_@#a712561038>
          - rdf:rest =

            <PersonList@>
            :   - rdf:first = <\_@#a79573306>
                - rdf:rest = <\_@http://www.w3.org/1999/02/22-rdf-syntax-ns#nil>
    - proceedings = <\_@#a32071928>
    - title = 'Ontology Mapping - An Integrated Approach'
    - url = 'http://www.aifb.uni-karlsruhe.de/WBS/meh/publications/ehrig04ontology\_ESWS04.pdf'

<Article@a80299267>
:   - rdfs:label = 'Start making sense: The Chatty Web approach for global semantic agreements'
    - author =

      <PersonList@>
      :   - rdf:first = <\_@#a29105611>
          - rdf:rest =

            <PersonList@>
            :   - rdf:first = <\_@#a98078619>
                - rdf:rest =

                  <PersonList@>
                  :   - rdf:first = <\_@#a337716610>
                      - rdf:rest = <\_@http://www.w3.org/1999/02/22-rdf-syntax-ns#nil>
    - journal = <\_@#a246119474>
    - title = 'Start making sense: The Chatty Web approach for global semantic agreements'
    - date =

      <Date@>
      :   - month = '--12'
          - year = '2003'

<Monograph@a108048723>
:   - rdfs:label = 'Object-Oriented Data Modeling'
    - publisher = <\_@#a85849488>
    - title = 'Object-Oriented Data Modeling'
    - date =

      <Date@>
      :   - year = '2000'

<Article@a456080390>
:   - rdfs:label = 'On dynamically generated ontology translators in agent communication'
    - author =

      <PersonList@>
      :   - rdf:first = <\_@#a093016135>
          - rdf:rest =

            <PersonList@>
            :   - rdf:first = <\_@#a572406328>
                - rdf:rest =

                  <PersonList@>
                  :   - rdf:first = <\_@#a086379337>
                      - rdf:rest =

                        <PersonList@>
                        :   - rdf:first = <\_@#a04570373>
                            - rdf:rest = <\_@http://www.w3.org/1999/02/22-rdf-syntax-ns#nil>
    - journal = <\_@#a362042121>
    - title = 'On dynamically generated ontology translators in agent communication'
    - pages =

      <PageRange@>
      :   - startPage = '587'
          - endPage = '607'
    - date =

      <Date@>
      :   - month = '--12'
          - year = '2001'

<Article@a846015923>
:   - rdfs:label = 'A Framework for Modeling and Evaluating Automatic Semantic Reconciliation'
    - author =

      <PersonList@>
      :   - rdf:first = <\_@#a900366022>
          - rdf:rest =

            <PersonList@>
            :   - rdf:first = <\_@#a467748807>
                - rdf:rest =

                  <PersonList@>
                  :   - rdf:first = <\_@#a971541439>
                      - rdf:rest =

                        <PersonList@>
                        :   - rdf:first = <\_@#a066600210>
                            - rdf:rest = <\_@http://www.w3.org/1999/02/22-rdf-syntax-ns#nil>
    - journal = <\_@#a906774044>
    - title = 'A Framework for Modeling and Evaluating Automatic Semantic Reconciliation'
    - note = 'to appear'
    - date =

      <Date@>
      :   - year = '2004'

---

Generated by OWL2HTML
