# Supplementary material for: Matching sensor ontologies through siamese neural networks without using reference alignment
Source: PeerJ Comput Sci. 2021 Jun 18;7:e602. doi: 10.7717/peerj-cs.602 (PMC8237319; doi:10.7717/peerj-cs.602)
Supplement: Supplemental Information 1 [file peerj-cs-07-602-s001.zip › 230/onto.html]

Bibliographic references


# Bibliographic references

Bibliographic references in OWL

*Possible ontology to describe bibTeX entries.*  
Author: Nick Knouf <nknouf@mit.edu>  
Contributor: Antoine Zimmermann <antoine.zimmermann@inrialpes.fr>, Jérôme Euzenat,   
Date: 01/06/2004  
Version: $Id: onto-cflat.rdf,v 1.15 2011/05/31 15:37:28 euzenat Exp $

## Classes

**http://www.w3.org/1999/02/22-rdf-syntax-ns#List** (, *)*


**http://xmlns.com/foaf/0.1/Person** (, *)*


**http://xmlns.com/foaf/0.1/Organization** (, *)*


**Reference** (Reference, *Base class for all entries)*
:   - #title [0 1]
    - #author [0 1]
    - #year [1 1] *http://www.w3.org/2001/XMLSchema#gYear*
    - #month [0 1] *http://www.w3.org/2001/XMLSchema#gMonth*
    - #day [0 1] *http://www.w3.org/2001/XMLSchema#gDay*

    **Book** (Book, *A book that may be a monograph or a collection of written texts.)*
    :   - #title [1 1]
        - #volume [0 1]
        - #shortName [1 1]
        - #country [0 1] *http://www.w3.org/2001/XMLSchema#string*
        - #state [0 1] *http://www.w3.org/2001/XMLSchema#string*
        - #city [0 1] *http://www.w3.org/2001/XMLSchema#string*
        - #publisherName [0 1]
        - #series [0 1]
        - #year [1 1] *http://www.w3.org/2001/XMLSchema#gYear*
        - #month [0 1] *http://www.w3.org/2001/XMLSchema#gMonth*
        - #day [0 1] *http://www.w3.org/2001/XMLSchema#gDay*
        - #humanCreator [1 1]
        - #edition [0 1]

        **Monograph** (Monograph, *A book that is a single entity, as opposed to a collection.)*


        **Collection** (Collection, *A book that is collection of texts or articles.)*


        **Proceedings** (Proceedings, *The proceedings of a conference.)*
        :   - #eventName [1 1]
            - #shortName [0 1]
            - #issue [0 1]
            - #country [0 1] *http://www.w3.org/2001/XMLSchema#string*
            - #state [0 1] *http://www.w3.org/2001/XMLSchema#string*
            - #city [0 1] *http://www.w3.org/2001/XMLSchema#string*
            - #editor [0 1]
            - #organizationName *http://www.w3.org/2001/XMLSchema#string*  
               [0 1]

    **Informal** (Informal, *A document that that was informally published or not published.)*
    :   - #title [1 1]

        **Booklet** (Booklet, *A work that is printed and bound, but without a named publisher or sponsoring institution.)*


        **LectureNotes** (LectureNotes, *Lecture notes.)*


        **Manual** (Manual, *Technical documentation.)*
        :   - #organizationName [0 1]
            - #edition [0 1]
            - #title [1 1]

        **Unpublished** (Unpublished, *A document having an author and title, but not formally published.)*
        :   - #author [1 1]
            - #title [1 1]
            - #note [1 +oo]

    **Part** (Part, *A part of something (either Book or Proceedings).)*
    :   - #startPage [1 1]
        - #endPage [1 1]
        - #title [1 1]

        **Article** (Article, *An article from a journal or magazine.)*
        :   - #publisherName [0 1]
            - #series [0 1]
            - #author [1 1]
            - #startPage [1 1]
            - #endPage [1 1]
            - #journalName [1 1] *http://www.w3.org/2001/XMLSchema#string*  
               [1 1]
            - #year [1 1] *http://www.w3.org/2001/XMLSchema#gYear*
            - #month [0 1] *http://www.w3.org/2001/XMLSchema#gMonth*
            - #day [0 1] *http://www.w3.org/2001/XMLSchema#gDay*
            - #number [0 1]
            - #volume [0 1]

        **Chapter** (BookPart, *A chapter (or section or whatever) of a book having its own title.)*
        :   - #chapter [0 1] [0 1]

        **InBook** (InBook, *A subpart of a book given by a range of pages.)*
        :   - #author [1 1]
            - #startPage [1 1]
            - #endPage [1 1]
            - #book [1 1]

        **InCollection** (Incollection, *A part of a book having its own title.)*
        :   - #author [1 1]
            - #collection [1 1]

        **InProceedings** (InProceedings, *An article in a conference proceedings.)*
        :   - #author [1 1]
            - #proceedings [1 1]

    **Academic** (Academic, *A Master's or PhD thesis.)*
    :   - #author [1 1]
        - #title [1 1]
        - #schoolName [1 1]
        - #year [1 1] *http://www.w3.org/2001/XMLSchema#gYear*
        - #month [0 1] *http://www.w3.org/2001/XMLSchema#gMonth*
        - #day [0 1] *http://www.w3.org/2001/XMLSchema#gDay*

        **MastersThesis** (MastersThesis, *A Master's thesis.)*


        **PhdThesis** (PhdThesis, *A PhD thesis.)*

    **Misc** (Misc, *Use this type when nothing else fits.)*


    **Report** (Report, *A report published by an institution with some explicit policy.)*
    :   - #author [1 1]
        - #title [1 1]
        - #organizationName [1 1]
        - #year [1 1] *http://www.w3.org/2001/XMLSchema#gYear*
        - #month [0 1] *http://www.w3.org/2001/XMLSchema#gMonth*
        - #day [0 1] *http://www.w3.org/2001/XMLSchema#gDay*
        - #number [0 1]

        **TechReport** (TechReport, *A report published by a school or other institution, usually numbered within a series.)*


        **Deliverable** (Deliverable report, *A report delivered for accomplishing a contract.)*
        :   - #contract [0 1]

    **MotionPicture** (MotionPicture, *A film/movie/motion picture.)*

**PersonList** (Person list, *A list of persons.)*
:   super: *http://www.w3.org/1999/02/22-rdf-syntax-ns#List*  

    - http://www.w3.org/1999/02/22-rdf-syntax-ns#first [1 1] *http://xmlns.com/foaf/0.1/Person*
    - http://www.w3.org/1999/02/22-rdf-syntax-ns#rest [1 1] (*#PersonList* |  {

      <rdf:List@ttp://www.w3.org/1999/02/22-rdf-syntax-ns#nil>
      } )

## Properties

**http://www.w3.org/1999/02/22-rdf-syntax-ns#first**: http://www.w3.org/1999/02/22-rdf-syntax-ns#List -> \_ *()*


**http://www.w3.org/1999/02/22-rdf-syntax-ns#rest**: http://www.w3.org/1999/02/22-rdf-syntax-ns#List -> http://www.w3.org/1999/02/22-rdf-syntax-ns#List *()*


**contract**: #Reference -> http://www.w3.org/2002/07/owl#Thing *(The contract in which a particular reference has been made.)*


**humanCreator**: #Reference -> #PersonList *(A generic human creator category, necessary in order to contain both author and editor.)*
:   **author**: \_ -> \_ *(The name(s) of the author(s), in the format described in the LaTeX book.)*


    **editor**: \_ -> \_ *(The list of persons who edited or coordinated a reference.)*


    **directors**: #MotionPicture -> \_ *(The list of persons who directed the motion picture.)*

**isPartOf**: #Part -> \_ *(The document that contains a text or article.)*
:   **book**: #InBook -> #Monograph *(A reference to the book that contains the entry. The entry must be an InBook end the book a Monograph.)*


    **collection**: #InCollection -> #Collection *(A reference to the book that contains the entry. The entry must be an Inbook or Incollection.)*


    **proceedings**: #InProceedings -> #Proceedings *(A reference to the proceedings in which the entry appears. The entry must be an InProceedings.)*

**http://purl.org/dc/elements/1.1/creator**\_ -> \_ *()*


**http://purl.org/dc/elements/1.1/contributor**\_ -> \_ *()*


**http://purl.org/dc/elements/1.1/description**\_ -> \_ *()*


**http://purl.org/dc/elements/1.1/date**\_ -> \_ *()*


**http://xmlns.com/foaf/0.1/firstName**\_ -> \_ *()*


**lastName**\_ -> \_ *()*


**http://xmlns.com/foaf/0.1/name**\_ -> \_ *()*


**key** #Reference -> http://www.w3.org/2001/XMLSchema#string *(The key for a particular bibTeX entry. Note that the rdf:ID for each Reference instance could be the bibTeX key as well, possibly making this property redundant.)*


**reviewed** #Reference -> http://www.w3.org/2001/XMLSchema#string *(The selection process overcome by the publication (full, none, poster, invited are possible values).)*


**eventName** #Proceedings -> http://www.w3.org/2001/XMLSchema#string *(The event concerned with the proceedings.)*


**annote** #Reference -> http://www.w3.org/2001/XMLSchema#string *(An annotation. It is not used by the standard bibliography styles, but may be used by others that produce an annotated bibliography.)*


**edition**(*#Book* | *#Manual*) -> http://www.w3.org/2001/XMLSchema#string *(The edition of a book (for example, "Second"). This should be an ordinal, and should have the first letter capitalized, as shown here; the standard styles convert to lower case when necessary.)*


**howPublished**(*#Misc* | *#Booklet*) -> http://www.w3.org/2001/XMLSchema#string *(How something strange has been published. The first word should be capitalized.)*


**journalName** #Article -> http://www.w3.org/2001/XMLSchema#string *(The journal in which an article is published.)*


**note** #Reference -> http://www.w3.org/2001/XMLSchema#string *(Any additional information that can help the reader. The first word should be capitalized.)*


**organizationName**(*#Proceedings* | *#Manual*) -> http://www.w3.org/2001/XMLSchema#string *(The organization that sponsors a conference or that publishes a manual.)*


**publisherName** #Reference -> http://www.w3.org/2001/XMLSchema#string *(The publisher's name.)*


**schoolName**(*#Academic* | *#LectureNotes*) -> http://www.w3.org/2001/XMLSchema#string *(The name of the school where a thesis was written.)*


**series** #Reference -> http://www.w3.org/2001/XMLSchema#string *(The name of a series or set of books. When citing an entire book, the the title field gives its title and an optional series field gives the name of a series or multi-volume set in which the book is published.)*


**title** #Reference -> http://www.w3.org/2001/XMLSchema#string *(The work's title, typed as explained in the LaTeX book.)*


**type**(*#Chapter* | *#TechReport* | *#Academic*) -> http://www.w3.org/2001/XMLSchema#string *(The type of a technical report (for example, "Research Note").)*


**affiliation** #Reference -> http://www.w3.org/2001/XMLSchema#string *(The authors affiliation.)*


**abstract** #Reference -> http://www.w3.org/2001/XMLSchema#string *(An abstract of the work.)*


**contents** #Reference -> http://www.w3.org/2001/XMLSchema#string *(A Table of Contents.)*


**copyright** #Reference -> http://www.w3.org/2001/XMLSchema#string *(Copyright information.)*


**isbn** #Reference -> http://www.w3.org/2001/XMLSchema#string *(The International Standard Book Number.)*


**issn** #Reference -> http://www.w3.org/2001/XMLSchema#string *(The International Standard Serial Number. Used to identify a journal.)*


**keywords** #Reference -> http://www.w3.org/2001/XMLSchema#string *(Key words used for searching or possibly for annotation.)*


**language** #Reference -> http://www.w3.org/2001/XMLSchema#language *(The language in which the referenced publication is written or performed.)*


**lccn** #Reference -> http://www.w3.org/2001/XMLSchema#string *(The Library of Congress Call Number.)*


**mrNumber** #Reference -> http://www.w3.org/2001/XMLSchema#string *(The Mathematical Reviews number.)*


**price** #Reference -> http://www.w3.org/2001/XMLSchema#string *(The price of the document.)*


**size** #Reference -> http://www.w3.org/2001/XMLSchema#string *(The physical dimensions of a work.)*


**url** #Reference -> http://www.w3.org/2001/XMLSchema#string *(The WWW Universal Resource Locator that points to the item being referenced. This often is used for technical reports to point to the ftp or web site where the postscript source of the report is located.)*


**name**\_ -> http://www.w3.org/2001/XMLSchema#string *()*


**shortName**\_ -> http://www.w3.org/2001/XMLSchema#string *()*


**chapter** #Part -> http://www.w3.org/2001/XMLSchema#string *(A chapter (or section or whatever) number.)*


**numberOrVolume** http://www.w3.org/2002/07/owl#Thing -> \_ *(The number of a journal, magazine, technical report, or of a work in a series. An issue of a journal or magazine is usually identified by its volume and number; the organization that issues a technical report usually gives it a number; and sometimes books are given numbers in a named series.)*
:   **number** #Reference -> http://www.w3.org/2001/XMLSchema#string *(The number of a journal, magazine, technical report, or of a work in a series. An issue of a journal or magazine is usually identified by its volume and number; the organization that issues a technical report usually gives it a number; and sometimes books are given numbers in a named series.)*


    **issue** #Reference -> http://www.w3.org/2001/XMLSchema#string *(The issue of a conference.)*


    **volume** #Reference -> http://www.w3.org/2001/XMLSchema#nonNegativeInteger *(The volume of a journal or multivolume book.)*

**year** #Reference -> http://www.w3.org/2001/XMLSchema#gYear *()*


**month** #Reference -> http://www.w3.org/2001/XMLSchema#gMonth *()*


**day** #Reference -> http://www.w3.org/2001/XMLSchema#gDay *()*


**city** #Reference -> http://www.w3.org/2001/XMLSchema#string *()*


**state** #Reference -> http://www.w3.org/2001/XMLSchema#string *()*


**country** #Reference -> http://www.w3.org/2001/XMLSchema#string *(Usually in ISO format)*


**startPage** #Reference -> http://www.w3.org/2001/XMLSchema#nonNegativeInteger *(The beginning of a range of pages.)*


**endPage** #Reference -> http://www.w3.org/2001/XMLSchema#nonNegativeInteger *(The end of a range of pages.)*

## Individuals

<rdf:List@ttp://www.w3.org/1999/02/22-rdf-syntax-ns#nil>


<foaf:Person@a04570373>
:   - rdfs:label = 'John-Jules Meyer'
    - foaf:name = 'John-Jules Meyer'
    - foaf:firstName = 'John-Jules'
    - lastName = 'Meyer'

<foaf:Person@a43836633>
:   - rdfs:label = 'Jeen Broekstra'
    - foaf:name = 'Jeen Broekstra'
    - foaf:firstName = 'Jeen'
    - lastName = 'Broekstra'

<foaf:Person@a85228505>
:   - rdfs:label = 'Alexander Mädche'
    - foaf:name = 'Alexander Mäadche'
    - foaf:firstName = 'Alexander'
    - lastName = 'Mädche'

<foaf:Person@a48552212>
:   - rdfs:label = 'Björn Schnizler'
    - foaf:name = 'Björn Schnizler'
    - foaf:firstName = 'Björn'
    - lastName = 'Schnizler'

<foaf:Person@a971541439>
:   - rdfs:label = 'Alberto Trombetta'
    - foaf:name = 'Alberto Trombetta'
    - foaf:firstName = 'Alberto'
    - lastName = 'Trombetta'

<foaf:Person@a11090777>
:   - rdfs:label = 'Christine Parent'
    - foaf:name = 'Christine Parent'
    - foaf:firstName = 'Christine'
    - lastName = 'Parent'

<foaf:Person@a250331360>
:   - rdfs:label = 'R. Schmidt'
    - foaf:name = 'R. Schmidt'
    - foaf:firstName = 'R.'
    - lastName = 'Schmidt'

<foaf:Person@a79573306>
:   - rdfs:label = 'York Sure'
    - foaf:name = 'York Sure'
    - foaf:firstName = 'York'
    - lastName = 'Sure'

<foaf:Person@a885257047>
:   - rdfs:label = 'M. Punceva'
    - foaf:name = 'M. Punceva'
    - foaf:firstName = 'M.'
    - lastName = 'Punceva'

<foaf:Person@a74993404>
:   - rdfs:label = 'I. V. Levenshtein'
    - foaf:name = 'I. V. Levenshtein'
    - foaf:firstName = 'I. V.'
    - lastName = 'Levenshtein'

<foaf:Person@a71003986>
:   - rdfs:label = 'Steffen Staab'
    - foaf:name = 'Steffen Staab'
    - foaf:firstName = 'Steffen'
    - lastName = 'Staab'

<foaf:Person@a572406328>
:   - rdfs:label = 'Frank Boer'
    - foaf:name = 'Frank Boer'
    - foaf:firstName = 'Frank'
    - lastName = 'Boer'

<foaf:Person@a139477786>
:   - rdfs:label = 'Maarten Menken'
    - foaf:name = 'Maarten Menken'
    - foaf:firstName = 'Maarten'
    - lastName = 'Menken'

<foaf:Person@a337716610>
:   - rdfs:label = 'Manfred Hauswirth'
    - foaf:name = 'Manfred Hauswirth'
    - foaf:firstName = 'Manfred'
    - lastName = 'Hauswirth'

<foaf:Person@a086379337>
:   - rdfs:label = 'Wiebe Hoek'
    - foaf:name = 'Wiebe Hoek'
    - foaf:firstName = 'Wiebe'
    - lastName = 'Hoek'

<foaf:Person@a712561038>
:   - rdfs:label = 'Marc Ehrig'
    - foaf:name = 'Marc Ehrig'
    - foaf:firstName = 'Marc'
    - lastName = 'Ehrig'

<foaf:Person@a066600210>
:   - rdfs:label = 'Danilo Montesi'
    - foaf:name = 'Danilo Montesi'
    - foaf:firstName = 'Danilo'
    - lastName = 'Montesi'

<foaf:Person@a093016135>
:   - rdfs:label = 'Rogier Eijk'
    - foaf:name = 'Rogier Eijk'
    - foaf:firstName = 'Rogier'
    - lastName = 'Eijk'

<foaf:Person@a944339054>
:   - rdfs:label = 'Frank van Harmelen'
    - foaf:name = 'Frank van Harmelen'
    - foaf:firstName = 'Frank'
    - lastName = 'van Harmelen'

<foaf:Person@a98078619>
:   - rdfs:label = 'Philippe Cudré-Mauroux'
    - foaf:name = 'Philippe Cudré-Mauroux'
    - foaf:firstName = 'Philippe'
    - lastName = 'Cudré-Mauroux'

<foaf:Person@a39510672>
:   - rdfs:label = 'Z. Despotovic'
    - foaf:name = 'Z. Despotovic'
    - foaf:firstName = 'Z.'
    - lastName = 'Despotovic'

<foaf:Person@a431956276>
:   - rdfs:label = 'Stefano Spaccapietra'
    - foaf:name = 'Stefano Spaccapietra'
    - foaf:firstName = 'Stefano'
    - lastName = 'Spaccapietra'

<foaf:Person@a431956276b>
:   - rdfs:label = 'Mike Papazoglou'
    - foaf:name = 'Mike Papazoglou'
    - foaf:firstName = 'Mike'
    - lastName = 'Papazoglou'

<foaf:Person@a431956276c>
:   - rdfs:label = 'Zahir Tari'
    - foaf:name = 'Zahir Tari'
    - foaf:firstName = 'Zahir'
    - lastName = 'Tari'

<foaf:Person@a70955601>
:   - rdfs:label = 'A. Datta'
    - foaf:name = 'A. Datta'
    - foaf:firstName = 'A.'
    - lastName = 'Datta'

<foaf:Person@a467748807>
:   - rdfs:label = 'Ateret Anaby-Tavor'
    - foaf:name = 'Ateret Anaby-Tavor'
    - foaf:firstName = 'Ateret'
    - lastName = 'Anaby-Tavor'

<foaf:Person@a3105947>
:   - rdfs:label = 'Ronny Siebes'
    - foaf:name = 'Ronny Siebes'
    - foaf:firstName = 'Ronny'
    - lastName = 'Siebes'

<foaf:Person@a29105611>
:   - rdfs:label = 'Karl Aberer'
    - foaf:name = 'Karl Aberer'
    - foaf:firstName = 'Karl'
    - lastName = 'Aberer'

<foaf:Person@a958684218>
:   - rdfs:label = 'Peter Mika'
    - foaf:name = 'Peter Mika'
    - foaf:firstName = 'Peter'
    - lastName = 'Mika'

<foaf:Person@a94533498>
:   - rdfs:label = 'Peter Haase'
    - foaf:name = 'Peter Haase'
    - foaf:firstName = 'Peter'
    - lastName = 'Haase'

<foaf:Person@a900366022>
:   - rdfs:label = 'Avigdor Gal'
    - foaf:name = 'Avigdor Gal'
    - foaf:firstName = 'Avigdor'
    - lastName = 'Gal'

<Proceedings@a060097576>
:   - rdfs:label = 'Proceedings of the SemPGrid 04 Workshop'
    - title = 'Proceedings of the SemPGrid 04 Workshop'
    - year = '2004'
    - eventName = 'SemPGrid 04 Workshop'
    - city = 'New-York'
    - state = 'NY'
    - country = 'US'
    - month = '--05'

<InProceedings@a64263824>
:   - rdfs:label = 'Bibster - A Semantics-Based Bibliographic Peer-to-Peer System'
    - author =

      <PersonList@>
      :   - rdf:first = <\_@#a43836633>
          - rdf:rest =

            <PersonList@>
            :   - rdf:first = <\_@#a712561038>
                - rdf:rest =

                  <PersonList@>
                  :   - rdf:first = <\_@#a94533498>
                      - rdf:rest =

                        <PersonList@>
                        :   - rdf:first = <\_@#a944339054>
                            - rdf:rest =

                              <PersonList@>
                              :   - rdf:first = <\_@#a139477786>
                                  - rdf:rest =

                                    <PersonList@>
                                    :   - rdf:first = <\_@#a958684218>
                                        - rdf:rest =

                                          <PersonList@>
                                          :   - rdf:first = <\_@#a48552212>
                                              - rdf:rest =

                                                <PersonList@>
                                                :   - rdf:first = <\_@#a3105947>
                                                    - rdf:rest = <\_@http://www.w3.org/1999/02/22-rdf-syntax-ns#nil>
    - proceedings = <\_@#a060097576>
    - title = 'Bibster - A Semantics-Based Bibliographic Peer-to-Peer System'

<InProceedings@a439508789>
:   - rdfs:label = 'Measuring Similarity between Ontologies'
    - author =

      <PersonList@>
      :   - rdf:first = <\_@#a85228505>
          - rdf:rest =

            <PersonList@>
            :   - rdf:first = <\_@#a71003986>
                - rdf:rest = <\_@http://www.w3.org/1999/02/22-rdf-syntax-ns#nil>
    - proceedings = <\_@#a72192307>
    - title = 'Measuring Similarity between Ontologies'

<Article@a492378321>
:   - rdfs:label = '{P-Grid: A Self-organizing Structured P2P System}'
    - author =

      <PersonList@>
      :   - rdf:first = <\_@#a29105611>
          - rdf:rest =

            <PersonList@>
            :   - rdf:first = <\_@#a98078619>
                - rdf:rest =

                  <PersonList@>
                  :   - rdf:first = <\_@#a70955601>
                      - rdf:rest =

                        <PersonList@>
                        :   - rdf:first = <\_@#a39510672>
                            - rdf:rest =

                              <PersonList@>
                              :   - rdf:first = <\_@#a337716610>
                                  - rdf:rest =

                                    <PersonList@>
                                    :   - rdf:first = <\_@#a885257047>
                                        - rdf:rest =

                                          <PersonList@>
                                          :   - rdf:first = <\_@#a250331360>
                                              - rdf:rest = <\_@http://www.w3.org/1999/02/22-rdf-syntax-ns#nil>
    - journalName = 'ACM SIGMOD Record'
    - title = '{P-Grid}: A Self-organizing Structured P2P System'
    - year = '2003'

<Article@a475526642>
:   - rdfs:label = 'Binary Codes capable of correcting deletions, insertions, and reversals'
    - author =

      <PersonList@>
      :   - rdf:first = <\_@#a74993404>
          - rdf:rest = <\_@http://www.w3.org/1999/02/22-rdf-syntax-ns#nil>
    - journalName = 'Cybernetics and Control Theory'
    - title = 'Binary Codes capable of correcting deletions, insertions, and reversals'
    - year = '1996'

<InBook@a71568377>
:   - rdfs:label = 'Database integration: the key to data interoperability'
    - author =

      <PersonList@>
      :   - rdf:first = <\_@#a11090777>
          - rdf:rest =

            <PersonList@>
            :   - rdf:first = <\_@#a431956276>
                - rdf:rest = <\_@http://www.w3.org/1999/02/22-rdf-syntax-ns#nil>
    - book = <\_@#a108048723>
    - title = 'Database integration: the key to data interoperability'
    - editor =

      <PersonList@>
      :   - rdf:first = <\_@#a431956276>
          - rdf:rest =

            <PersonList@>
            :   - rdf:first = <\_@#a431956276b>
                - rdf:rest =

                  <PersonList@>
                  :   - rdf:first = <\_@#a431956276c>
                      - rdf:rest = <\_@http://www.w3.org/1999/02/22-rdf-syntax-ns#nil>

<Proceedings@a72192307>
:   - rdfs:label = 'Proc. Of the 13th Int. Conference on Knowledge Engineering and Management (EKAW-2002)'
    - publisherName = 'Springer-Verlag'
    - city = 'Heidelberg'
    - country = 'DE'
    - title = 'Proc. Of the 13th Int. Conference on Knowledge Engineering and Management (EKAW-2002)'
    - eventName = 'Int. Conference on Knowledge Engineering and Management'
    - shortName = 'EKAW'
    - issue = '13'
    - month = '--10'
    - year = '2002'

<Proceedings@a32071928>
:   - rdfs:label = 'Proceedings of the First European Semantic Web Symposium'
    - publisherName = 'Springer-Verlag'
    - eventName = 'European Semantic Web Symposium'
    - shortName = 'ESWS'
    - issue = '1'
    - city = ' rdf:datatype="http://www.w3.org/2001/XMLSchema#string">Heraklion'
    - country = 'GR'
    - month = '--05'
    - title = 'Proceedings of the First European Semantic Web Symposium'
    - year = '2004'

<Misc@a140583454>
:   - rdfs:label = '{QOM} - Quick Ontology Mapping'
    - author =

      <PersonList@>
      :   - rdf:first = <\_@#a712561038>
          - rdf:rest =

            <PersonList@>
            :   - rdf:first = <\_@#a71003986>
                - rdf:rest = <\_@http://www.w3.org/1999/02/22-rdf-syntax-ns#nil>
    - title = '{QOM} - Quick Ontology Mapping'
    - note = 'submitted to the ISWC 04'
    - year = '2004'

<InProceedings@a11065952>
:   - rdfs:label = 'Ontology Mapping - An Integrated Approach'
    - author =

      <PersonList@>
      :   - rdf:first = <\_@#a712561038>
          - rdf:rest =

            <PersonList@>
            :   - rdf:first = <\_@#a79573306>
                - rdf:rest = <\_@http://www.w3.org/1999/02/22-rdf-syntax-ns#nil>
    - proceedings = <\_@#a32071928>
    - title = 'Ontology Mapping - An Integrated Approach'
    - url = 'http://www.aifb.uni-karlsruhe.de/WBS/meh/publications/ehrig04ontology\_ESWS04.pdf'

<Article@a80299267>
:   - rdfs:label = 'Start making sense: The Chatty Web approach for global semantic agreements'
    - author =

      <PersonList@>
      :   - rdf:first = <\_@#a29105611>
          - rdf:rest =

            <PersonList@>
            :   - rdf:first = <\_@#a98078619>
                - rdf:rest =

                  <PersonList@>
                  :   - rdf:first = <\_@#a337716610>
                      - rdf:rest = <\_@http://www.w3.org/1999/02/22-rdf-syntax-ns#nil>
    - journalName = 'Journal of Web Semantics'
    - shortName = 'JWS'
    - title = 'Start making sense: The Chatty Web approach for global semantic agreements'
    - month = '--12'
    - year = '2003'

<Monograph@a108048723>
:   - rdfs:label = 'Object-Oriented Data Modeling'
    - publisherName = 'The MIT Press, Cambridge (MA US)'
    - city = 'Cambridge'
    - state = 'MA'
    - country = 'US'
    - title = 'Object-Oriented Data Modeling'
    - year = '2000'

<Article@a456080390>
:   - rdfs:label = 'On dynamically generated ontology translators in agent communication'
    - author =

      <PersonList@>
      :   - rdf:first = <\_@#a093016135>
          - rdf:rest =

            <PersonList@>
            :   - rdf:first = <\_@#a572406328>
                - rdf:rest =

                  <PersonList@>
                  :   - rdf:first = <\_@#a086379337>
                      - rdf:rest =

                        <PersonList@>
                        :   - rdf:first = <\_@#a04570373>
                            - rdf:rest = <\_@http://www.w3.org/1999/02/22-rdf-syntax-ns#nil>
    - journalName = 'International journal of intelligent system'
    - shortName = 'IJIS'
    - title = 'On dynamically generated ontology translators in agent communication'
    - startPage = '587'
    - endPage = '607'
    - month = '--12'
    - year = '2001'

<Article@a846015923>
:   - rdfs:label = 'A Framework for Modeling and Evaluating Automatic Semantic Reconciliation'
    - author =

      <PersonList@>
      :   - rdf:first = <\_@#a900366022>
          - rdf:rest =

            <PersonList@>
            :   - rdf:first = <\_@#a467748807>
                - rdf:rest =

                  <PersonList@>
                  :   - rdf:first = <\_@#a971541439>
                      - rdf:rest =

                        <PersonList@>
                        :   - rdf:first = <\_@#a066600210>
                            - rdf:rest = <\_@http://www.w3.org/1999/02/22-rdf-syntax-ns#nil>
    - journalName = 'VLDB Journal'
    - title = 'A Framework for Modeling and Evaluating Automatic Semantic Reconciliation'
    - note = 'to appear'
    - year = '2004'

---

Generated by OWL2HTML
