# Supplementary material for: Matching sensor ontologies through siamese neural networks without using reference alignment
Source: PeerJ Comput Sci. 2021 Jun 18;7:e602. doi: 10.7717/peerj-cs.602 (PMC8237319; doi:10.7717/peerj-cs.602)
Supplement: Supplemental Information 1 [file peerj-cs-07-602-s001.zip › 240/onto.html]

Bibliographic references


# Bibliographic references

Bibliographic references in OWL

*Possible ontology to describe bibTeX entries.*  
Author: Nick Knouf <nknouf@mit.edu>  
Contributor: Antoine Zimmermann <antoine.zimmermann@inrialpes.fr>, Jérôme Euzenat,   
Date: 08/06/2005  
Version: $Id: onto-exp.rdf,v 1.10 2008/05/27 14:40:02 euzenat Exp $

## Classes

**http://www.w3.org/1999/02/22-rdf-syntax-ns#List** (, *)*


**http://xmlns.com/foaf/0.1/Person** (, *)*


**http://xmlns.com/foaf/0.1/Organization** (, *)*


**Resource** (Resource, *)*
:   **Reference** (Reference, *Base class for all entries)*
    :   **Text** (Text, *Textual work.)*
        :   **Book** (Book, *A book that may be a monograph or a collection of written texts.)*
            :   **Monograph** (Monograph, *A book that is a single entity, as opposed to a collection.)*


                **Collection** (Collection, *A book that is collection of texts or articles.)*


                **Proceedings** (Proceedings, *The proceedings of a conference.)*

            **Informal** (Informal, *A document that was informally published or not published.)*
            :   **Booklet** (Booklet, *A work that is printed and bound, but without a named publisher or sponsoring institution.)*


                **LectureNotes** (LectureNotes, *Lecture notes.)*


                **Manual** (Manual, *Technical documentation.)*
                :   **UserGuide** (User's guide, *A manual for helping using a technology.)*


                    **ReferenceManual** (Reference manual, *A complete manual for helping using a technology.)*

                **Unpublished** (Unpublished, *A document having an author and title, but not formally published.)*

            **Part** (Part, *A part of something (either Book or Proceedings).)*
            :   **JournalPart** (Paper published in a periodical publication., *An subpart of a journal or magazine.)*
                :   **Article** (Article, *An article from a journal or magazine.)*


                    **Review** (Review, *The review of a work in a periodic publication.)*


                    **Editorial** (The editor part (foreword) of a journal issue., *An introductory part of a Journal.)*


                    **Letter** (Lettre, *A letter published in a periodic publication.)*

                **Chapter** (BookPart, *A chapter (or section or whatever) of a book having its own title.)*


                **InBook** (InBook, *A subpart of a book given by a range of pages.)*


                **InCollection** (Incollection, *A part of a book having its own title.)*


                **InProceedings** (InProceedings, *An article in a conference proceedings.)*

            **Academic** (Academic, *A Master's or PhD thesis.)*
            :   **MastersThesis** (MastersThesis, *A Master's thesis.)*


                **PhdThesis** (PhdThesis, *A PhD thesis.)*

            **Misc** (Misc, *Use this type when nothing else fits.)*


            **Report** (Report, *A report published by an institution with some explicit policy.)*
            :   **InstitutionReport** (Institution report, *A report published by an institution.)*
                :   **ProspectiveReport** (Prospective report, *A prospective report on a particular topic or field.)*


                    **EvaluationReport** (Evaluation report, *A report evaluating the activity of some institution subpart.)*


                    **SerialReport** (Serial report, *A report published by an institution as part of a serie.)*
                    :   **NormalizationReport** (Normalization report, *A report constituting a normative document.)*
                        :   **Standard** (Standard, *A document describing a standard.)*


                            **Recommendation** (Recommendation, *A document describing a recommended technology.)*

                        **WorkReport** (Work report, *A report on technical matter published within a series.)*
                        :   **TechReport** (Technical report, *A report on technical matter published within a series.)*


                            **TechnicalMemo** (Technical memorandum, *)*


                            **ResearchReport** (Research report, *)*


                            **ResearchNote** (Research note, *)*

                    **PeriodicReport** (Institution report, *A report published by an institution on a regular basis.)*
                    :   **YearlyReport** (Institution report, *A report published by an institution on a regular basis.)*

                **Deliverable** (Deliverable report, *A report delivered for accomplishing a contract.)*
                :   **FinalReport** (Final report, *The final report on a contract.)*

        **MotionPicture** (MotionPicture, *A film/movie/motion picture.)*

**Periodical** (Journal or magazine, *A periodical publication collecting works from different authors.)*
:   **Journal** (Journal, *A periodical publication of peer-reviewed scientific papers.)*


    **Magazine** (Magazine, *A periodical publication of scientific papers and news.)*

**ScientificMeeting** (Scientific meeting, *An event presenting work.)*
:   **Conference** (Conference, *A scientific conference.)*


    **Congress** (Congress, *A scientific congress.)*


    **Symposium** (Symposium, *A symposium.)*


    **Workshop** (Workshop, *A scientific workshop, i.e. a small audience conference in which more interaction between participants can occur.)*

**Address** (Address, *The street address of the location of some organization or event.)*


**Institution** (Institution, *An institution.)*
:   super: *http://xmlns.com/foaf/0.1/Organization*  


    **Society** (Society, *A scientific society which can additionnaly publish books and journals.)*


    **Publisher** (Publisher, *The publisher of books or journals.)*


    **HigherEducationInstitution** (Higher education institution, *A school or university.)*
    :   **University** (University, *A University.)*


        **School** (School, *A identified and autonomous university department, college or school.)*


        **EngineerSchool** (Engineer school, *A French-system so-called Engineer school or German technische Hochschule.)*


        **Polytechnics** (Practical university, *The French CNAM or Britsh Polytechnics.)*

**PersonList** (Person list, *A list of persons.)*
:   super: *http://www.w3.org/1999/02/22-rdf-syntax-ns#List*

**PageRange** (PageRange, *A range of pages.)*


**Date** (Date, *Date of a day which can be unknown (i.e., only the year is known or only the year and month). This is for overcoming the limits of XML-Schema for wich a date is not separable.)*

## Properties

**http://www.w3.org/1999/02/22-rdf-syntax-ns#first**: http://www.w3.org/1999/02/22-rdf-syntax-ns#List -> \_ *()*


**http://www.w3.org/1999/02/22-rdf-syntax-ns#rest**: http://www.w3.org/1999/02/22-rdf-syntax-ns#List -> http://www.w3.org/1999/02/22-rdf-syntax-ns#List *()*

**http://purl.org/dc/elements/1.1/creator**\_ -> \_ *()*


**http://purl.org/dc/elements/1.1/contributor**\_ -> \_ *()*


**http://purl.org/dc/elements/1.1/description**\_ -> \_ *()*


**http://purl.org/dc/elements/1.1/date**\_ -> \_ *()*


**http://xmlns.com/foaf/0.1/firstName**\_ -> \_ *()*


**#lastName**\_ -> \_ *()*


**http://xmlns.com/foaf/0.1/name**\_ -> \_ *()*

## Individuals

<rdf:List@ttp://www.w3.org/1999/02/22-rdf-syntax-ns#nil>


<foaf:Person@a04570373>
:   - rdfs:label = 'John-Jules Meyer'
    - foaf:name = 'John-Jules Meyer'
    - foaf:firstName = 'John-Jules'

<foaf:Person@a43836633>
:   - rdfs:label = 'Jeen Broekstra'
    - foaf:name = 'Jeen Broekstra'
    - foaf:firstName = 'Jeen'

<foaf:Person@a85228505>
:   - rdfs:label = 'Alexander Mädche'
    - foaf:name = 'Alexander Mädche'
    - foaf:firstName = 'Alexander'

<foaf:Person@a48552212>
:   - rdfs:label = 'Björn Schnizler'
    - foaf:name = 'Björn Schnizler'
    - foaf:firstName = 'Björn'

<foaf:Person@a971541439>
:   - rdfs:label = 'Alberto Trombetta'
    - foaf:name = 'Alberto Trombetta'
    - foaf:firstName = 'Alberto'

<foaf:Person@a11090777>
:   - rdfs:label = 'Christine Parent'
    - foaf:name = 'Christine Parent'
    - foaf:firstName = 'Christine'

<foaf:Person@a250331360>
:   - rdfs:label = 'R. Schmidt'
    - foaf:name = 'R. Schmidt'
    - foaf:firstName = 'R.'

<foaf:Person@a79573306>
:   - rdfs:label = 'York Sure'
    - foaf:name = 'York Sure'
    - foaf:firstName = 'York'

<foaf:Person@a885257047>
:   - rdfs:label = 'M. Punceva'
    - foaf:name = 'M. Punceva'
    - foaf:firstName = 'M.'

<foaf:Person@a74993404>
:   - rdfs:label = 'I. V. Levenshtein'
    - foaf:name = 'I. V. Levenshtein'
    - foaf:firstName = 'I. V.'

<foaf:Person@a71003986>
:   - rdfs:label = 'Steffen Staab'
    - foaf:name = 'Steffen Staab'
    - foaf:firstName = 'Steffen'

<foaf:Person@a572406328>
:   - rdfs:label = 'Frank Boer'
    - foaf:name = 'Frank Boer'
    - foaf:firstName = 'Frank'

<foaf:Person@a139477786>
:   - rdfs:label = 'Maarten Menken'
    - foaf:name = 'Maarten Menken'
    - foaf:firstName = 'Maarten'

<foaf:Person@a337716610>
:   - rdfs:label = 'Manfred Hauswirth'
    - foaf:name = 'Manfred Hauswirth'
    - foaf:firstName = 'Manfred'

<foaf:Person@a086379337>
:   - rdfs:label = 'Wiebe Hoek'
    - foaf:name = 'Wiebe Hoek'
    - foaf:firstName = 'Wiebe'

<foaf:Person@a712561038>
:   - rdfs:label = 'Marc Ehrig'
    - foaf:name = 'Marc Ehrig'
    - foaf:firstName = 'Marc'

<foaf:Person@a066600210>
:   - rdfs:label = 'Danilo Montesi'
    - foaf:name = 'Danilo Montesi'
    - foaf:firstName = 'Danilo'

<foaf:Person@a093016135>
:   - rdfs:label = 'Rogier Eijk'
    - foaf:name = 'Rogier Eijk'
    - foaf:firstName = 'Rogier'

<foaf:Person@a944339054>
:   - rdfs:label = 'Frank van Harmelen'
    - foaf:name = 'Frank van Harmelen'
    - foaf:firstName = 'Frank'

<foaf:Person@a98078619>
:   - rdfs:label = 'Philippe Cudré-Mauroux'
    - foaf:name = 'Philippe Cudré-Mauroux'
    - foaf:firstName = 'Philippe'

<foaf:Person@a39510672>
:   - rdfs:label = 'Z. Despotovic'
    - foaf:name = 'Z. Despotovic'
    - foaf:firstName = 'Z.'

<foaf:Person@a431956276>
:   - rdfs:label = 'Stefano Spaccapietra'
    - foaf:name = 'Stefano Spaccapietra'
    - foaf:firstName = 'Stefano'

<foaf:Person@a431956276b>
:   - rdfs:label = 'Mike Papazoglou'
    - foaf:name = 'Mike Papazoglou'
    - foaf:firstName = 'Mike'

<foaf:Person@a431956276c>
:   - rdfs:label = 'Zahir Tari'
    - foaf:name = 'Zahir Tari'
    - foaf:firstName = 'Zahir'

<foaf:Person@a70955601>
:   - rdfs:label = 'A. Datta'
    - foaf:name = 'A. Datta'
    - foaf:firstName = 'A.'

<foaf:Person@a467748807>
:   - rdfs:label = 'Ateret Anaby-Tavor'
    - foaf:name = 'Ateret Anaby-Tavor'
    - foaf:firstName = 'Ateret'

<foaf:Person@a3105947>
:   - rdfs:label = 'Ronny Siebes'
    - foaf:name = 'Ronny Siebes'
    - foaf:firstName = 'Ronny'

<foaf:Person@a29105611>
:   - rdfs:label = 'Karl Aberer'
    - foaf:name = 'Karl Aberer'
    - foaf:firstName = 'Karl'

<foaf:Person@a958684218>
:   - rdfs:label = 'Peter Mika'
    - foaf:name = 'Peter Mika'
    - foaf:firstName = 'Peter'

<foaf:Person@a94533498>
:   - rdfs:label = 'Peter Haase'
    - foaf:name = 'Peter Haase'
    - foaf:firstName = 'Peter'

<foaf:Person@a900366022>
:   - rdfs:label = 'Avigdor Gal'
    - foaf:name = 'Avigdor Gal'
    - foaf:firstName = 'Avigdor'

<Journal@a246119474>
:   - rdfs:label = 'Journal of Web Semantics'
    - foaf:name = 'Journal of Web Semantics'

<Publisher@a131020767>
:   - rdfs:label = 'Springer-Verlag'

<Journal@a70981683>
:   - rdfs:label = 'Cybernetics and Control Theory'

<Publisher@a85849488>
:   - rdfs:label = 'The MIT Press'

<Journal@a362042121>
:   - rdfs:label = 'International journal of intelligent system'

<Journal@a674639524>
:   - rdfs:label = 'ACM SIGMOD Record'

<Journal@a906774044>
:   - rdfs:label = 'VLDB Journal'

<Workshop@spg04>
:   - rdfs:label = 'SemPGrid 04 Workshop'

<Conference@a72192307c>
:   - rdfs:label = '13th Int. Conference on Knowledge Engineering and Management (EKAW-2002)'

<Conference@a32071928c>
:   - rdfs:label = 'First European Semantic Web Symposium'

<Proceedings@a060097576>
:   - rdfs:label = 'Proceedings of the SemPGrid 04 Workshop'

<InProceedings@a64263824>
:   - rdfs:label = 'Bibster - A Semantics-Based Bibliographic Peer-to-Peer System'

<InProceedings@a439508789>
:   - rdfs:label = 'Measuring Similarity between Ontologies'

<Article@a492378321>
:   - rdfs:label = '{P-Grid: A Self-organizing Structured P2P System}'

<Article@a475526642>
:   - rdfs:label = 'Binary Codes capable of correcting deletions, insertions, and reversals'

<InBook@a71568377>
:   - rdfs:label = 'Database integration: the key to data interoperability'

<Proceedings@a72192307>
:   - rdfs:label = 'Proc. Of the 13th Int. Conference on Knowledge Engineering and Management (EKAW-2002)'

<Proceedings@a32071928>
:   - rdfs:label = 'Proceedings of the First European Semantic Web Symposium'

<Misc@a140583454>
:   - rdfs:label = '{QOM} - Quick Ontology Mapping'

<InProceedings@a11065952>
:   - rdfs:label = 'Ontology Mapping - An Integrated Approach'

<Article@a80299267>
:   - rdfs:label = 'Start making sense: The Chatty Web approach for global semantic agreements'

<Monograph@a108048723>
:   - rdfs:label = 'Object-Oriented Data Modeling'

<Article@a456080390>
:   - rdfs:label = 'On dynamically generated ontology translators in agent communication'

<Article@a846015923>
:   - rdfs:label = 'A Framework for Modeling and Evaluating Automatic Semantic Reconciliation'

---

Generated by OWL2HTML
