# Supplementary material for: Matching sensor ontologies through siamese neural networks without using reference alignment
Source: PeerJ Comput Sci. 2021 Jun 18;7:e602. doi: 10.7717/peerj-cs.602 (PMC8237319; doi:10.7717/peerj-cs.602)
Supplement: Supplemental Information 1 [file peerj-cs-07-602-s001.zip › 241/onto.html]

Bibliographic references


# Bibliographic references

Bibliographic references in OWL

*Possible ontology to describe bibTeX entries.*  
Author: Nick Knouf <nknouf@mit.edu>  
Contributor: Antoine Zimmermann <antoine.zimmermann@inrialpes.fr>, Jérôme Euzenat,   
Date: 08/06/2005  
Version: $Id: onto.rdf,v 1.30 2008/05/27 14:41:13 euzenat Exp $

## Classes

**http://www.w3.org/1999/02/22-rdf-syntax-ns#List** (, *)*


**http://xmlns.com/foaf/0.1/Person** (, *)*


**http://xmlns.com/foaf/0.1/Organization** (, *)*


**Reference** (Reference, *Base class for all entries)*


**Article** (Article, *An article from a journal or magazine.)*


**Book** (Book, *A book that may be a monograph or a collection of written texts.)*


**Monograph** (Monograph, *A book that is a single entity, as opposed to a collection.)*


**Collection** (Collection, *A book that is collection of texts or articles.)*


**Informal** (Informal, *A document that was informally published or not published.)*


**Booklet** (Booklet, *A work that is printed and bound, but without a named publisher or sponsoring institution.)*


**Part** (Part, *A part of something (either Book or Proceedings).)*


**Chapter** (BookPart, *A chapter (or section or whatever) of a book having its own title.)*


**InBook** (InBook, *A subpart of a book given by a range of pages.)*


**InCollection** (Incollection, *A part of a book having its own title.)*


**InProceedings** (InProceedings, *An article in a conference proceedings.)*


**LectureNotes** (LectureNotes, *Lecture notes.)*


**Manual** (Manual, *Technical documentation.)*


**Academic** (Academic, *A Master's or PhD thesis.)*


**MastersThesis** (MastersThesis, *A Master's thesis.)*


**PhdThesis** (PhdThesis, *A PhD thesis.)*


**Misc** (Misc, *Use this type when nothing else fits.)*


**Proceedings** (Proceedings, *The proceedings of a conference.)*


**Report** (Report, *A report published by an institution with some explicit policy.)*


**TechReport** (TechReport, *A report published by a school or other institution, usually numbered within a series.)*


**Deliverable** (Deliverable report, *A report delivered for accomplishing a contract.)*


**Unpublished** (Unpublished, *A document having an author and title, but not formally published.)*


**MotionPicture** (MotionPicture, *A film/movie/motion picture.)*


**Journal** (Journal or magazine, *A periodical publication collecting works from different authors.)*


**Conference** (The location of an event, *An event presenting work.)*


**Address** (Address, *The street address of the location of some organization or event.)*


**Institution** (Institution, *An institution.)*


**Publisher** (Publisher, *The publisher of books or journals.)*


**School** (School, *A school or university.)*


**PersonList** (Person list, *A list of persons.)*


**PageRange** (PageRange, *A range of pages.)*


**Date** (Date, *Date of a day which can be unknown (i.e., only the year is known or only the year and month). This is for overcoming the limits of XML-Schema for wich a date is not separable.)*

## Properties

**http://www.w3.org/1999/02/22-rdf-syntax-ns#first**: http://www.w3.org/1999/02/22-rdf-syntax-ns#List -> \_ *()*


**http://www.w3.org/1999/02/22-rdf-syntax-ns#rest**: http://www.w3.org/1999/02/22-rdf-syntax-ns#List -> http://www.w3.org/1999/02/22-rdf-syntax-ns#List *()*

**http://purl.org/dc/elements/1.1/creator**\_ -> \_ *()*


**http://purl.org/dc/elements/1.1/contributor**\_ -> \_ *()*


**http://purl.org/dc/elements/1.1/description**\_ -> \_ *()*


**http://purl.org/dc/elements/1.1/date**\_ -> \_ *()*


**http://xmlns.com/foaf/0.1/firstName**\_ -> \_ *()*


**http://xmlns.com/foaf/0.1/name**\_ -> \_ *()*

## Individuals

<rdf:List@ttp://www.w3.org/1999/02/22-rdf-syntax-ns#nil>

---

Generated by OWL2HTML
