# Supplementary material for: Matching sensor ontologies through siamese neural networks without using reference alignment
Source: PeerJ Comput Sci. 2021 Jun 18;7:e602. doi: 10.7717/peerj-cs.602 (PMC8237319; doi:10.7717/peerj-cs.602)
Supplement: Supplemental Information 1 [file peerj-cs-07-602-s001.zip › 241/refalign.html]

# (level 0) Alignment

## Source: http://oaei.ontologymatching.org/2011/benchmarks/101/onto.rdf

## Target: http://oaei.ontologymatching.org/2011/benchmarks/241/onto.rdf

## Correspondences

PersonList = PersonList
:   1.0

Unpublished = Unpublished
:   1.0

Address = Address
:   1.0

Chapter = Chapter
:   1.0

InBook = InBook
:   1.0

Date = Date
:   1.0

PageRange = PageRange
:   1.0

Booklet = Booklet
:   1.0

LectureNotes = LectureNotes
:   1.0

MastersThesis = MastersThesis
:   1.0

TechReport = TechReport
:   1.0

Misc = Misc
:   1.0

Collection = Collection
:   1.0

Academic = Academic
:   1.0

MotionPicture = MotionPicture
:   1.0

Article = Article
:   1.0

Informal = Informal
:   1.0

Book = Book
:   1.0

School = School
:   1.0

PhdThesis = PhdThesis
:   1.0

Proceedings = Proceedings
:   1.0

Reference = Reference
:   1.0

InCollection = InCollection
:   1.0

Report = Report
:   1.0

Conference = Conference
:   1.0

Part = Part
:   1.0

Publisher = Publisher
:   1.0

Manual = Manual
:   1.0

InProceedings = InProceedings
:   1.0

Journal = Journal
:   1.0

Monograph = Monograph
:   1.0

Institution = Institution
:   1.0

Deliverable = Deliverable
:   1.0
