# Supplementary material for: Matching sensor ontologies through siamese neural networks without using reference alignment
Source: PeerJ Comput Sci. 2021 Jun 18;7:e602. doi: 10.7717/peerj-cs.602 (PMC8237319; doi:10.7717/peerj-cs.602)
Supplement: Supplemental Information 1 [file peerj-cs-07-602-s001.zip › 247/onto.html]

Bibliographic references


# Bibliographic references

Bibliographic references in OWL

*Possible ontology to describe bibTeX entries.*  
Author: Nick Knouf <nknouf@mit.edu>  
Contributor: Antoine Zimmermann <antoine.zimmermann@inrialpes.fr>, Jérôme Euzenat,   
Date: 08/06/2005  
Version: $Id: onto-exp.rdf,v 1.10 2008/05/27 14:40:02 euzenat Exp $

## Classes

**http://www.w3.org/1999/02/22-rdf-syntax-ns#List** (, *)*


**http://xmlns.com/foaf/0.1/Person** (, *)*


**http://xmlns.com/foaf/0.1/Organization** (, *)*


**Resource** (Resource, *)*
:   **Reference** (Reference, *Base class for all entries)*
    :   **Text** (Text, *Textual work.)*
        :   **Book** (Book, *A book that may be a monograph or a collection of written texts.)*
            :   **Monograph** (Monograph, *A book that is a single entity, as opposed to a collection.)*


                **Collection** (Collection, *A book that is collection of texts or articles.)*


                **Proceedings** (Proceedings, *The proceedings of a conference.)*

            **Informal** (Informal, *A document that was informally published or not published.)*
            :   **Booklet** (Booklet, *A work that is printed and bound, but without a named publisher or sponsoring institution.)*


                **LectureNotes** (LectureNotes, *Lecture notes.)*


                **Manual** (Manual, *Technical documentation.)*
                :   **UserGuide** (User's guide, *A manual for helping using a technology.)*


                    **ReferenceManual** (Reference manual, *A complete manual for helping using a technology.)*

                **Unpublished** (Unpublished, *A document having an author and title, but not formally published.)*

            **Part** (Part, *A part of something (either Book or Proceedings).)*
            :   **JournalPart** (Paper published in a periodical publication., *An subpart of a journal or magazine.)*
                :   **Article** (Article, *An article from a journal or magazine.)*


                    **Review** (Review, *The review of a work in a periodic publication.)*


                    **Editorial** (The editor part (foreword) of a journal issue., *An introductory part of a Journal.)*


                    **Letter** (Lettre, *A letter published in a periodic publication.)*

                **Chapter** (BookPart, *A chapter (or section or whatever) of a book having its own title.)*


                **InBook** (InBook, *A subpart of a book given by a range of pages.)*


                **InCollection** (Incollection, *A part of a book having its own title.)*


                **InProceedings** (InProceedings, *An article in a conference proceedings.)*

            **Academic** (Academic, *A Master's or PhD thesis.)*
            :   **MastersThesis** (MastersThesis, *A Master's thesis.)*


                **PhdThesis** (PhdThesis, *A PhD thesis.)*

            **Misc** (Misc, *Use this type when nothing else fits.)*


            **Report** (Report, *A report published by an institution with some explicit policy.)*
            :   **InstitutionReport** (Institution report, *A report published by an institution.)*
                :   **ProspectiveReport** (Prospective report, *A prospective report on a particular topic or field.)*


                    **EvaluationReport** (Evaluation report, *A report evaluating the activity of some institution subpart.)*


                    **SerialReport** (Serial report, *A report published by an institution as part of a serie.)*
                    :   **NormalizationReport** (Normalization report, *A report constituting a normative document.)*
                        :   **Standard** (Standard, *A document describing a standard.)*


                            **Recommendation** (Recommendation, *A document describing a recommended technology.)*

                        **WorkReport** (Work report, *A report on technical matter published within a series.)*
                        :   **TechReport** (Technical report, *A report on technical matter published within a series.)*


                            **TechnicalMemo** (Technical memorandum, *)*


                            **ResearchReport** (Research report, *)*


                            **ResearchNote** (Research note, *)*

                    **PeriodicReport** (Institution report, *A report published by an institution on a regular basis.)*
                    :   **YearlyReport** (Institution report, *A report published by an institution on a regular basis.)*

                **Deliverable** (Deliverable report, *A report delivered for accomplishing a contract.)*
                :   **FinalReport** (Final report, *The final report on a contract.)*

        **MotionPicture** (MotionPicture, *A film/movie/motion picture.)*

**Periodical** (Journal or magazine, *A periodical publication collecting works from different authors.)*
:   **Journal** (Journal, *A periodical publication of peer-reviewed scientific papers.)*


    **Magazine** (Magazine, *A periodical publication of scientific papers and news.)*

**ScientificMeeting** (Scientific meeting, *An event presenting work.)*
:   **Conference** (Conference, *A scientific conference.)*


    **Congress** (Congress, *A scientific congress.)*


    **Symposium** (Symposium, *A symposium.)*


    **Workshop** (Workshop, *A scientific workshop, i.e. a small audience conference in which more interaction between participants can occur.)*

**Address** (Address, *The street address of the location of some organization or event.)*


**Institution** (Institution, *An institution.)*
:   super: *http://xmlns.com/foaf/0.1/Organization*  


    **Society** (Society, *A scientific society which can additionnaly publish books and journals.)*


    **Publisher** (Publisher, *The publisher of books or journals.)*


    **HigherEducationInstitution** (Higher education institution, *A school or university.)*
    :   **University** (University, *A University.)*


        **School** (School, *A identified and autonomous university department, college or school.)*


        **EngineerSchool** (Engineer school, *A French-system so-called Engineer school or German technische Hochschule.)*


        **Polytechnics** (Practical university, *The French CNAM or Britsh Polytechnics.)*

**PersonList** (Person list, *A list of persons.)*
:   super: *http://www.w3.org/1999/02/22-rdf-syntax-ns#List*

**PageRange** (PageRange, *A range of pages.)*


**Date** (Date, *Date of a day which can be unknown (i.e., only the year is known or only the year and month). This is for overcoming the limits of XML-Schema for wich a date is not separable.)*

## Properties

**http://www.w3.org/1999/02/22-rdf-syntax-ns#first**: http://www.w3.org/1999/02/22-rdf-syntax-ns#List -> \_ *()*


**http://www.w3.org/1999/02/22-rdf-syntax-ns#rest**: http://www.w3.org/1999/02/22-rdf-syntax-ns#List -> http://www.w3.org/1999/02/22-rdf-syntax-ns#List *()*

**http://purl.org/dc/elements/1.1/creator**\_ -> \_ *()*


**http://purl.org/dc/elements/1.1/contributor**\_ -> \_ *()*


**http://purl.org/dc/elements/1.1/description**\_ -> \_ *()*


**http://purl.org/dc/elements/1.1/date**\_ -> \_ *()*


**http://xmlns.com/foaf/0.1/firstName**\_ -> \_ *()*


**#lastName**\_ -> \_ *()*


**http://xmlns.com/foaf/0.1/name**\_ -> \_ *()*

## Individuals

<rdf:List@ttp://www.w3.org/1999/02/22-rdf-syntax-ns#nil>

---

Generated by OWL2HTML
